# Identification of STAM-binding protein as a target for the treatment of gemcitabine resistance pancreatic cancer in a nutrient-poor microenvironment

**DOI:** 10.1038/s41419-024-07048-z

**Published:** 2024-09-06

**Authors:** Wenming Zhang, Zheng Xu, Yunyan Du, Tiande Liu, Zhijuan Xiong, Junwen Hu, Leifeng Chen, Xiaogang Peng, Fan Zhou

**Affiliations:** 1 Department of General Surgery, The Second Affiliated Hospital of Nanchang University, 1 Minde Road, Nanchang, 330006 Jiangxi PR China; 2 Department of Pharmacology, Nanchang University, 461 Bayi Avenue, Nanchang, 330006 Jiangxi PR China; 3 Department of Gastroenterology, The First Affiliated Hospital of Nanchang University, 17 Yongwaizheng Street, Nanchang, 330006 Jiangxi PR China; 4 Department of Oncology, The Second Affiliated Hospital of Nanchang University, 1 Minde Road, Nanchang, 330006 Jiangxi PR China; 5 Medical Center for Cardiovascular Diseases, Neurological Diseases and Tumors of Jiangxi Province, The Second Affiliated Hospital of Nanchang University, 1 Minde Road, Nanchang, 330006 Jiangxi PR China; 6 Laboratory of Translational Medicine, National Cancer Center/National Clinical Research Center for Cancer/Cancer Hospital, Chinese Academy of Medical Sciences and Peking Union Medical College, 100021 Beijing, PR China; 7 Jiangxi Province Key Laboratory of Molecular Medicine, The Second Affiliated Hospital of Nanchang University, 1 Minde Road, Nanchang, 330006 Jiangxi PR China

**Keywords:** Tumour biomarkers, Oncogenes

## Abstract

Pancreatic cancer (PC) is a highly malignant solid tumor whose resistance to gemcitabine (GEM) chemotherapy is a major cause of poor patient prognosis. Although PC is known to thrive on malnutrition, the mechanism underlying its chemotherapy resistance remains unclear. The current study analyzed clinical tissue sample databases using bioinformatics tools and observed significantly upregulated expression of the deubiquitinase STAMBP in PC tissues. Functional experiments revealed that STAMBP knockdown remarkably increases GEM sensitivity in PC cells. Multiple omics analyses suggested that STAMBP enhances aerobic glycolysis and suppresses mitochondrial respiration to increase GEM resistance in PC both in vitro and in vivo. STAMBP knockdown decreased PDK1 levels, an essential regulator of the aerobic glycolytic process, in several cancers. Mechanistically, STAMBP promoted the PDK1-mediated Warburg effect and chemotherapy resistance by modulating E2F1 via direct binding to E2F1 and suppressing its degradation and ubiquitination. High-throughput compound library screening using three-dimensional protein structure analysis and drug screening identified the FDA drug entrectinib as a potent GEM sensitizer and STAMBP inhibitor, augmenting the antitumor effect of GEM in a patient-derived xenograft (PDX) model. Overall, we established a novel mechanism, via the STAMBP–E2F1–PDK1 axis, by which PC cells become chemoresistant in a nutrient-poor tumor microenvironment.

## Introduction

Pancreatic cancer (PC) is a highly malignant solid tumor with a poor prognosis and a 5-year survival rate of < 10% [1]. By 2030, PC will be the second most common cause of cancer-related deaths [2]. Radical surgery is currently the most effective treatment for PC [3]. Nevertheless, the median progression-free survival time after surgery does not exceed 2 years, and the 5-year survival rate does not exceed 40%, which is far lower than that of other digestive tract tumors [4]. Chemotherapy is the preferred option for initial treatment of metastatic or locally advanced PC [5]. However, PC is prone to acquiring drug resistance, and the recurrence rate remains > 95% after surgery [6]. Although some progress has been made in understanding the mechanism of chemotherapy resistance in PC, the high rate of chemotherapy resistance remains an important factor limiting its efficacy. Therefore, continuous investigation of the mechanism of acquired chemotherapy resistance in PC and identification of new therapeutic targets and drug resistance solutions, which are of great significance for improving the prognosis of patients with PC, are considered imperative.

Aerobic glycolysis is the primary pathway through which tumor cells gain energy under oxygen-sufficient conditions [7]. Although a relatively inefficient method of gaining energy compared to mitochondrial oxidative phosphorylation, aerobic glycolysis and its branches can enhance biosynthesis, provide biological raw materials that are essential for rapid tumor cell proliferation, and facilitate tumor progression [8]. Several studies have indicated that the rate of aerobic glycolysis is linked to tumor progression and drug resistance [9]. Studies have shown that glycolysis and the pentose phosphate pathway can regulate drug resistance in tumor cells [10]. The 3-phosphate-dependent protein kinase 1 (PDK1) is a member of the AGC kinase family and is encoded by the PDPK1 gene located at 16p13.3 [11]. PDK1 is thought to be a “switch” that regulates the fate of pyruvate in glucose metabolism [12]. PDK1 acts as a critical regulator of aerobic glycolysis in tumor cells by phosphorylating the E1 subunit of pyruvate dehydrogenase (PDH) at Ser232, resulting in its inactivation. Inactivated PDH cannot promote the conversion of pyruvate to acetyl-CoA, thereby inhibiting the entry of pyruvate into the tricarboxylic acid (TCA) cycle [13, 14]. PDK1 is involved in physiological processes, such as proliferation, metastasis, stress response, apoptosis, repair of DNA damage, and resistance to chemotherapy in cancer cells [11, 15]. Recent studies have shown that aerobic glycolysis mediated by PDK1 plays a critical role in chemoresistance in numerous malignancies, including acute myeloid leukemia, breast cancer, PC, hepatocellular carcinoma, and prostate cancer [11, 16, 17]. However, the role of PDK1 in the metabolic transformation of PC cells and the specific mechanism by which PDK1 leads to chemoresistance in PC remain unclear.

Deubiquitinates (DUBs) eliminate ubiquitin from proteins, thereby antagonizing the effects of E3 ligases [18]. STAM-binding protein (STAMBP), also referred to as an associated molecule with the Src homology 3 domain of signal transducing adaptor molecule (AMSH), is a ubiquitinating enzyme in the Jab1/MPN family of metalloenzymes that modulates the intracellular stability of substrates by specifically removing the ubiquitin molecule [19, 20]. An imbalance in STAMBP is related to specific characteristics of cancer and leads to the occurrence of malignant tumors [21]. Several studies have demonstrated the role of STAMBP as an oncogene in the malignant progression of tumors [22]. Increasing evidence has shown that STAMBP is highly elevated and is linked to poor clinical prognosis in multiple cancers, such as lung adenocarcinoma, breast cancer, triple-negative breast cancer, and PC [19, 20, 22]. However, the biological function and anti-chemotherapeutic mechanism of STAMBP in PC remain unknown.

In the present study, we demonstrated the mechanism and action of STAMBP in the chemoresistance of PC to gemcitabine (GEM). First, we demonstrated high STAMBP expression in PC tissues. Second, we confirmed that STAMBP leads to chemotherapy resistance in PC by increasing PDK1-mediated aerobic glycolysis. Next, we revealed the molecular mechanism by which STAMBP stabilizes E2F1 expression by inhibiting its ubiquitination and degradation. Importantly, the current study offers preclinical evidence for the therapeutic potential of STAMBP inhibition in chemotherapy-resistant PC. We extensively characterized the inhibitory effect of entrectinib, an FDA drug, on GEM resistance in PC both in vitro and in vivo. Our findings highlighted the potential of entrectinib combination therapy, which should eventually be evaluated and optimized in clinical trials.

## Results

### STAMBP expression was upregulated and correlated with the progression and prognosis of patients with PC

**Fig. 1:**
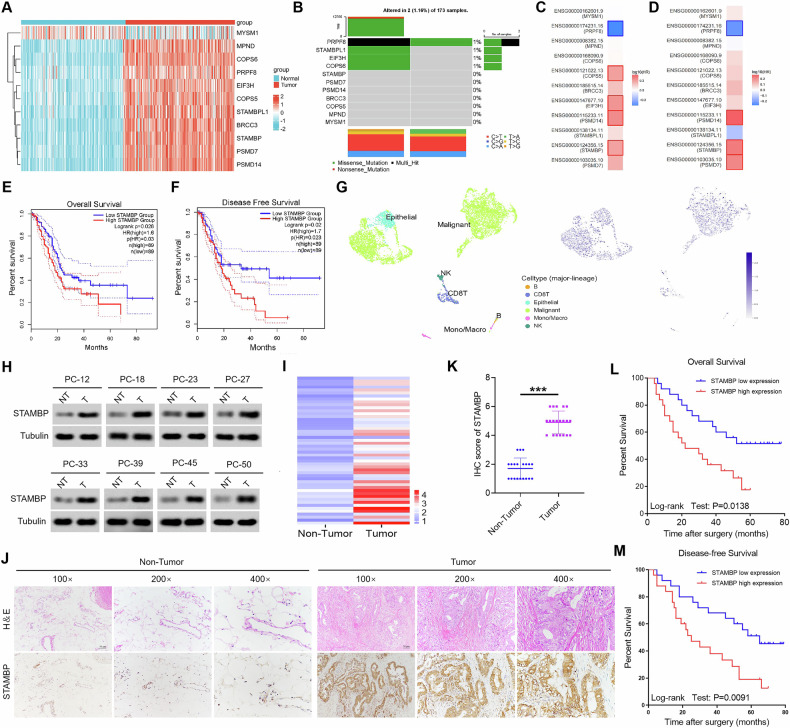
High STAMBP level was closely correlated with the poor prognosis in patients with PC. **A** The heatmap volcano plot of the 11 differentially expressed Zn^+^-dependent JAMM deubiquitinases in PC tissues and the normal tissue from the TCGA-PAAD dataset and GTEx dataset. Gene expression values are z-transformed. **B** Waterfall plots of the mutated Zn^+^-dependent JAMM deubiquitinases genes in the whole cohort. Prognosis of overall (**C**) and disease-free survival (**D**) in TCGA-PAAD dataset with differentially expressed Zn^+^-dependent JAMM deubiquitinases. Kaplan–Meier curves for predicting the overall survival (**E**) and disease-free survival (**F**) for both groups of patients with pancreatic cancer classified by high- and low STAMBP expression in TCGA-PAAD dataset. **G** Analysis of STAMBP gene expression in pancreatic cancer using the single-cell sequencing data set from the TISCH database (http://tisch.comp-genomics.org/). **H** Determination and STAMBP protein levels in PC tissues and paired normal tissues by western blotting assay. Tubulin was used as a loading control. **I** The heatmap volcano plot shown the differentially expressed STAMBP in PC tissues and paired normal tissues. Representative image (**J**) and quantification (**K**) of STAMBP staining in PC tissues and paired normal tissues. The image was captured at 100×, 200× and 400× magnification. Scale bar, 50 μm. ^***^P < 0.001. Kaplan–Meier plots representing probabilities of overall (**L**) and progression-free survival (**M**) in 128 PC patients according to expression level of STAMBP. Statistical analysis was conducted using Student’s *t* test and Log Rank test.

The expression of Zn^+^-dependent JAMM deubiquitinases is associated with the development of malignant tumors [23]. Initially, we observed the expression of 11 Zn^+^-dependent JAMM deubiquitinases in PC cells (Supplementary Table S1); the expression was significantly higher in tumor tissues than in adjacent tissue samples (Fig. 1A and Supplementary Fig. S1A). Investigation of the frequency of CNV alterations revealed a prevalence of Zn^+^-dependent JAMM deubiquitinases; EIF3H, COSP6, and MPND presented more copy-number amplifications, whereas STAMBPL1 presented a higher copy number (Supplementary Fig. S1B). The locations of CNV alterations in Zn^+^-dependent JAMM deubiquitinases on chromosomes are shown in Supplementary Fig. S1C. Subsequently, we evaluated the genomic characteristics of Zn^+^-dependent JAMM deubiquitinases and found approximately 1.16% of cancer samples (*n* = 173) to carry mutations (Fig. 1B). Next, we extracted transcriptomic data of the PC cohort from The Cancer Genome Atlas database (TCGA) public datasets to explore the predictive value of Zn^+^-dependent JAMM deubiquitinases for overall survival (OS) and disease-free survival (DFS). Specifically, STAMBP is overexpressed in the pancreatic tumors as compared to the normal organ in a grade-dependent manner and is also significantly associated with poor overall and disease-free survival of PC patients (Fig. 1C–F and Supplementary Fig. S1D). Furthermore, investigating the single-cell expression of STAMBP, we observed predominant expression in tumor cells, with minimal expression detected in immune cells (Fig. 1G). Collectively, these analyses strongly indicated the unfavorable role of STAMBP in PC patients.

The expression levels of STAMBP were examined in 30 paired tissue samples using western blotting and qRT-PCR. The STAMBP protein level was significantly higher in tumor tissues than in adjacent normal tissues (Fig. 1H). Furthermore, STAMBP mRNA expression was higher in PC tissues than in adjacent paracancerous tissues (Fig. 1I). Immunohistochemical findings further supported the above data (Fig. 1J, K). Moreover, we evaluated data from a PC cohort collected retrospectively from the Second Affiliated Hospital of Nanchang University and found that patients with low STAMBP expression had better overall survival and disease-free survival than those with high STAMBP expression (Fig. 1L, M). We further assessed the association between STAMBP expression and clinicopathological factors in 128 patients with PC. The results indicated no significant association of STAMBP expression with age, histological type, or lymph node metastasis; however, it was significantly correlated with tumor size (*P* = 0.036), TNM stage (*P* < 0.001), and differentiation (*P* < 0.001) (Supplementary Table S2). Multivariate Cox regression analysis demonstrated that high STAMBP levels were independent prognostic factors for poor survival in patients with PC (Table S3). Collectively, the findings indicated that STAMBP may serve as a valuable new prognostic factor for human PC.

### Inhibition of STAMBP increased the chemotherapy sensitivity of PC to GEM both in vivo and in vitro

**Fig. 2:**
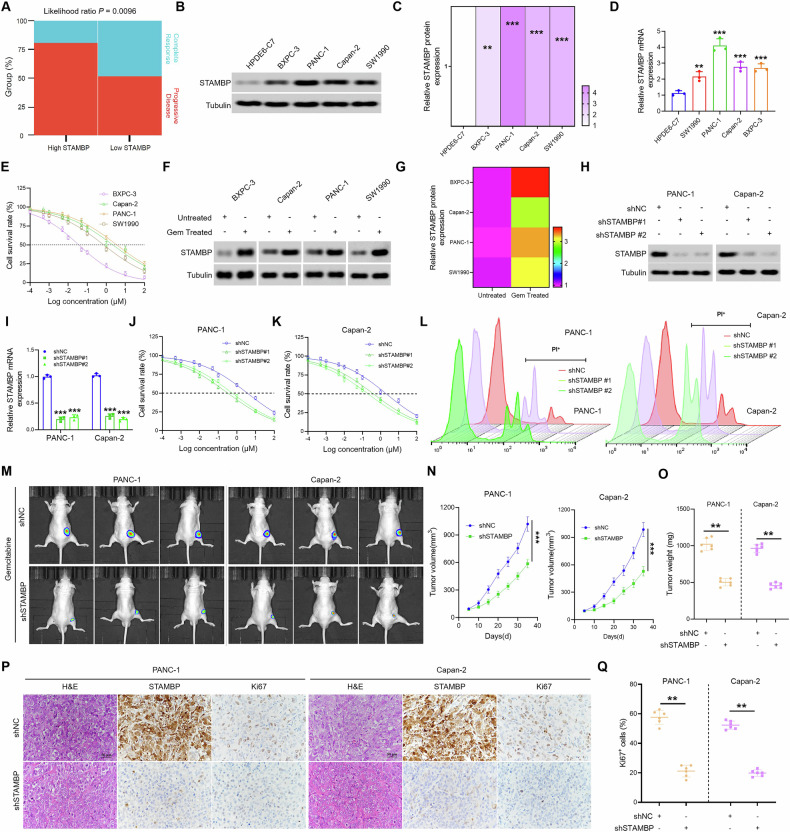
Elevated expression of STAMBP confers gemcitabine resistance in PC. **A** Hierarchy graph demonstrating TCGA analysis on high and low STAMBP-expressing PC patients with progressive disease and complete response after gemcitabine treatment. Median expression values (TPM) of MUC5AC for each group are mentioned in the boxes. Determination (**B**) and quantification (**C**) STAMBP protein levels in four PC cells and the immortalized H6c7 line. Tubulin was used as a loading control.^**^P < 0.01, ^***^P < 0.001. **D** The mRNA levels of STAMBP were detected in four PC cells and the immortalized H6c7 line. ^**^P < 0.01, ^***^P < 0.001. **E** IC50 value of gemcitabine in four PC cell lines by the CCK-8 assay. Determination (**F**) and quantification (**G**) STAMBP protein levels in parental cell lines and chemoresistance cell lines. The protein (**H**) and mRNA levels (**I**) of STAMBP were detected in PANC-1 and Capn-2 cells stably transfected with the STAMBP-silenced vector. Tubulin was used as a loading control. **J**, **K** IC50 value of gemcitabine in PANC-1 and Capn-2 cells transfected with STAMBP-silenced vector by the CCK-8 assay. **L** Results are expressed as scatter diagram for measurement of PI-positive cell population in STAMBP-knockdown PC cells. Images of the tumor tissues (**M**) and the tumor growth curve (**N**) of the STAMBP-silenced group compared to the control group under gemcitabine treatment. ^***^P < 0.001. **O** Comparison of tumor weights of the STAMBP-silenced group compared to the control group, with or without gemcitabine treatment. ^**^P < 0.01. **P** Representative images of H&E, Ki-67, and STAMBP staining of the tumor tissues in the STAMBP-silenced group and the control group with gemcitabine treatment, are displayed. Scale bar, 50 μm. **Q** Quantification of Ki-67 staining in in the STAMBP-silenced group and the control group. ^**^P < 0.01.

In order to comprehend the clinical association of STAMBP with PC chemoresistance, patients who received gemcitabine in the TCGA cohort were segregated into high and low STAMBP-expressors, based on the median values. While low STAMBP-expressors were more responsive to gemcitabine, almost 80.6% of high STAMBP-expressing patients exhibited a progressive disease after gemcitabine treatment (Fig. 2A). Next, we investigated the survival of PC cells that were exposed to various concentrations of GEM and derived the corresponding IC_50_ values. Intriguingly, we discovered that PC cell lines with higher STAMBP expression were more resistant to GEM (Fig. 2B–E), and that the protein and mRNA expression of STAMBP in PC cells increased after GEM treatment (Fig. 2F, G). Next, we transfected the shSTAMBP plasmid into PC cells, and validated the effectiveness of the shSTAMBP plasmid in decreasing STAMBP expression by western blotting and qRT-PCR analyses (Fig. 2H, I). Western blot analysis also confirmed that altering STAMBP does not affect other proteins in the Zn^+^-dependent JAMM deubiquitinases family (Supplementary Fig. S2A, B). Subsequently, the shSTAMBP-PC cells were exposed to various concentrations of GEM. Cell viability assays, we revealed that the suppression of STAMBP expression increased the sensitivity of PC cells (Fig. 2J, K). Conversely, enhanced STAMBP expression decreased the sensitivity of PC cells to GEM (Supplementary Fig. S3A–D). We used flow cytometry to verify the results. The apoptosis experiment suggested that silencing of STAMBP expression can facilitate the apoptotic effect of GEM on PC cells (Fig. 2L). Conversely, STAMBP overexpression attenuated these effects (Supplementary Fig. S3E, F). Next, we injected nude mice with stably knocked-down STAMBP-PC cells and treated them with regular intraperitoneal injections of GEM. After 35 days, compared to the shNC+GEM group, the tumor volume of nude mice in the shSTAMBP+GEM group decreased significantly (Fig. 2M–O). In comparison to the shNC+GEM group, the survival period of nude mice in the shSTAMBP+GEM group was longer (Supplementary Fig. S3G). Next, we measured Ki67 expression in the tumor tissues using immunohistochemistry and found its expression in the tumor tissues of nude mice in the shSTAMBP+GEM group to decrease significantly (Fig. 2P, Q). Based on these results, we confirmed that STAMBP knockdown increased the chemotherapy sensitivity of PC cells to GEM both in vivo and in vitro.

### STAMBP enhanced aerobic glycolysis in PC cells

**Fig. 3:**
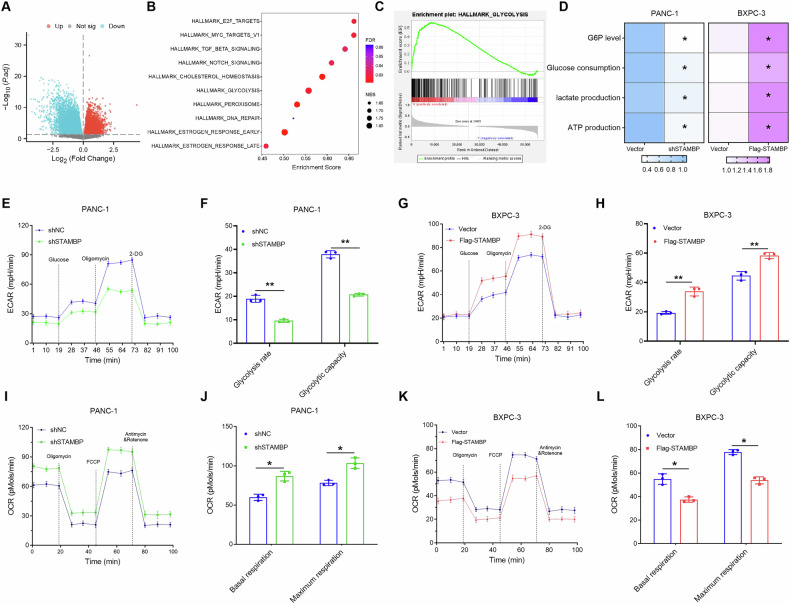
STAMBP overexpression enhances glycolysis in PC cells. **A** Volcano plots showing differentially expressed genes in high and low STAMBP-expressing PC patients from the TCGA-PAAD dataset. |log2FC|>1, *p*-value < 0.05. KEGG pathway analysis (**B**) and GSEA analysis (**C**) of STAMBP -associated differentially expressed genes using the TCGA-PAAD dataset. **D** G6P level, Glucose consumption, lactate production, and ATP levels were detected in PANC-1/shSTAMBP and BxPC-3/Flag-STAMBP cells. Three independent experiments were performed. ^*^P < 0.05. ECAR data showing the glycolytic rate and capacity in PANC-1/shSTAMBP cells (**E** and **F**) and BxPC-3/Flag-STAMBP cells (**G** and **H**). Glucose (10 mM), the oxidative phosphorylation inhibitor oligomycin (1.0 μM), and the glycolytic inhibitor 2-deoxyglucose (2-DG, 50 mM) were sequentially injected into each well at the indicated time points. ^**^P < 0.01. OCR results showing the basal respiration and maximum respiration in PANC-1/shSTAMBP cells (**I** and **J**) and BxPC-3/Flag-STAMBP cells (**K** and **L**). Oligomycin (1.0 μM), the mitochondrial uncoupler carbonyl cyanide p-trifluoromethoxy phenylhydrazone (FCCP, 1.0 μM), and the mitochondrial complex I inhibitor rotenone plus the mitochondrial complex III inhibitor antimycin A (Rote/AA, 0.5 μM) were sequentially injected. ^*^P < 0.05.

To examine the latent mechanism of malignant progression and chemotherapy resistance in PC mediated by STAMBP, we first conducted a GSEA in TCGA database to investigate the possible relationship between a variety of signaling pathways and STAMBP. As illustrated in Fig. 3A–C, the genomes of Hallmark_Glycolysis_Targets were clearly enriched, indicating that the Warburg effect pathway was closely related to high STAMBP levels in PC. Recent studies have revealed that the Warburg effect is closely related to malignant tumor progression and the efficacy of chemotherapeutic drugs. Thus, we hypothesized that STAMBP is likely to be involved in modulating glycolysis in PC, thereby contributing to malignant tumor progression and chemoresistance of PC cells. To test this hypothesis, we examined the role of STAMBP in PC cell glycolysis. Figure 3D shows that the knockdown of STAMBP resulted in a dramatic reduction in the cellular levels of glucose depletion, glucose-6-phosphate (G6P), ATP, and production of lactate in PANC-1 cells, whereas overexpression of STAMBP had the opposite impact in BxPC-3 cells.

In order to validate the impact of STAMBP on PC glycolysis, we determined the extracellular acidification rate (ECAR), which represents the total glycolytic flux. Our findings revealed that ECAR was remarkably diminished in PANC-1 cells after STAMBP knockdown, whereas it was elevated in BxPC-3 cells after STAMBP overexpression (Fig. 3E–H). The cellular OCR, an index of mitochondrial respiration, was also examined. Results indicated that while shSTAMBP/PANC-1 cells exhibited an elevated OCR, STAMBP overexpression lowered the OCR in BxPC-3 cells (Fig. 3I–L). Similar responses were observed in Capan-2/shSTAMBP and SW1990/p-STAMBP cells (Supplementary Fig. S4A–I). Collectively, the findings suggested that STAMBP augmented aerobic glycolysis and suppressed mitochondrial respiration in PC cells.

### PDK1 was identified as the key for STAMBP-enhanced aerobic glycolysis in PC cells

**Fig. 4:**
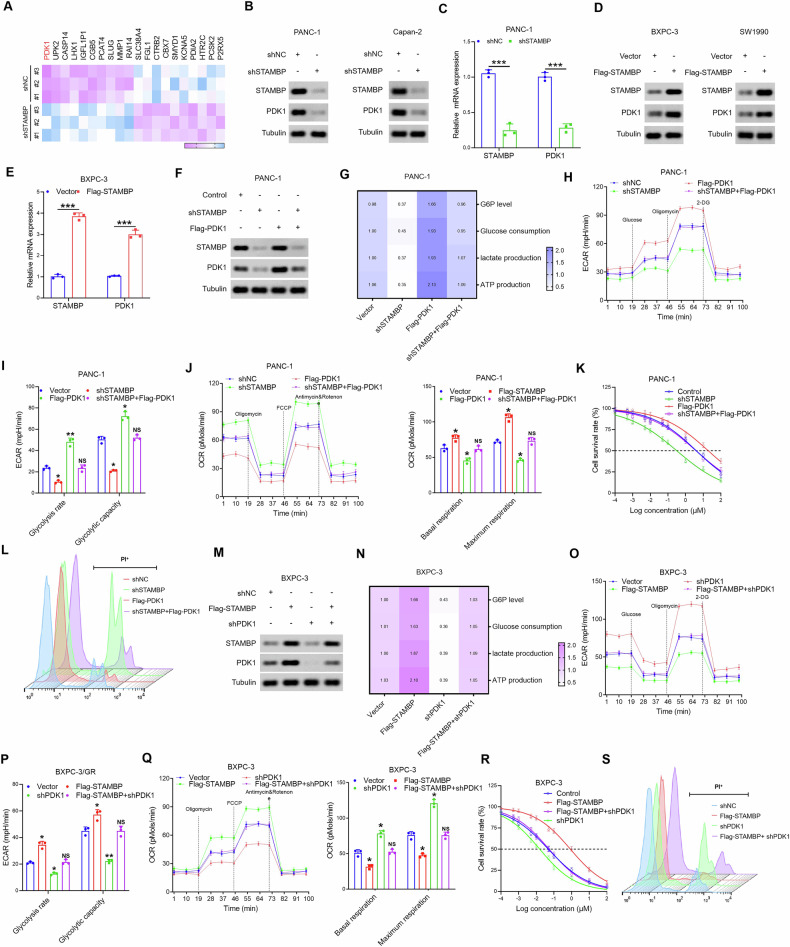
STAMBP promotes glycolysis by upgulating PDK1 expression. **A** Heatmap of genes differentially expressed following STAMBP silence by mass spectroscopic analysis. The protein and mRNA levels of STAMBP and PDK1 were detected in STAMBP knockdown PC cells (**B** and **C**) and STAMBP overexpression PC cells (**D** and **E**). Tubulin was used as a loading control. ^***^P < 0.001. **F** Restoration of PDK1 in STAMBP silenced PANC-1 cells, the protein levels of STAMBP and PDK1 were detected. Tubulin was used as a loading control. **G** G6P levels, glucose uptake, the production of lactate and ATP production were measured in PANC-1/shSTAMBP cells following transfected with PDK1 overexpression vector. **H**, **I** ECAR data showing the glycolytic rate and capacity in PANC-1/shSTAMBP cells following transfected with PDK1 overexpression vector. ^*^P < 0.05, ^**^P < 0.01. **J** OCR results showing the basal respiration and maximum respiration in PANC-1/shSTAMBP cells following transfected with PDK1 overexpression vector. ^*^P < 0.05. **K** IC50 value of gemcitabine in PANC-1/shSTAMBP cells following transfected with PDK1 overexpression vector by the CCK-8 assay. **L** Results are expressed as scatter diagram for measurement of PI-positive cell population in the indicated cells. **M** Knockdown of PDK1 in STAMBP overexpressed BxPC-3 cells, the protein levels of STAMBP and PDK1 were detected. Tubulin was used as a loading control. **N** G6P levels, glucose uptake, the production of lactate and ATP production were measured in BxPC-3/Flag-STAMBP cells following transfected with shPDK1 plasmid. **O**, **P** ECAR data showing the glycolytic rate and capacity in BxPC-3/Flag-STAMBP cells following transfected with shPDK1 plasmid. ^*^P < 0.05. **Q** OCR results showing the basal respiration and maximum respiration in BxPC-3/Flag-STAMBP cells following transfected with shPDK1 plasmid. ^*^P < 0.05. **R** IC50 value of gemcitabine in BxPC-3/Flag-STAMBP cells following transfected with shPDK1 plasmid. by the CCK-8 assay. **S** Results are expressed as scatter diagram for measurement of PI-positive cell population in the indicated cells.

Next, we explored the mechanism by which STAMBP enhanced aerobic glycolysis. RNA-seq data showed that PDK1 expression decreased in shSTAMBP-PANC-1 cells (Fig. 4A). Previous studies had shown that the glycolysis gatekeeper PDK1 is a critical rate-limiting enzyme in aerobic glycolysis. Recent data indicated that PDK1 expression is dysregulated in multiple cancer types. To identify whether STAMBP modulates the expression of PDK1, qRT-PCR and western blotting were performed to assay the expression of PDK1 mRNA and protein in shSTAMBP PC cells. The findings indicated that PDK1 expression was remarkably decreased in PC cells after STAMBP knockdown (Fig. 4B, C). In contrast, STAMBP overexpression significantly increased PDK1 expression in PC cells (Fig. 4D, E). Next, we examined whether PDK1 mediated STAMBP-induced aerobic glycolysis. The knockdown of STAMBP attenuated aerobic glycolysis in PC cells, whereas concomitant PDK1 overexpression decreased the reduction in capacity and glycolytic rate (Fig. 4F–J). Simultaneously, rescue experiments showed that restoration of PDK1 expression abrogated the increase in GEM sensitivity of PC resulting from STAMBP silencing (Fig. 4K, L). In addition, PDK1 knockdown rescued the STAMBP-mediated increase in aerobic glycolysis in PC cells (Fig. 4M–Q). The reduction in PDK1 expression remarkably decreased GEM resistance in PC cells, which was enhanced by STAMBP (Fig. 4R, S). These findings demonstrated that STAMBP promoted chemoresistance in PC by increasing PDK1-mediated aerobic glycolysis.

### E2F1 was identified as a transcription factor of PDK1 in PC cells

**Fig. 5:**
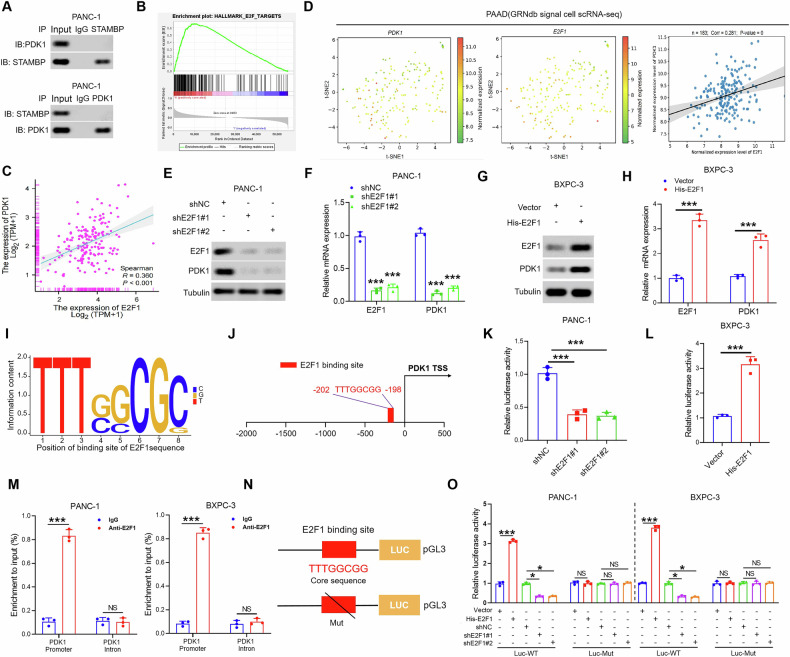
STAMBP upregulates PDK1 by stabilization E2F1 in PC cells. **A** Co-IP assay showing that endogenous STAMBP and PDK1 were not directly bound in PANC-1 cell. **B** GSEA comparing the gene sets of E2F1 targets in STAMBP^high^ and STAMBP^low^ PC patients. Data were obtained from the TCGA-PAAD database. **C** The correlation between the expression of E2F1 and PDK1 in PC patients from the TCGA-PAAD dataset. **D** The correlation between the expression of E2F1 and PDK1 gene in pancreatic cancer using the single-cell sequencing data set from the TISCH database. The protein and mRNA level of E2F1 and PDK1 were detected in PANC-1/shE2F1 cells (**E** and **F**) and BxPC-3/His-E2F1 cells (**G** and **H**). ^***^P < 0.001. **I** Map of E2F1 binding site sequence. **J** Schematic illustration of the potential E2F1 binding site in the PDK1 promoter. Full-length PDK1 promoter luciferase construct was transfected into the PANC-1/shE2F1 cells (**K**) and BxPC-3/His-E2F1 (**L**) cells. Transcriptional activation was analyzed with the dual luciferase reporter assay. ^***^P < 0.001 vs control group. **M** ChIP-qPCR assay demonstrated the direct binding of E2F1 to the PDK1 promoter in PANC-1 and BxPC-3 cells. Gene enrichment was quantified relative to input controls by qPCR using primers specific for the promoter regions of PDK1. Results are shown as a fold change of qPCR value over IgG, with intron of the human PDK1 gene used as a negative control. **N** The 8-base pair sequence of the E2F1 consensus site and deletion mutation analysis identified a responsive transcription factor E2F1 binding site in the PDK1 promoter. **O** Luciferase constructs for wild-type, or mutant PDK1 promoter was transfected into PANC-1/shE2F1 cells and BxPC-3/His-E2F1 cells. Results were normalized to Renilla luciferase activity and are expressed as fold change in luciferase activity relative to the control. ^*^P < 0.05, ^***^P < 0.001.

STAMBP has been reported to interact with various substrates in order to play its role [20, 24]. To further elucidate the mechanism by which STAMBP modulates STAMBP expression in PC cells, we investigated whether there are direct interactions between STAMBP and PDK1. However, co-immunoprecipitation (Co-IP) revealed no direct interaction between STAMBP and PDK1 (Fig. 5A and Supplementary Fig. S5A). E2F1 has been reported to regulate PDK1 expression. Simultaneously, through bioinformatic analysis, E2F1 was found to be a potentially modified substrate protein of STAMBP (Supplementary Fig. S5B). As illustrated in Fig. 5B, the genomes of Hallmark_E2F_Targets were clearly enriched, indicating that the E2F pathway was closely related to high STAMBP levels in PC. Consequently, we hypothesized that STAMBP modulates PDK1 expression via E2F1. To verify this hypothesis, we first analyzed the expression correlation between E2F1 and PDK1 using RNA-seq data derived from TCGA-PAAD projects. The resulting expression correlation scatter plot showed a signifcant positive correlation between E2F1 and PDK1 (Fig. 5C). Additionally, a single-cell RNA sequencing analysis using GRNdb database confrmed that E2F1 expression was signifcantly correlated with PDK1 expression at the transcriptional level (Fig. 5D). Furthermore, we examined whether E2F1 modulates PDK1 in PC cells and tested the alterations in the expression of PDK1 in PANC-1 cells with E2F1 knockdown. The findings demonstrated that the mRNA and protein expression of PDK1 in PANC-1 cells was reduced after E2F1 knockdown (Fig. 5E, F and Supplementary Fig. S5C, D), whereas upregulation of E2F1 showed the opposite result in PC cells (Fig. 5G, H and Supplementary Fig. S5E, F). Next, to gain an understanding of potential transcription factor (TF) binding sites within PDK1 promoter region, we conducted JASPAR analysis, revealing the presence of E2F1 binding motifs within the PDK1 promoter region in PAAD cell lines (Fig. 5I). Notably, the site located at -202 to -198 (TTTGGCGG) upstream of the EFTUD2 transcription start site was the top-ranked predicted binding sites (Fig. 5J). In addition, a dual-luciferase reporter gene assay was performed; its result displayed that the decrease and overexpression of E2F1 can suppress and increase PDK1 luciferase activity, respectively (Fig. 5K, L and Supplementary Fig. S5G, H). ChIP-qPCR was performed using an antibody specific to E2F1. The E2F1-bound complex displayed remarkable enrichment of the PDK1 promoter compared to the IgG-bound samples (Fig. 5M). Further, functional validation was performed through a dual luciferase reporter assay. The assay showed an increase in luciferase activity when the PDK1 promoter was introduced along with E2F1. Conversely, down-regulation of E2F1 significantly attenuated the luciferase activity in both PANC-1 and BXPC-3 cell lines. Importantly, this effect was abolished when the binding site within the PDK1 promoter was mutated (Fig. 5N, O). Collectively, the data further supported the notion that E2F1 is a transcription factor of PDK1 in PC cells.

### STAMBP regulated E2F1 to promote the PDK1-mediated Warburg effect

**Fig. 6:**
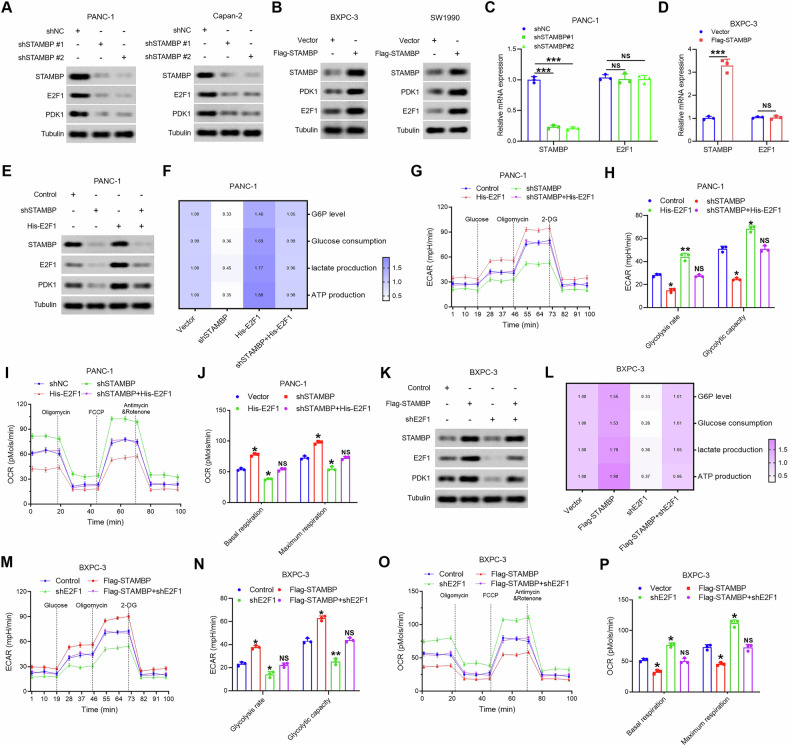
Oncogenic effect of STAMBP is dependent on E2F1 enhancement. Western blotting showing the protein expression of STAMBP, E2F1, and PDK1 in STAMBP knockdown PC cells (**A**) and STAMBP overexpression PC cells (**B**). Tubulin was a loading control. **C** and **D**, qRT-PCR analysis of STAMBP and E2F1 at the mRNA level in STAMBP knockdown PC cells (**C**) and STAMBP overexpression PC cells (**D**). ^***^P < 0.001. **E** Restoration of E2F1 in STAMBP silenced PANC-1 cells, the protein levels of STAMBP, E2F1, and PDK1 were detected. Tubulin was used as a loading control. **F** G6P levels, glucose uptake, the production of lactate and ATP production were measured in PANC-1/shSTAMBP cells following transfected with E2F1 overexpression vector. **G**, **H** ECAR data showing the glycolytic rate and capacity in PANC-1/shSTAMBP cells following transfected with E2F1 overexpression vector. ^*^P < 0.05, ^**^P < 0.01. **I**, **J** OCR results showing the basal respiration and maximum respiration in PANC-1/shSTAMBP cells following transfected with E2F1 overexpression vector. ^*^P < 0.05. **K** Knockdown of E2F1 in STAMBP overexpressed BxPC-3 cells, the protein levels of STAMBP E2F1, and PDK1 were detected. Tubulin was used as a loading control. **L** G6P levels, glucose uptake, the production of lactate and ATP production were measured in BxPC-3/Flag-STAMBP cells following transfected with shE2F1 plasmid. **M**, **N** ECAR data showing the glycolytic rate and capacity in BxPC-3/Flag-STAMBP cells following transfected with shE2F1 plasmid. ^*^P < 0.05. **O**, **P** OCR results showing the basal respiration and maximum respiration in BxPC-3/Flag-STAMBP cells following transfected with shE2F1 plasmid. ^*^P < 0.05.

We examined whether STAMBP regulates the expression of PDK1 through E2F1. To achieve this, we investigated the variations in expression of PDK1 and E2F1 in PANC-1 cells following STAMBP knockdown. The results indicated that both PDK1 and E2F1 expression was remarkably reduced in PANC-1 and Capan-2 cells after STAMBP knockdown (Fig. 6A). Conversely, STAMBP overexpression increased the expression of PDK1 and E2F1 in PC cells (Fig. 6B). However, E2F1 mRNA levels did not influence the variation in STAMBP expression in PC cells (Fig. 6C, D and Supplementary Fig. S6A, B). The results suggested that E2F1 was involved in the modulation of PDK1 expression mediated by STAMBP. To further prove that STAMBP modulated the expression of PDK1 via E2F1 in PC cells, rescue experiments demonstrated that E2F1 overexpression restoration abrogated the decrease in PDK1 resulting from STAMBP silencing (Fig. 6E). Simultaneously, STAMBP knockdown attenuated aerobic glycolysis in PC cells, whereas concomitant E2F1 overexpression decreased the reduction in capacity and glycolytic rate (Fig. 6F–J). Furthermore, the reduction of E2F1 remarkably decreased PDK1 expression in PC cells, which was enhanced by STAMBP (Fig. 6K). E2F1 knockdown rescued the STAMBP-mediated increase in aerobic glycolysis in PC cells (Fig. 6L–P). Therefore, E2F1 is the key to STAMBP-related promotion of PDK1-mediated aerobic glycolysis in PC cells.

### STAMBP stabilized E2F1 expression by inhibiting its ubiquitination degradation

**Fig. 7:**
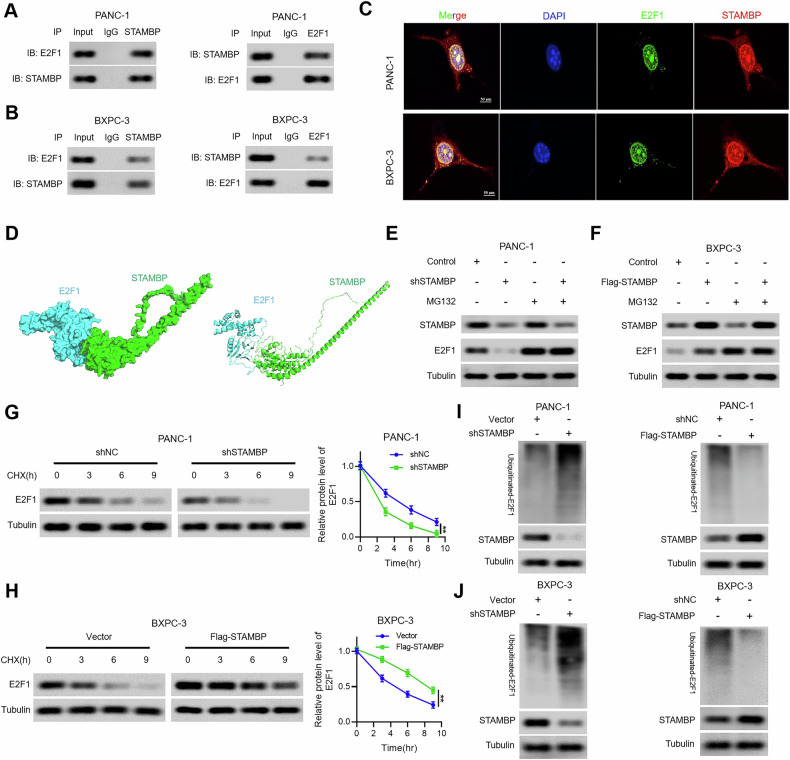
STAMBP interacting with E2F1 and stabilizing E2F1 expression via deubiquitination. **A**, **B** The interaction between STAMBP and E2F1 was confirmed by co-IP assay in PANC-1 cells and BxPC-3 cells. **C** Co-localization studies of PANC-1 and BxPC-3 cells using anti-STAMBP antibody (1:100, red) and anti-E2F1 antibody (1:100, green), followed by DAPI nuclear counterstaining (blue). Scale bar, 50 μm. **D** Docking conformation of the first ranking score. Three-dimensional structure of STAMBP and E2F1 in ribbon (left) and surface (right) format. STAMBP is shown in blue. E2F1 is shown in green. PC cells transduced with shSTAMBP (**E**) or Flag-STAMBP (**F**) were treated with 10 μM MG132. Cells were collected at 6 h and immunoblotted with the antibodies indicated. Representative (right) and quantitative (left) results of E2F1 protein level in PANC-1/shSTAMBP cells (**G**) or BxPC-3/Flag-STAMBP cells (**H**). The cells were treated with cycloheximide (CHX, 100 mg/ml) for indicated time points were subjected to western blot analysis. ^**^P < 0.01. Lysates from PC cells transduced with shSTAMBP (**I**) or Flag-STAMBP (**J**) were immunoprecipitated with the anti-Ub and immunoblotted with the anti-E2F1. Cells were treated with MG132 for 6 h before collection.

We next investigated the mechanism underlying STAMBP modulation of E2F1 expression in PC. Our data indicated that there was no apparent difference in E2F1 mRNA levels after altering STAMBP expression in PC cells, hence demonstrating the positive modulation of E2F1 by STAMBP at the post-transcriptional level. Furthermore, E2F1 was suggested to be degraded by the ubiquitin-protease system. Since STAMBP is a deubiquitinase, we hypothesized that it might modulate the degradation and ubiquitination of E2F1 in PC. To test this hypothesis, we first investigated whether STAMBP interacts with E2F1 in PC cells. Co-IP assays utilizing endogenous E2F1 and STAMBP antibodies in BxPC-3 and SW1990 cells verified the interaction between E2F1 and STAMBP (Fig. 7A, B). Confocal microscopy experiments confirmed the co-localization of E2F1 and STAMBP in PC cells (Fig. 7C). Additionally, Docking analysis indicated a binding interaction between E2F1 and STAMBP (Fig. 7D). Furthermore, we found that E2F1 protein levels were restored in PC cells with knockdown and overexpression of STAMBP after treatment with the proteasome inhibitor MG132 (Fig. 7E, F). Subsequently, we evaluated E2F1 protein degradation in STAMBP-knockdown cells after exposure to cycloheximide. As shown in Fig. 7G, H, the data suggested that STAMBP silencing contributed remarkably to E2F1 protein degradation in PC cells. Finally, the ectopic expression of STAMBP diminished the level of E2F1 ubiquitination, whereas STAMBP knockdown elevated E2F1 polyubiquitination (Fig. 7I, J). These findings indicated that STAMBP, a deubiquitinase, is responsible for stabilizing E2F1 in PC cells via the ubiquitin-proteasome pathway.

### Entrectinib increased GEM sensitivity in PC by inhibiting the activity of STAMBP

**Fig. 8:**
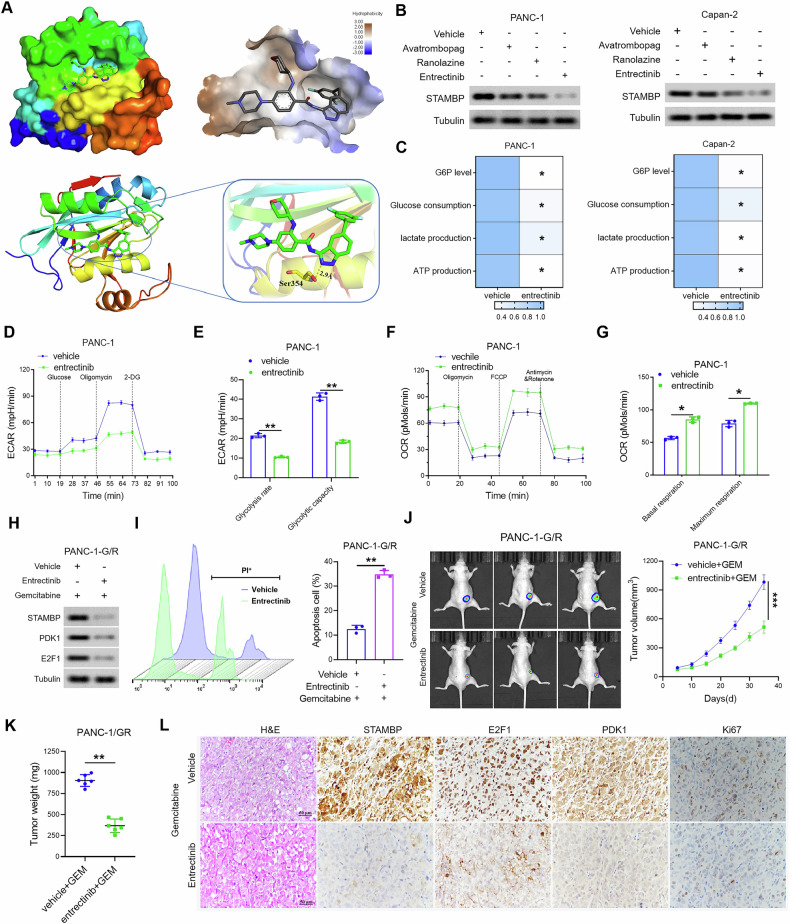
Identification and characterization of entrectinib as a STAMBP inhibitor that enhances gemcitabine efficiency. **A** Docking analysis for binding between STAMBP and the drugs entrectinib from the Food and Drug Administration drug library. **B** The protein levels of STAMBP in PC cells were detected after treatment with the entrectinib, ranolazine, and avatrombopag, respectively. Tubulin was used as a loading control. **C** G6P levels, glucose uptake, the production of lactate and ATP production were measured in PC cells following treatment with the entrectinib. ^*^P < 0.05. **D**, **E** ECAR data showing the glycolytic rate and capacity in PANC-1 cells following treatment with the entrectinib. ^**^P < 0.01. **F**, **G** OCR results showing the basal respiration and maximum respiration in PANC-1 cells following treatment with the entrectinib. ^*^P < 0.05. **H** The protein levels of STAMBP, PDK1, and E2F1 in PANC-1-G/R cells were detected after treatment with the entrectinib. **I** Representative images (left) and quantification (right) of PI-positive cell population in the indicated cells. ^**^P < 0.01. **J** Images of the tumor tissues and the tumor growth in nude mice treated with entrectinib, gemcitabine, or both. ^***^P < 0.001. **K** Tumor wight in nude mice treated with entrectinib, gemcitabine, or both. ^**^P < 0.01. **L** Representative images of H&E, STAMBP, E2F1, PDK1, and Ki-67 staining of the tumor tissues in nude mice treated with entrectinib, gemcitabine, or both. Scale bar, 50 μm.

To identify the drugs that can target and inhibit the activity of STAMBP, we performed protein structure and drug screening through the Food and Drug Administration drug library. We found that entrectinib, ranolazine, and avatrombopag to act on the active pocket of STAMBP (Fig. 8A and Supplementary Table S4). As shown in Fig. 8B, the data indicate that entrectinib could inhibit STAMBP expression in PANC-1 and Capan-2 cells. Further, treatment with entrectinib resulted in a dramatic reduction in the cellular levels of glucose depletion, glucose-6-phosphate (G6P), ATP, and production of lactate in PANC-1 and Capan-2 cells (Fig. 8C). The results indicate that ECAR was remarkably diminished and OCR was elevated in PANC-1 and Capan-2 cells after treatment with entrectinib (Fig. 8D–G and Supplementary Fig. S7A–D). To confirm the result, we also treated drug-resistant PC cells with entrectinib. After treatment with entrectinib, the expression of STAMBP, E2F1, and PDK1 was decreased in drug-resistant PC cells (Fig. 8H and Fig. 6E), and PC cells exhibited a remarkable increase in sensitivity to GEM (Fig. 8I and Supplementary Fig. S7F, G). To further determine the clinical significance of entrectinib in mitigating GEM resistance in PC, the effects of GEM treatment alone or in combination with entrectinib were examined in subcutaneous tumor-bearing nude mice. Figure 8J, K shows that the combination of GEM and entrectinib decreased tumor weight and volume of mice. IHC analysis revealed that STAMBP, E2F1, PDK1 and Ki-67 expression in tumors treated with the combination was lower than that in tumors treated with GEM alone (Fig. 8L). In the PDX model of PC, entrectinib remarkably improved the sensitivity of PC cells to GEM compared to that in the control group (Supplementary Fig. S7H). Immunohistochemistry confirmed that entrectinib treatment reduced the expression of STAMBP, E2F1, PDK1 and Ki-67 expression in tumor tissues (Supplementary Fig. S7I). Based on the above results, we confirmed that entrectinib increased GEM sensitivity in PC by inhibiting the activity of STAMBP.

### Correlation among the expression of STAMBP, E2F1, and PDK1 in PC

**Fig. 9:**
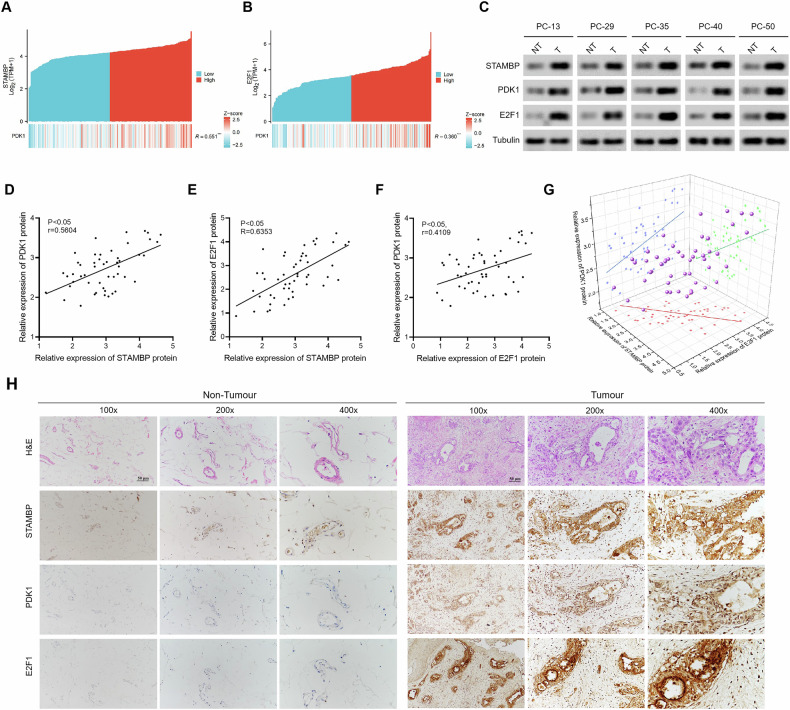
Correlation among the expression of STAMBP, E2F1, and PDK1 in PC tissues. **A** Spearman correlation analysis of STAMBP and PDK1 mRNA expression in PC tissues. **B** Spearman correlation analysis of E2F1 and PDK1 mRNA expression in tumor tissues. **C** Representative western blotting analysis of STAMBP, E2F1, and PDK1 protein expression in tumor or nontumorous tissues. Tubulin was used as a loading control. (***P < 0.001). **D** Spearman correlation analysis of STAMBP and PDK1 protein expression in PC tissues. **E** Spearman correlation analysis of STAMBP and E2F1 protein expression in PC tissues. **F** Spearman correlation analysis of PDK1 and E2F1 protein expression in PC tissues. **G** Correlation among the expression of STAMBP, E2F1, and PDK1 in PC tissues. **H** Representative immunohistochemistry staining of STAMBP, E2F1, and PDK1 in PC tissues. Scale bar, 50 μm.

In addition, a co-expression heat map derived from the TCGA database showed a significantly positive correlation between the expressions of STAMBP and PDK1 (Fig. 9A). And TCGA database also showed a significantly positive correlation between the expressions of E2F1 and PDK1 (Fig. 9B). Considering the importance of STAMBP in the malignant progression of PC and in the sensitivity to GEM treatment, we detected the expression of STAMBP, E2F1, and PDK1 through western blotting and analyzed their correlation in 60 fresh PC tissues. Western blot results showed that the expression of STAMBP, E2F1, and PDK1 was higher in PC tissues than in the corresponding adjacent tissues (Fig. 9C). Scatter plot analysis revealed a positive correlation among the protein expression levels of STAMBP, E2F1, and PDK1 in PC tissues (Fig. 9D–G). IHC staining revealed a positive correlation between the expression levels of STAMBP and E2F1 in PC samples. Moreover, PDK1 staining corresponded to STAMBP and E2F1 staining (Fig. 9H), suggesting that STAMBP promotes aerobic glycolysis in PC cells. The results provided clearly suggested that targeting STAMBP to inhibit E2F1-PDK1 axis-mediated glycolysis may be a new strategy for overcoming malignant progression and GEM resistance in PC.

## Discussion

Due to its rapid progression, PC remains the second leading cause of death worldwide [25]. The number of new cases is expected to increase by more than twice in the next few years. Chemotherapy is extensively used to prevent or slow the progression of PC [26–28]. However, it is not administered to all patients with PC. In many cases, chemotherapy resistance occurs after a successful initial treatment period [29–31]. Thus, better understanding of the mechanisms of chemoresistance in PC cells and development of new targets to enhance the chemosensitivity of PC cells are considered essential.

STAMBP is a ubiquitinase belonging to the JAMM family that plays a critical role in the sequencing and endocytosis of cell surface receptors [32, 33]. Growing evidence shows that STAMBP modulates substrate stability through the cleavage of polyubiquitination from the substrate [32]. STAMBP overexpression promotes the malignant behavior of cancer cells in multiple cancers, such as breast cancer, triple-negative breast cancer, PC, and lung cancer. For example, STAMBP potentiates the metastatic potential of melanoma cells by modulating SLUG stability [34]. Xu et al. demonstrated that STAMBP facilitates metastasis in lung adenocarcinoma by activating the EGFR/MAPK signaling pathway [22]. However, detailed information on the role and molecular mechanisms of STAMBP in PC is unavailable. In the present study, we reported that STAMBP is highly expressed in PC tissues; patients with low STAMBP expression showed better overall survival and disease-free survival than those with high STAMBP expression. High STAMBP levels are an independent prognostic factor for poor survival in patients with PC. STAMBP knockdown remarkably elevated GEM sensitivity in PC both in vitro and in vivo. As a result, our findings are of great significance for a better understanding of the role of STAMBP in the chemotherapy resistance of PC and for evaluating the possibility of STAMBP as a therapeutic target.

Metabolic alterations and reprogramming of tumor cells help them survive and grow in harsh microenvironments, which can lead to resistance to chemotherapy [35, 36]. Consequently, a better understanding of the mechanism underlying the regulation of aerobic glycolysis in PC may help reverse the chemoresistance of PC. As a key glycolytic enzyme, PDK1 plays a major role in chemoresistance in several malignancies [37–39]. For instance, Liu et al. reported that circ-LPAR3 might contribute to cisplatin resistance in ovarian cancer through the miR-634/PDK1 axis [40]. Zhang et al. demonstrated that ELK1 accelerates aerobic glycolysis via PDK1 to augment chemotherapy resistance in osteosarcoma [41]. Therefore, identifying and controlling the mechanism of PDK1-mediated Warburg effect would be crucial for overcoming the challenges associated with chemotherapy resistance in PC. In this study, we revealed a novel mechanism of inhibition of chemotherapy resistance and aerobic glycolysis in PC, which occurs through the STAMBP silencing-mediated reduction of PDK1 expression. First, we confirmed that STAMBP inhibits oxidative phosphorylation while promoting aerobic glycolysis in PC cells but promotes chemotherapy resistance by enhancing the Warburg effect in PC cells. Furthermore, our data demonstrated that STAMBP elevates PDK1 expression, thereby augmenting aerobic glycolysis in PC cells. Moreover, the restoration of PDK1 expression abrogated the increase in GEM sensitivity resulting from STAMBP silencing in PC. Meanwhile, STAMBP knockdown attenuated aerobic glycolysis in PC cells, whereas concomitant PDK1 overexpression decreased the reduction in capacity and glycolytic rate. Importantly, irinotecan hydrochloride and the disaccharide lapatinib, targeting STAMBP, remarkably enhanced the chemotherapy sensitivity of PC cells. These findings suggested that STAMBP contributes to PC by increasing PDK1-mediated aerobic glycolysis.

Next, we explored the molecular mechanisms by which STAMBP modulates PDK1 expression. STAMBP regulates substrate stability by removing substrate polyubiquitination [20, 32, 42]. It interacts with different substrates to play its role [20, 32, 42]. Research shows that the transcription factor E2F1 plays a critical role in modulating malignant progression and chemoresistance in cancer [43–45]. A new mechanism was revealed here, in which STAMBP modulates the expression of PDK1 by influencing the expression of E2F1. Our findings revealed that both protein and mRNA expression of PDK1 was remarkably reduced after E2F1 knockdown. E2F1 is a transcription factor of PDK1 in PC cells. Restoration of E2F1 expression abrogated the increase in GEM sensitivity resulting from STAMBP silencing in PC. Simultaneously, STAMBP knockdown attenuated aerobic glycolysis in PC cells, whereas concomitant E2F1 overexpression decreased the reduction in capacity and glycolytic rate. Taken together, our data indicated that STAMBP promotes the PDK1-mediated Warburg effect and chemotherapy resistance through an E2F1-dependent mechanism.

Next, we examined the mechanism by which STAMBP modulates E2F1 expression. Tian et al. showed that STAMBP might promote the stabilization of ULK1 by removing its K48-linked ubiquitin chains, thereby participating in the regulation of autophagic flux [46]. Bednash et al. demonstrated that STAMBP is necessary to limit K63-linked ubiquitination of NLRP3 [42]. Further, the study reported that E2F1 is a critical transcription factor [47]. E2F1 degradation, mediated by the ubiquitin-proteasome pathway, is a major mechanism for modulating intracellular E2F1 levels [48]. Consistent with these results, our findings indicated, for the first time, that STAMBP, a deubiquitinase, is responsible for stabilizing E2F1 in PC through the ubiquitin-proteasome pathway. This finding was supported by the following observations: first, STAMBP directly bound to E2F1 in PC cells. Second, STAMBP silencing contributed to E2F1 protein degradation in PC cells. Third, STAMBP repressed E2F1 K48-linked polyubiquitination, thus stabilizing the expression of E2F1 in PC cells. Finally, we found the chemoresistance and aerobic glycolytic activity of STAMBP in PC to be dependent on the stabilization of E2F1.

Importantly, this study comprehensively analyzed the effects of the FDA drug entrectinib on PC and suggested it for further evaluation in clinical trials as a first- or second-line therapy in combination with GEM. On August 15, 2019 the Food and Drug Administration granted accelerated approval to entrectinib for adults and pediatric patients, 12 years of age and older, with solid tumors that have a neurotrophic tyrosine receptor kinase (NTRK) gene fusion without a known acquired resistance mutation, are metastatic, in which surgical resection is likely to result in severe morbidity, have progressed following treatment, or have no satisfactory standard therapy [49, 50]. The FDA further approved entrectinib for adults with metastatic non-small cell lung cancer (NSCLC) whose tumors are ROS1-positive [51]. In this study, we found that entrectinib binds to the active pocket of STAMBP. STAMBP, E2F1, and PDK1 were downregulated in the combination treatment group than in the GEM-treated group. In the PDX model of PC, compared to the control group, entrectinib improved the sensitivity of PC cells to GEM remarkably, and glycolysis in tumor tissues was also significantly inhibited. Previous studies have reported that inhibiting tyrosine kinase may increase the sensitivity of pancreatic cancer to gemcitabine [52, 53]. However, it remains unclear whether entrectinib can simultaneously inhibit both tyrosine kinase and the STAMBP protein to further enhance the sensitivity of pancreatic cancer to gemcitabine, which warrants further exploration in the future. In general, targeting STAMBP with this combination could potentially be a novel therapeutic strategy for improving treatment outcomes in PC.

## Conclusion

**Fig. 10:**
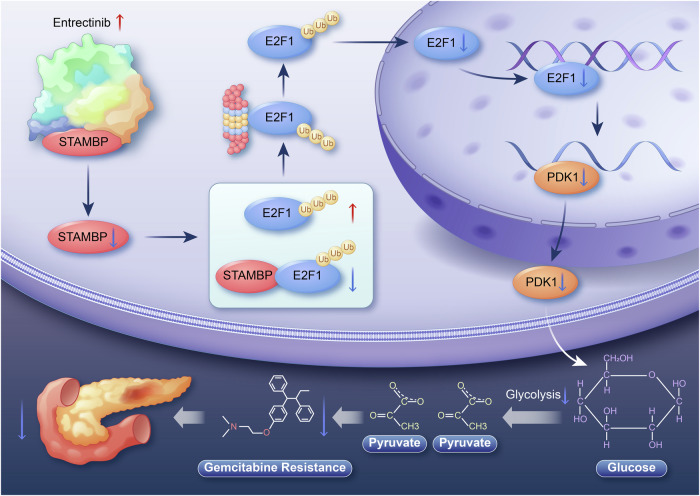
Proposed mechanistic scheme of STAMBP confers gemcitabine resistance by in promoting the E2F1/PDK1 axis-mediated aerobic glycolysis in pancreatic cancer.

Overall, we presented the first evidence that STAMBP expression is increased in PC-resistant tissues and is linked to the prognosis of patients with PC. We further showed that STAMBP leads to chemotherapy resistance in PC by increasing PDK1-mediated aerobic glycolysis. Our findings additionally demonstrated that STAMBP promotes the PDK1-mediated Warburg effect and chemotherapy resistance by modulating E2F1, which is achieved by binding directly to E2F1 and suppressing its degradation and ubiquitination (Fig. 10). Importantly, entrectinib-mediated targeting of STAMBP enhanced the chemosensitivity of PC cells remarkably. Based on these findings, STAMBP was concluded to act against chemoresistance in PC by enhancing aerobic glycolysis mediated by E2F1/PDK1. Therefore, targeting the STAMBP/E2F1/PDK1 axis may be a promising therapeutic strategy for PC.

## Materials and methods

### Cell lines and cell culture

PC cell lines, including CFPAC-1, BxPC-3, AsPC-1, SW1990, and MIA PaCa2, and the immortalized human pancreatic ductal epithelial cell line hpDE6-C7 were acquired from the Type Culture Collection, Chinese Academy of Sciences (Shanghai, China). The cell lines were cultivated in Dulbecco’s Modified Eagle Medium (DMEM)/F-12 medium containing 10% FBS (Gibco), streptomycin (0.1 mg/mL), and penicillin (100 U/mL) (Invitrogen, USA) at 37 °C and 5% CO_2_. GEM-resistant AsPC-1/GR, BXPC-3/GR, SW1990/GR and PANC-1/GR were generated by exposing GEM-sensitive AsPC-1, BXPC-3, SW1990 and PANC-1 parental cells to an initial concentration (1/5 of IC_50_) of GEM (Selleck) for one week. When the cells returned to normal growth after the recovery period, the GEM concentration was progressively increased from the initial concentration to the final (RI) > 5 (RI = IC_50, drug-resistant cell_ /IC_50, parental cell_) over a 10-month period.

### Clinical PC specimens

A total of 128 human PC samples, which were obtained from patients with a clinical and histopathological PC diagnosis, were collected from the Second Affiliated Hospital of Nanchang University. Informed consent was obtained from each patient and the study protocol was approved by the ethics committee of the second affiliated hospital of Nanchang University([2019] No. (053)).

### RNA extraction and qRT-PCR

Following the manufacturer’s instructions, total RNA was separated from PC tissues or cells using TRIzol reagent (Invitrogen, USA). After the extraction, RNA concentration was calculated and the purified RNA was stored in a −80 °C freezer to prevent degradation. Applied Biosystems®7900HT rapid real-time PCR system (Thermo Fisher Scientific, USA) and TB Green qPCR Master Mix (Takara, Japan) were employed for qRT-PCR. GAPDH acted as the internal control, and the 2^-ΔΔCT^ approach was applied for calculating the relative expression of mRNAs.

### Immunoprecipitation and western blotting

Total protein was extracted using cell lysis buffer for IP and protease inhibitor cocktails for western blot. For the co-IP assays, cell lysates were pre-treated with protein A/G magnetic beads (Santa Cruz, USA) by incubating them with IgG or the specified primary antibody at 4 °C overnight and subsequently at 4 °C for 120 min. Equal amounts of immunoprecipitates or cell lysates were isolated by SDS-PAGE and transferred to polyvinylidene difluoride (PVDF) membranes (Millipore, USA), which were then blocked with 5% skim milk and blotted overnight using primary antibodies. After incubation with the appropriate anti-rabbit/mouse (Zsbio, China) secondary antibody conjugated to horseradish peroxidase (HRP), the protein signal was observed by chemiluminescence (GE, USA) following the manufacturer’s instructions. The primary antibodies included anti-E2F1 (1:1000, Proteintech, 66515-1-Ig), anti-PDK1 (1:1000, Abcam, ab110025), anti-STAMBP (1:1000, Proteintech, 11346-1-AP), anti-HA (1:1000, Sigma, SAB1306082), anti-Flag (1:1000, Sigma, F1804), anti-Ki67 (1:1000, Santa Cruz, sc-23900), anti-GAPDH (1:1000, Abcam, ab8245), and anti-ubiquitin (1:1000, Santa Cruz, sc-53509).

### Immunohistochemistry (IHC) and immunofluorescence (IF)

Subcutaneous tumor and human tissue samples were fixed in formalin, embedded in paraffin, sectioned, and de-paraffinized. The sections were subsequently blocked for half an hour using a serum-free protein blocking buffer (DAKO, USA) and treated with the following primary antibodies: anti-E2F1 (1:200, Proteintech, 66515-1-Ig), anti-STAMBP (1:200, Proteintech, 11346-1-AP), anti-Ki67 (1:200, Santa Cruz, sc-23900) and anti-PDK1 (1:200, Abcam, ab110025). The secondary antibody conjugated with anti-rabbit horseradish peroxidase (HRP) was next incubated for 30 min at ambient temperature. Slides were counterstained with bluing reagent and hematoxylin. For IF, the cells were fixed on ice with 4% paraformaldehyde for 15 min after the indicated treatment, washed thrice with cold PBS, and finally permeabilized with 0.1% TritonX-100 for 5 min. After blocking in 1.5% BSA for 60 min, the cells were inoculated overnight with the primary antibody designated anti-STAMBP (1:100, Proteintech, 11346-1-AP) and anti-E2F1 (1:100, Proteintech, 66515-1-Ig at 4 °C. The nuclei were then counterstained with DAPI after three washes with cold 0.1% Tween20-PBS and incubated with fluorescent-dye conjugated secondary antibodies for 2 h. The cells were subsequently visualized using a laser-scanning confocal microscope (Olympus, Japan).

### Molecular docking analysis

The structures of compounds and proteins were optimized to perform docking analysis using the Discovery Studio (DS) 2019 Client. The force field and hydrogenation structures were optimized by removing water molecules from the model structures of E2F1 and STAMBP receptors. Protein ligands and receptors were docked in an induced-fit approach using the ZDOCK module of the DS software. A 20 Å cavity was chosen as a docking-active region with standard parameters for the docking calculations. The induced-fit approach allowed GEM to bind to the protein receptor by employing the best conformation, which, in turn, altered the original conformation to bind to GEM better. The highest-scoring docking model was then examined and validated.

### Quantitative flow cytometry

According to the manufacturer’s instructions, a PE Annexin V Apoptosis Detection Kit I (BD Biosciences) was used to assess apoptosis. FACSCalibur flow cytometry (BD Biosciences, USA) was used to analyze the apoptotic cells.

### 5-Ethynyl-20-deoxyuridine (EdU) incorporation assay

EdU Cell Proliferation Kit containing Alexa Fluor 555 was provided by RiboBio (Guangzhou, China). PC cells were fixed with 4% paraformaldehyde after the specified treatment, incubated in EdU solution (50 mM) for 2 h, and subsequently stained using DAPI; the Edu-labeled cells were photographed by a fluorescence microscope (Olympus, Japan).

### Luciferase reporter assay

Luciferase reporter plasmids with PDK1 or empty pGL3-promoter were transfected into PC cells together with pRL-TK, along with the control vector, E2F1, or mutated or truncated E2F1. After 2 days, luciferase activity was measured using a Hidex Sense instrument (Hidex, Finland) and a Dual Luciferase Reporter Gene Assay Kit (Beyotime, RG027, Shanghai, China). Firefly luciferase readings were normalized to the co-transformed Renilla luciferase control.

### Plasmid construction and transfection

Plasmids encoding shRNAs targeting PDK1, E2F1, or STAMBP were generated by GenePharma (Shanghai, China). The silencing of STAMBP was mediated by RNAi with the following target sequences: STAMBP sh#1 (5′- GCAGGATTGTAGGTTACTTAG-3′) and STAMBP sh#2 (5′-CAACTTAGATCTCCTGAAA-3′). Vectors encoding E2F1, PDK1, STAMBP, and mutated or truncated E2F1 were obtained from GenePharma. Subsequently, PC cells were transfected with the vectors and shRNA plasmids using Lipofectamine 3000 (Invitrogen) according to the instructions of the manufacturer.

### Chromatin immunoprecipitation assay (ChIP)

ChIP assays were conducted using the Millipore ChIP kit according to the manufacturer’s protocol. Briefly, 1.0 ×10^7^ cells were collected, fixed in 1% formaldehyde for 20 min, and quenched with glycine. The cells were sonicated using a Bioruptor sonicator (Diagenode) to produce DNA fragments of approximately 100–600 bp. Chromatin immunoprecipitates and input DNA were analyzed by qRT-PCR. Fold enrichment levels represented fold changes relative to the negative control for immunoglobulin G (IgG).

### Identification of extracellular acidification rate (ECAR) and oxygen consumption rate (OCR)

Based on the manufacturer’s guidelines, the Seahorse XFe24 extracellular flux analyzer (Seahorse Bioscience, Billerica, MA, USA) was employed to analyze cellular mitochondrial respiration and glycolytic capacity using the Glycolysis Stress Test Kit and XF Cell Mito stress test kit (Seahorse Bioscience), respectively.

### Subcutaneous xenograft transplantation

Pancreatic cancer cells (1 ×106 in 100 ml PBS) were injected subcutaneously into the flanks of nude mice (male BALB/c-nu/nu, 6–8 weeks old). The tumor volume (V) was calculated as follows:V = 0.52 × length × width2. Experimental nude mice were euthanized atthe end of the observation period, and then the tumors were removed and imaged. For the survival studies, animals were monitored for tumor volumes for 60 d, until the tumor volume exceeded 1000 mm^3^, or until the tumor became ulcerated with the ulcer diameter reaching 1 cm. All animal studies were approved by the Animal Experimental Ethics Committee of Nanchang University (NCUFII-2020523) and were performed in accordance with the NIH Guide for the Care and Use of Experimental Animals.

### Statistical analysis

All the results are presented as mean ± SD; they were examined through GraphPad Prism 8 (GraphPad Software, La Jolla, CA, USA) for a minimum of three independent trials. Survival analysis was performed using log-rank and Kaplan–Meier curves. Differences between groups were analyzed using analysis of variance (ANOVA) and two-tailed Student’s *t* test. Statistical significance was set at *P* < 0.05.

## Supplementary information

Supplementary Tables and Supplementary Figures

Original Data

## Data Availability

The data that support the findings of this study are available from the corresponding author upon reasonable request.
