# Supplementary material for: Identification of STAM-binding protein as a target for the treatment of gemcitabine resistance pancreatic cancer in a nutrient-poor microenvironment
Source: Cell Death Dis. 2024 Sep 6;15(9):657. doi: 10.1038/s41419-024-07048-z (PMC11379802; doi:10.1038/s41419-024-07048-z)
Supplement: Supplementary file 1 — Supplementary Tables and Supplementary Figures [file 41419_2024_7048_MOESM1_ESM.docx]

**Supplementary Tables and Supplementary Figures**

**Supplementary Table 1 List of Zn+-dependent JAMM deubiquitinases**

| MYSM1 | MPND | COPS6 | PRPF8 | EIF3H |
| --- | --- | --- | --- | --- |
| COPS5 | STAMBPL1 | BRCC3 | STAMBP | PSMD7 |
| PSMD14 |  |  |  |  |

| **Table S2 Relationship between STAMBP expression and clinicopathological features.** | | | | |
| --- | --- | --- | --- | --- |
|  |  |  |  |  |
| **Parameters** | **n** | **STAMBP expression** | | **P value** |
|  |  | **Low (N=45)** | **High (N=83)** |  |
| **Age (years)** |  |  |  | *P*=0.3581 |
| ≤65 | 50 | 20 | 30 |  |
| ＞65 | 78 | 25 | 53 |  |
| **Sex** |  |  |  | *P=0.3319* |
| Female | 58 | 23 | 35 |  |
| Male | 70 | 22 | 48 |  |
| **Tumour size (cm)** |  |  |  | ***P=0.007*** |
| ＜5 | 85 | 23 | 62 |  |
| ≥5 | 43 | 22 | 21 |  |
| **Tumour stage** |  |  |  | ***P=0.0035*** |
| T1-T2 | 49 | 22 | 28 |  |
| T3-T4 | 79 | 13 | 55 |  |
| **Distant metastasis** |  |  |  | *P=0.9253* |
| No | 90 | 30 | 60 |  |
| Yes | 38 | 13 | 25 |  |
| **Histologic grade** |  |  |  | *P=0.2547* |
| High | 45 | 19 | 26 |  |
| Low to medium | 83 | 27 | 57 |  |
| **Differentiation** |  |  |  | ***P=0.0127*** |
| Well | 55 | 26 | 29 |  |
| Moderate/poor | 73 | 19 | 54 |  |

| **Table S3. Univariate and multivariate analyses of overall survival in PC patients** | | | | | | |
| --- | --- | --- | --- | --- | --- | --- |
| **Parameters** | **Univariate analysis** |  |  | **Multivariate analysis** |  |  |
|  | **HR** | **95%CI** | ***P* value** | **HR** | **95%CI** | ***P* value** |
| **Age**  (≥65 vs ＜65) | 1.532 | 0.652-1.735 | 0.755 | — | — | — |
| **Sex**  (Female vs Male) | 1.751 | 0.792-2.034 | 0.539 | — | — | — |
| **Distant metastasis**  (No vs Yes) | 1.369 | 0.539-2.865 | 0.391 | — | — | — |
| **TNM stage**  (T1-T2 vs T3-T4) | 1.831 | 1.385-3.122 | 0.081 | — | — | — |
| **Tumor size**  (＜5 vs ≥6) | 1.799 | 1.551-3.866 | 0.016* | 1.378 | 1.107-2.325 | 0.043* |
| **Histologic grade**  (High vs Low to medium) | 2.834 | 1.636-4.339 | 0.003* | 1.651 | 1.389-3.531 | 0.011* |
| **Differentiation**  (Well vs Moderate/Poor) | 1.839 | 1.365-3.312 | 0.039* | 1.110 | 0.895-2.241 | 0.308 |
| **STAMBP expression**  (High vs Low) | 5.037 | 3.045-5.039 | 0.001* | 2.245 | 1.851-3.695 | 0.005* |
|  |  |  |  |  |  |  |

**Table S4. 2D image of FDA small molecules and interaction forces obtained through STAMBP protein screening**

| No. | Compound name | CAS | Docking  score | Structure |
| --- | --- | --- | --- | --- |
| 1 | Avatrombopag | 570406-98-3 | -8.616 | 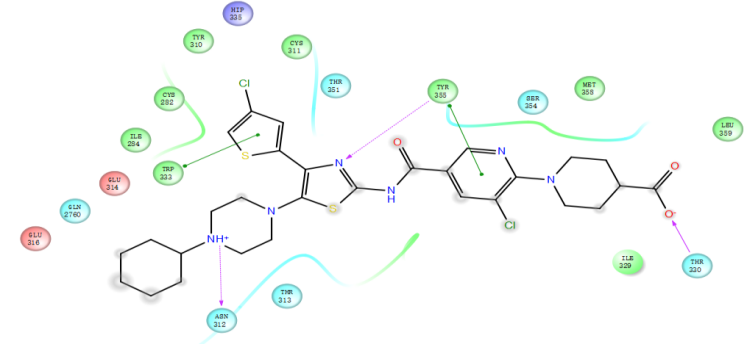 |
| 2 | Entrectinib | 1108743-60-7 | -7.957 | 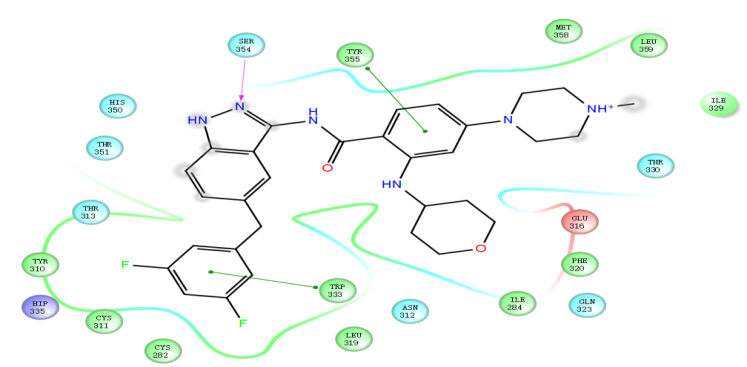 |
| 3 | Ranolazine | 95635-55-5 | -7.770 | 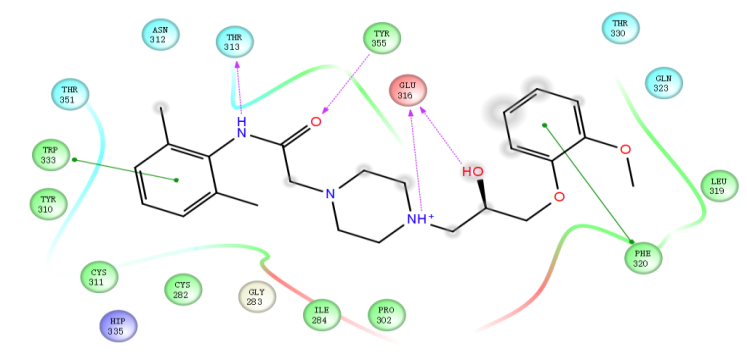 |
| 4 | DCC-2618 | 1442472-39-0 | -7.389 | 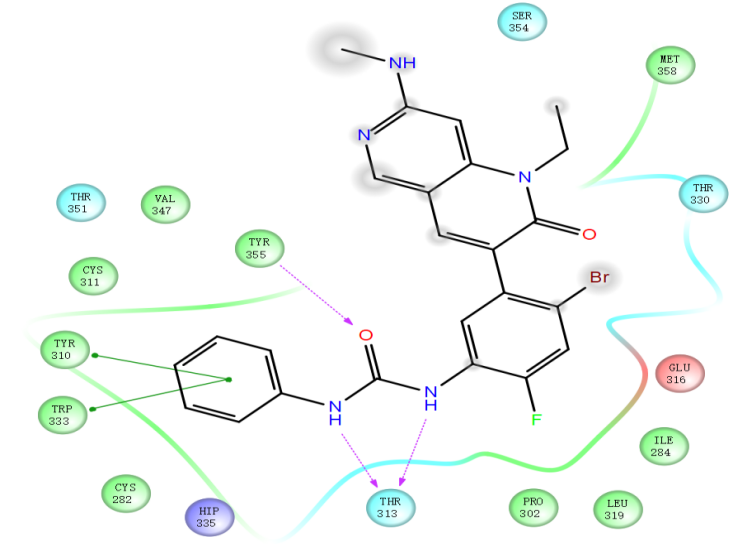 |
| 5 | Olmutinib | 1353550-13-6 | -7.272 | 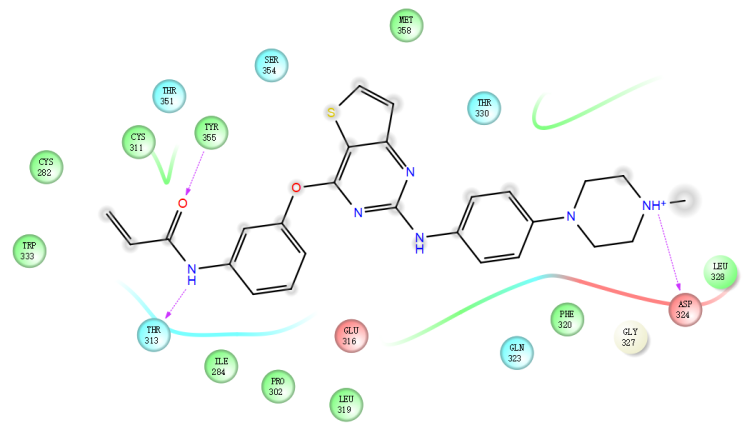 |
| 6 | Levonebivolol | 118457-14-0 | -7.178 | 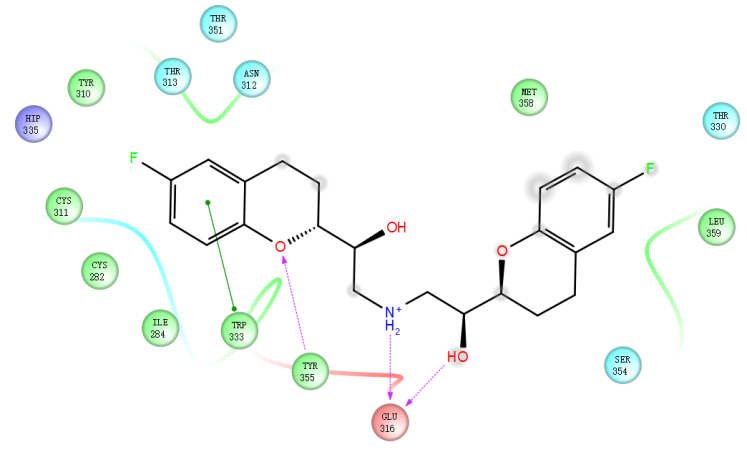 |
| 7 | Dasatinib | 854001-07-3 | -7.168 | 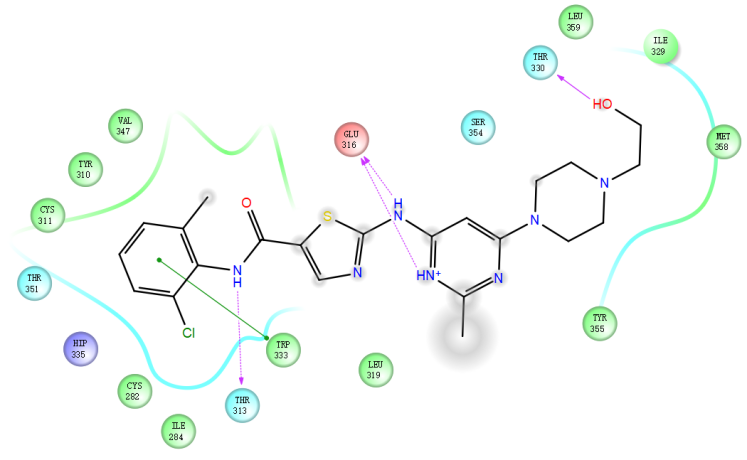 |
| 8 | Ivosidenib | 1448346-63-1 | -7.157 | 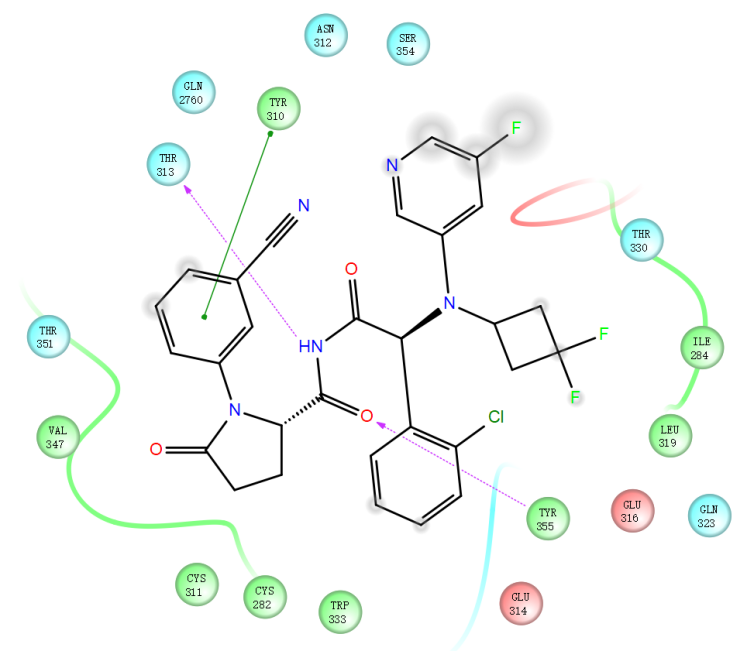 |
| 9 | Desfesoterodine | 207679-81-0 | -7.150 | 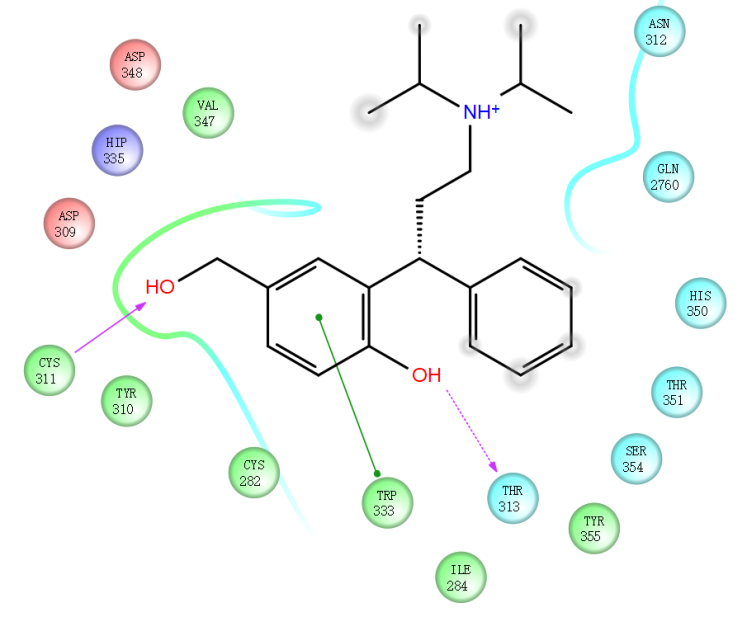 |
| 10 | Dasatinib | 302962-49-8 | -7.149 | 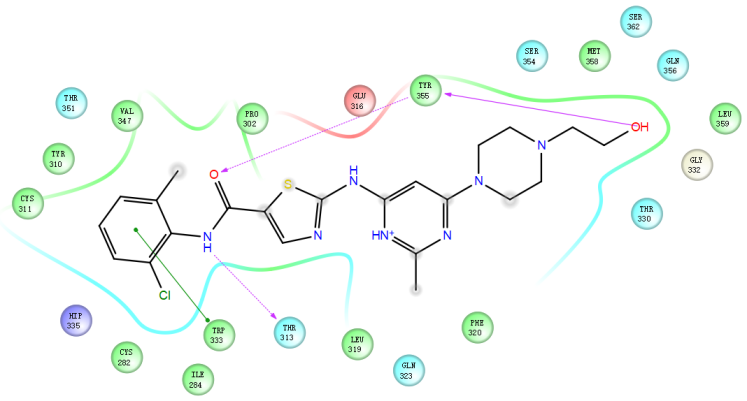 |
| 11 | Zanubrutinib | 1691249-45-2 | -7.048 | 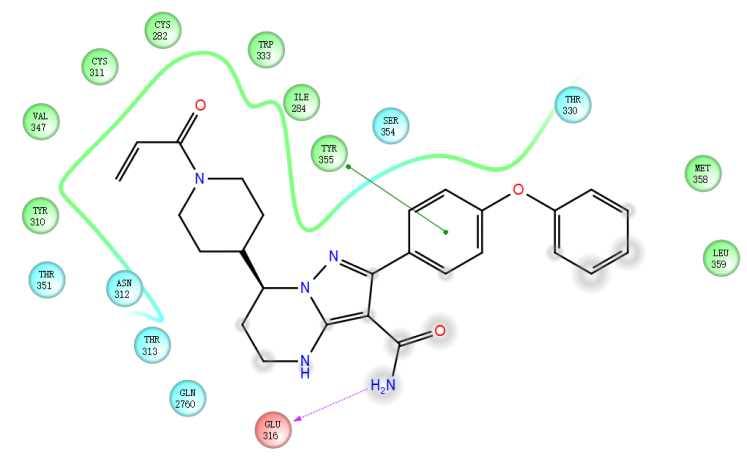 |
| 12 | Vilazodone | 163521-08-2 | -7.006 | 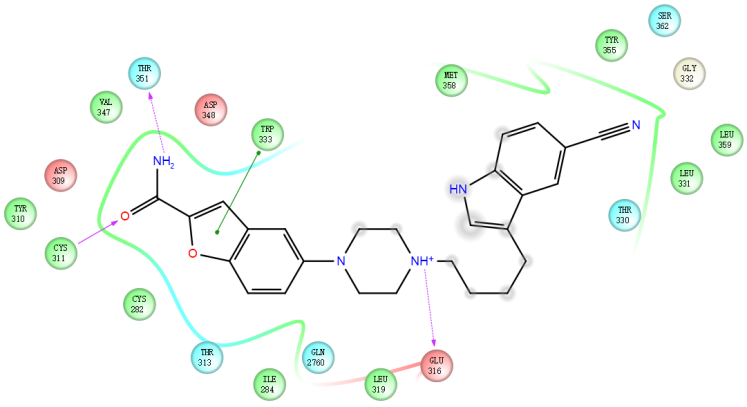 |
| 13 | CB-5083 | 1542705-92-9 | -6.961 | 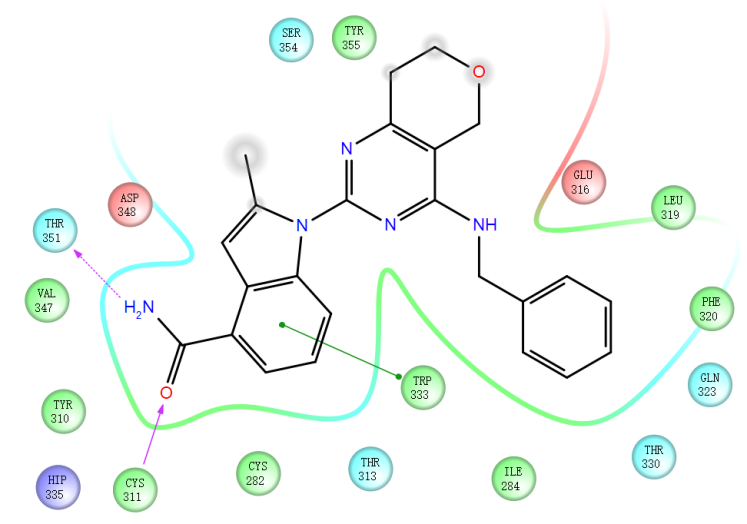 |
| 14 | Frovatriptan | 158930-09-7 | -6.949 | 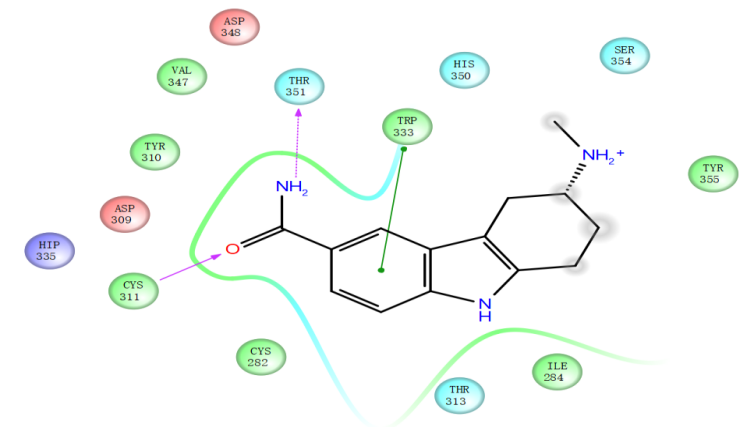 |
| 15 | Nilotinib | 641571-10-0 | -6.821 | 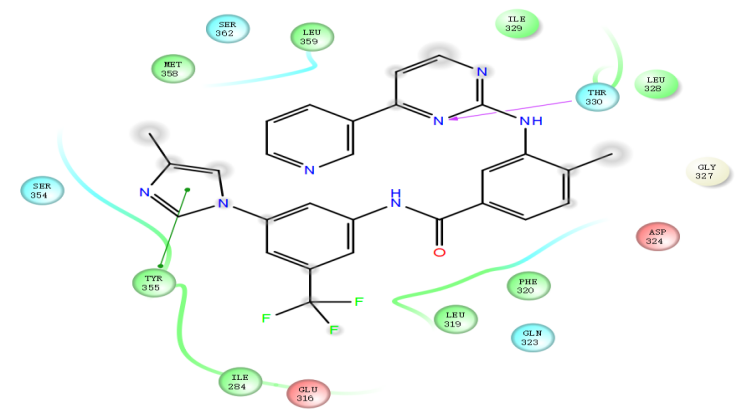 |
| 16 | Brigatinib | 1197953-54-0 | -6.810 | 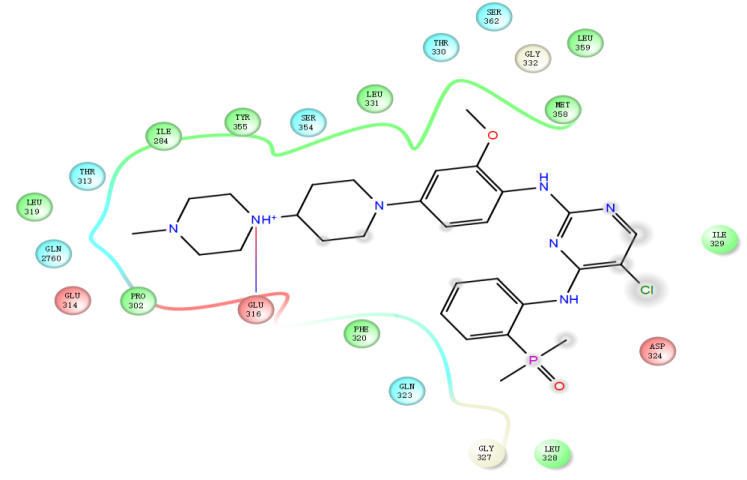 |
| 17 | Canagliflozin | 842133-18-0 | -6.770 | 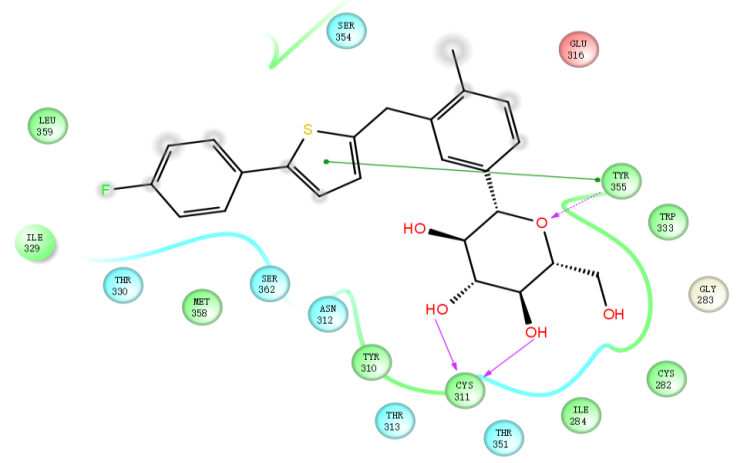 |
| 18 | Nicarbazin | 330-95-0 | -6.766 | 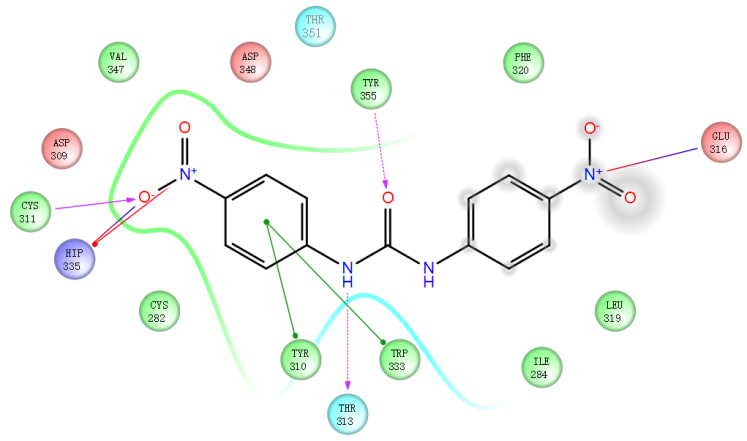 |
| 19 | Baricitinib | 1187594-09-7 | -6.756 | 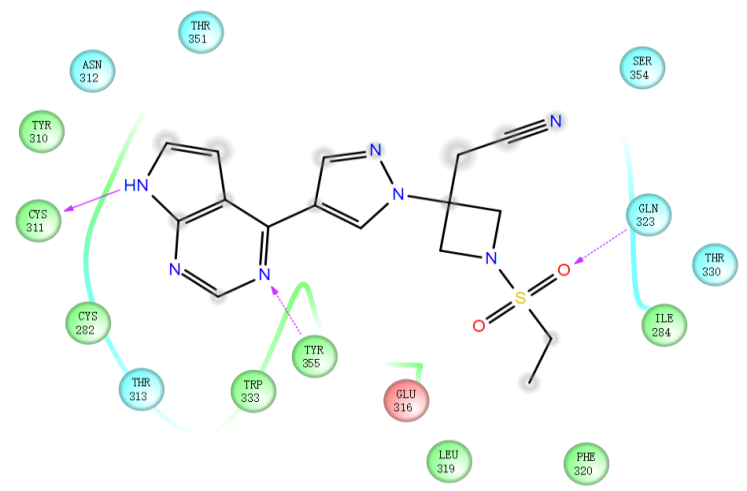 |
| 20 | Plerixafor | 110078-46-1 | -6.754 | 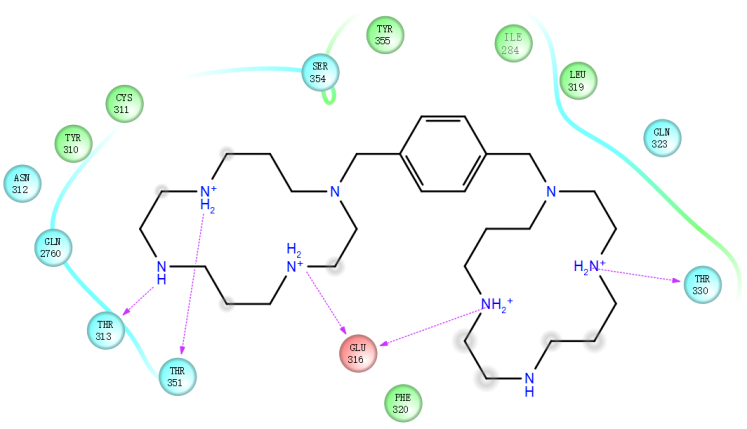 |

**Supplementary Figures**

**
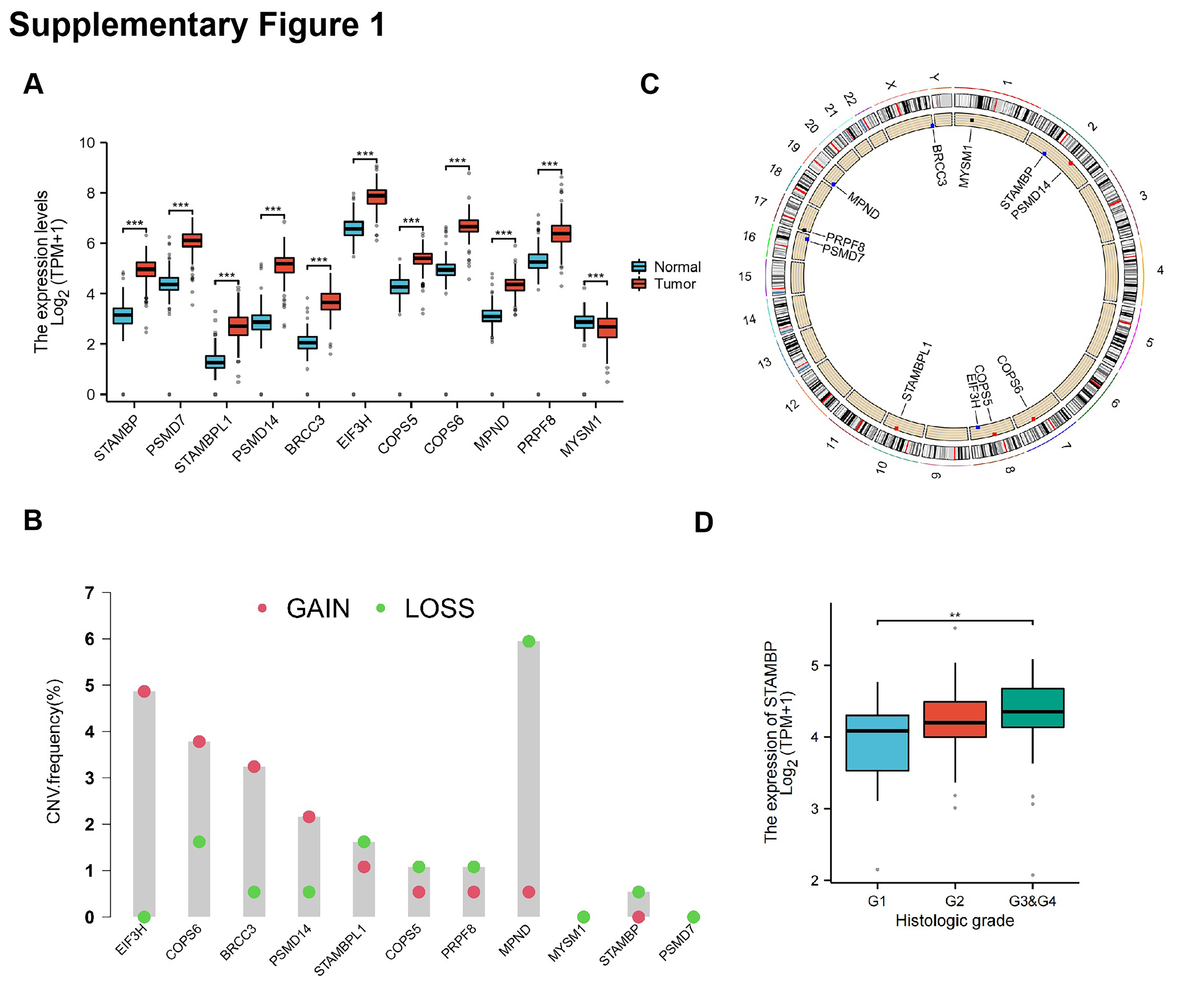
**

**Supplementary Figure 1. STAMBP may serve as a valuable new prognostic factor for human PC**. A, Analysis of the 11 differentially expressed Zn^+^-dependent JAMM deubiquitinases in PC tissues and the normal tissue from the TCGA-PAAD dataset. ^***^*P*<0.001. B, Expression of STAMBP progressively increases with the increase of PC grades. ^**^*P*<0.01. C, The CNV variation frequency of Zn^+^-dependent JAMM deubiquitinases in TCGA-PAAD cohort. The height of the column represented the alteration frequency. The deletion frequency, blue dot; The amplification frequency, red dot. D, The location of CNV alteration of Zn^+^-dependent JAMM deubiquitinases on 23 chromosomes using TCGA-PAAD cohort.

**
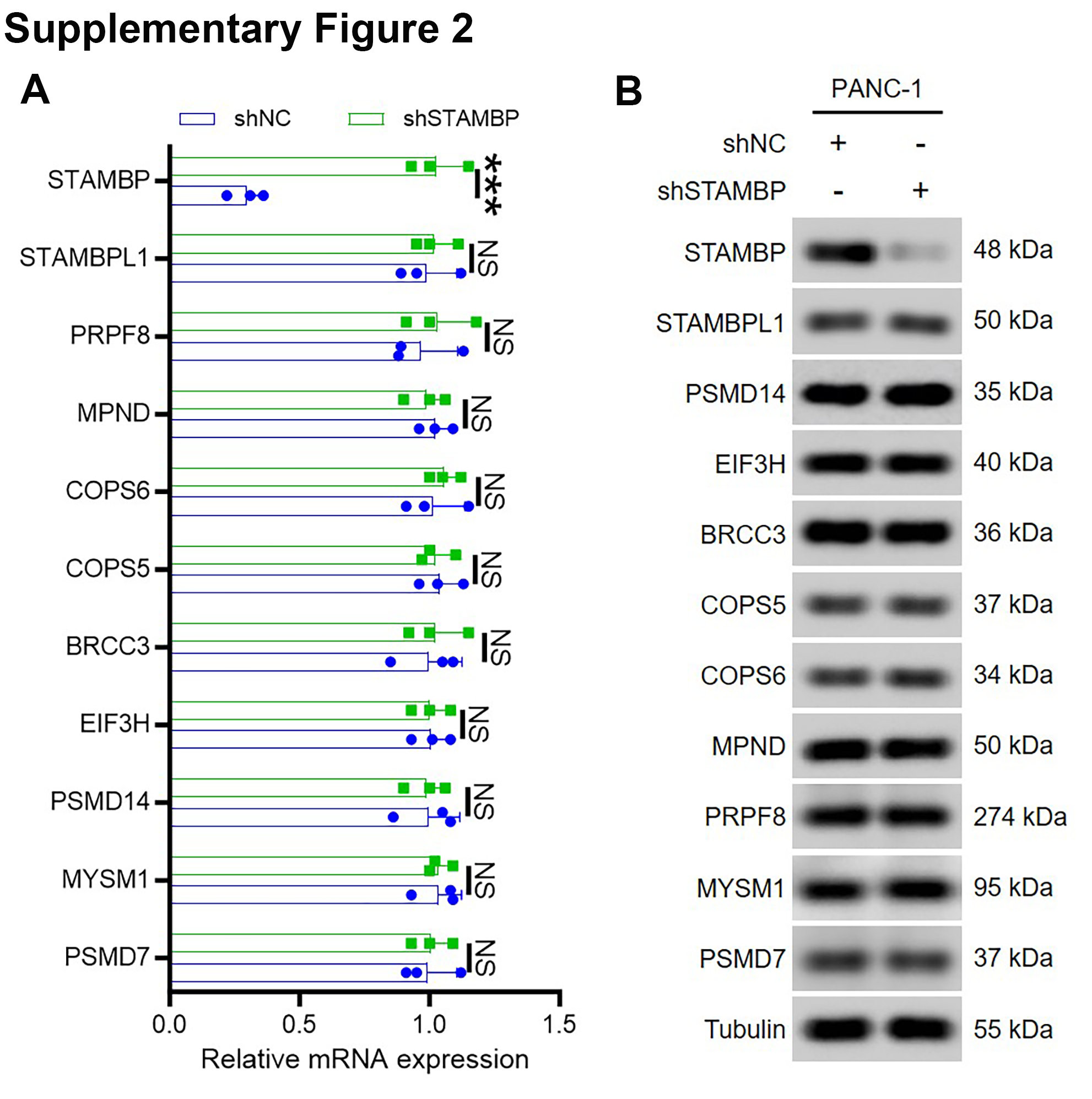
**

**Supplementary Figure 2. Knockdown STAMBP does not affect other proteins in the Zn+-dependent JAMM deubiquitinases family**

A, The mRNA levels of the Zn+-dependent JAMM deubiquitinases family in the PANC-1/shSTAMBP cells were detected by qRT-PCR. ^***^*P*<0.001. B, The protein levels of the Zn+-dependent JAMM deubiquitinases family in the PANC-1/shSTAMBP cells were detected by western blot.

**
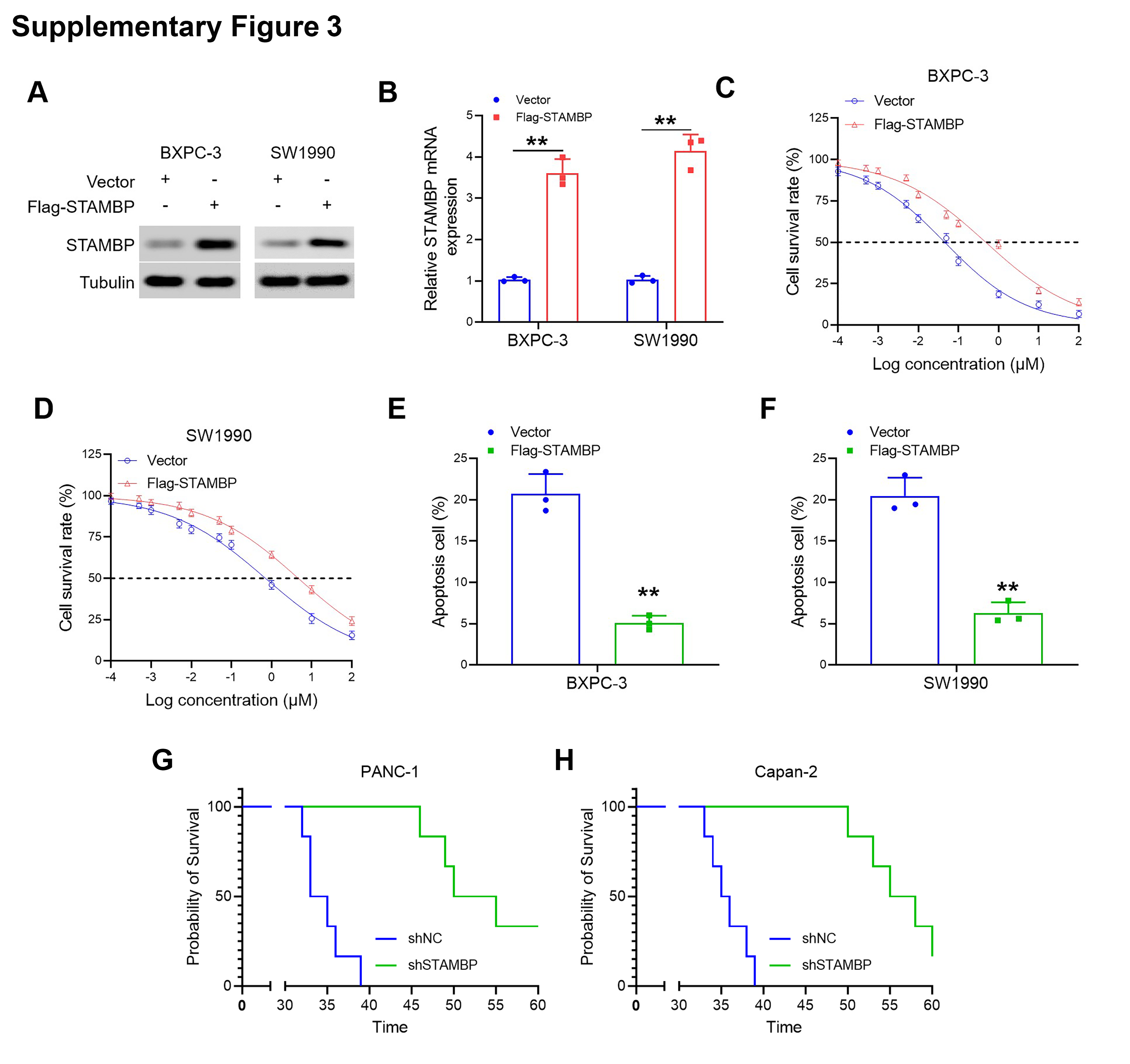
**

**Supplementary Figure 3. Upregulation of STAMBP decreased the chemotherapy sensitivity of PC to GEM.** A, western blot showing the protein expression of STAMBP in gemcitabine-resistant (GR) PC cells following transfected with vector or Flag-STAMBP. Tubulin was a loading control. B, qRT-PCR assay showing the mRNA level of STAMBP in gemcitabine-resistant (GR) PC cells following transfected with vector or Flag-STAMBP. ^**^*P*<0.01. C and D, IC50 value of gemcitabine in BxPC-3/GR (C) and SW1990/GR (D) cells transfected with vector or Flag-STAMBP by the CCK-8 assay. E and F, the quantification of cell apoptosis assay in PANC-1/GR (E) and Capan-2/GR (F) cells transfected with or without shSTAMBP by flowcytometry, under gemcitabine treatment (2 µM, 48 h). G, Kaplan–Meier survival curves for the shNC+GEM and shSTAMBP+GEM group.


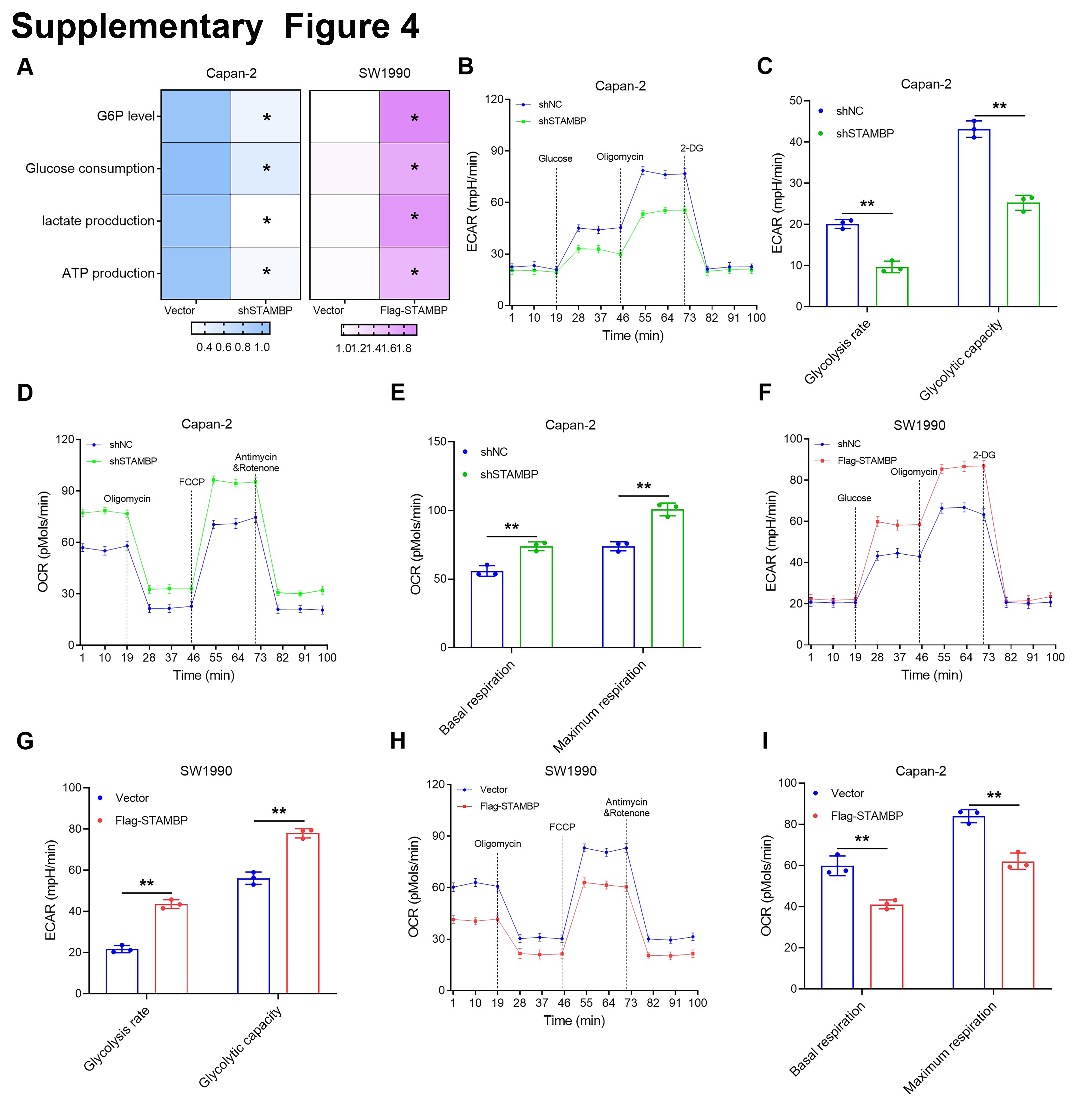


**Supplementary Figure 4. STAMBP enhanced aerobic glycolysis in PC cells.** A, G6P levels, glucose uptake, the production of lactate and ATP production were measured in Capan-2/shSTAMBP or SW1990/Flag-STAMBP cells. ^*^*P*<0.05. B and C, ECAR data showing the glycolytic rate and capacity in Capan-2/shSTAMBP cells. ^**^*P*<0.01. D and E, OCR results showing the basal respiration and maximum respiration in Capan-2/shSTAMBP cells. ^**^*P*<0.01. F and G, ECAR data showing the glycolytic rate and capacity in SW1990/Flag-STAMBP cells. ^**^*P*<0.01. H and I, OCR results showing the basal respiration and maximum respiration in SW1990/Flag-STAMBP cells. ^**^*P*<0.01.

**
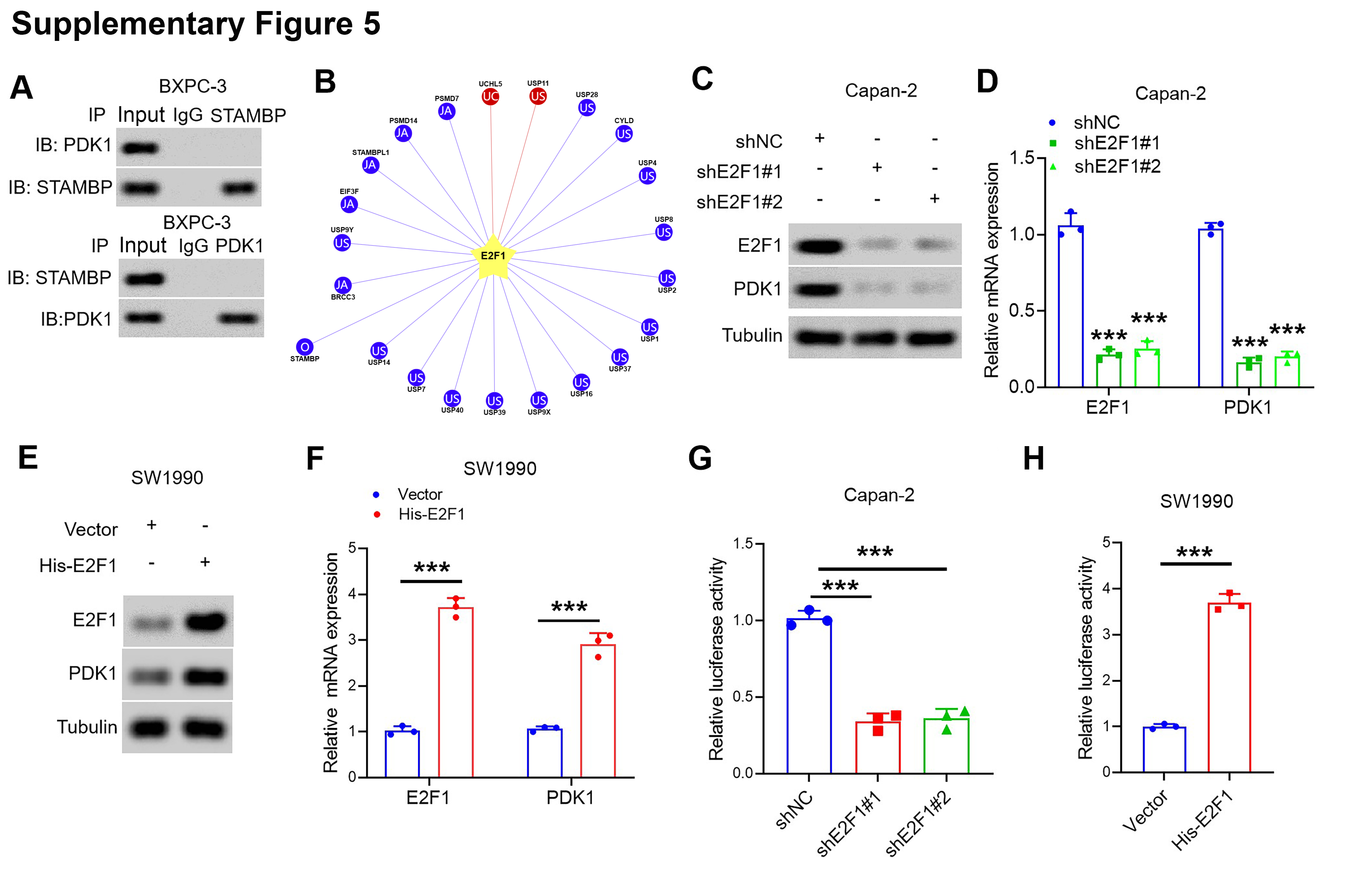
**

**Supplementary Figure 5. E2F1 is a transcription factor of PDK1 in PC cells.** A, Co-IP assay showing that endogenous STAMBP and PDK1 were not directly bound in BxPC-3 cell. B and C, the protein (B) and mRNA level (C) of E2F1 and PDK1 were detected in Capan-2/shE2F1 cells. Tubulin was a loading control. ^***^*P*<0.001. D and E, the protein (D) and mRNA level (E) of E2F1 and PDK1 were detected in SW1990/His-E2F1 cells. Tubulin was a loading control. ^***^*P*<0.001. F and G, Full-length PDK1 promoter luciferase construct was transfected into the Capan-2/shE2F1 cells (F) and SW1990/His-E2F1 (G) cells. Transcriptional activation was analyzed with the dual luciferase reporter assay. ^***^*P*<0.001 vs control group.

**
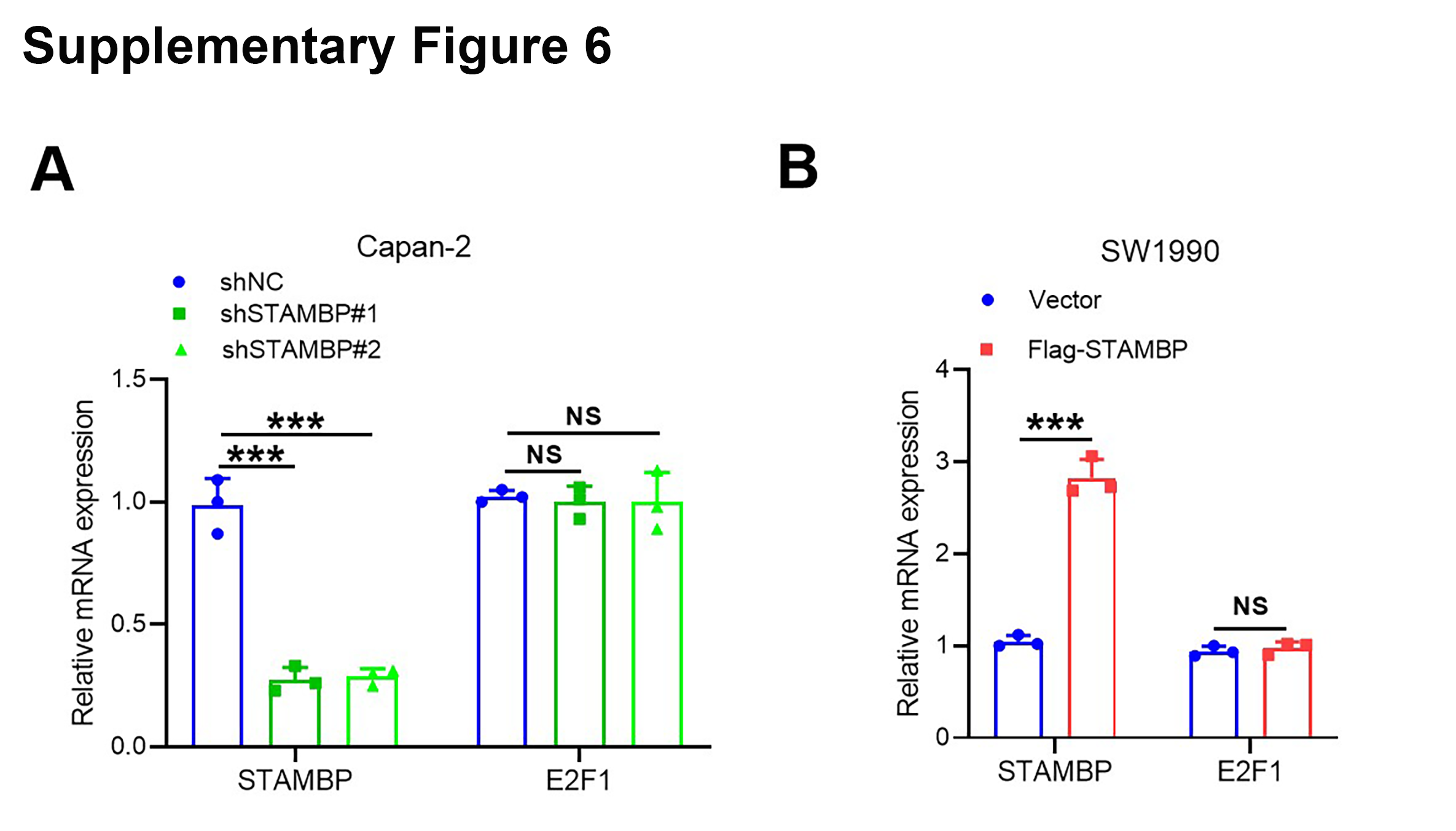
**

**Supplementary Figure 6. E2F1 mRNA levels did not influence the variation in STAMBP expression in PC cells.** A, the mRNA levels of STAMBP and E2F1 in the Capan-2/shE2F1 cells were detected by qRT-PCR. ^***^*P*<0.001. B, the mRNA levels of STAMBP and E2F1 in SW1990/His-E2F1 cells were detected by qRT-PCR. ^***^*P*<0.001.


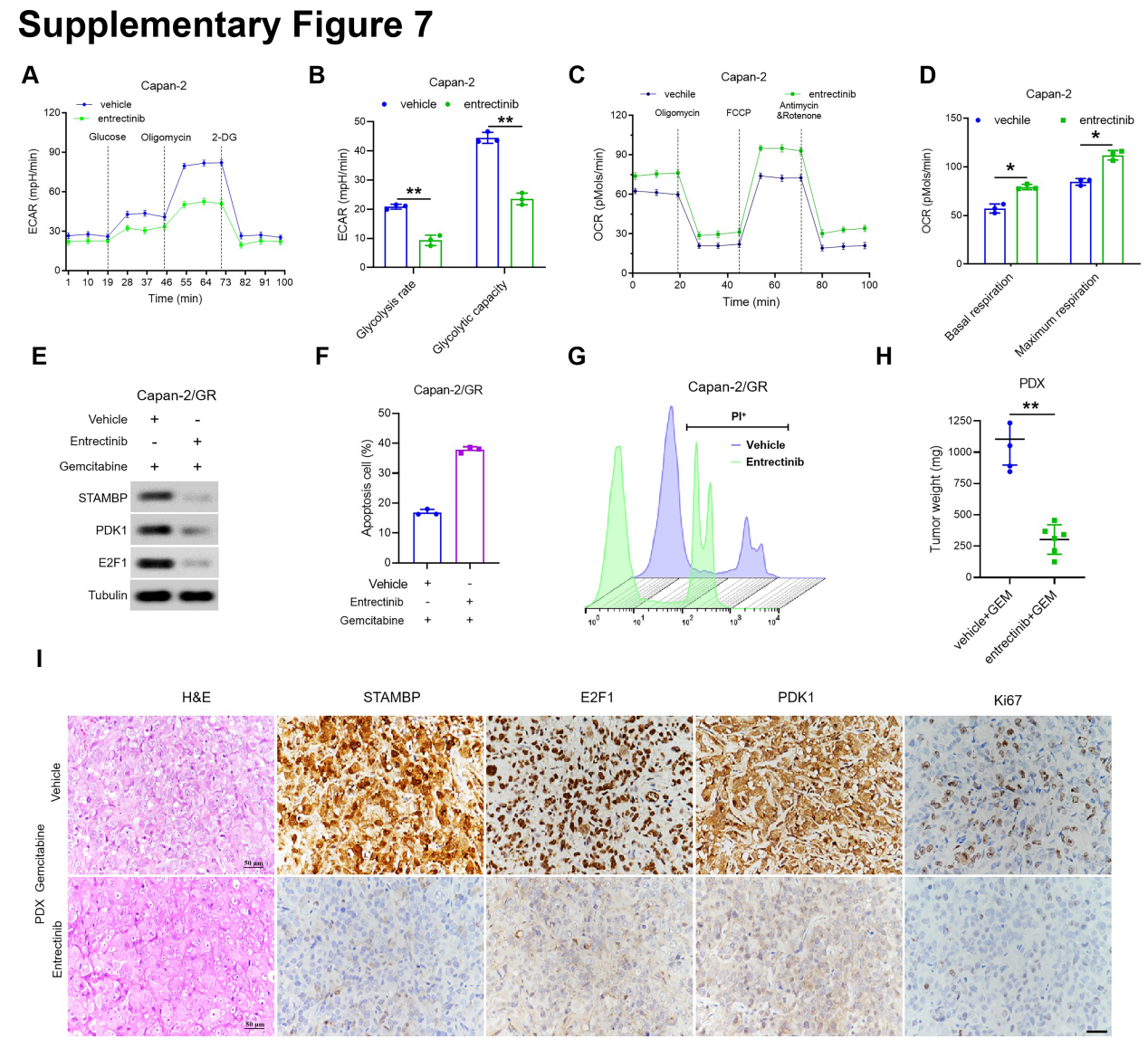


**Supplementary Figure 7. Entrectinib increased GEM sensitivity in PC by inhibiting the activity of STAMBP.** A and B, ECAR data showing the glycolytic rate and capacity in Capan-2 cells following treatment with the entrectinib. ^**^*P*<0.01. C and D, OCR results showing the basal respiration and maximum respiration in Capan-2 cells following treatment with the entrectinib. ^*^*P*<0.05. E, the protein levels of STAMBP, PDK1, and E2F1 in Capan-2-G/R cells were detected after treatment with the entrectinib. F and G, Representative images (left) and quantification (right) of PI-positive cell population in the indicated cells. ^**^*P*<0.01. H, tumor wight in PDXs treated with entrectinib, gemcitabine, or both. ^**^*P*<0.01. I, Representative images of H&E, STAMBP, E2F1, PDK1, and Ki-67 staining of the tumor tissues in PDXs treated with entrectinib, gemcitabine, or both. Scale bar, 50 μm.
