# Supplementary material for: Identification of STAM-binding protein as a target for the treatment of gemcitabine resistance pancreatic cancer in a nutrient-poor microenvironment
Source: Cell Death Dis. 2024 Sep 6;15(9):657. doi: 10.1038/s41419-024-07048-z (PMC11379802; doi:10.1038/s41419-024-07048-z)

Full and uncropped western blot for Figure 1H

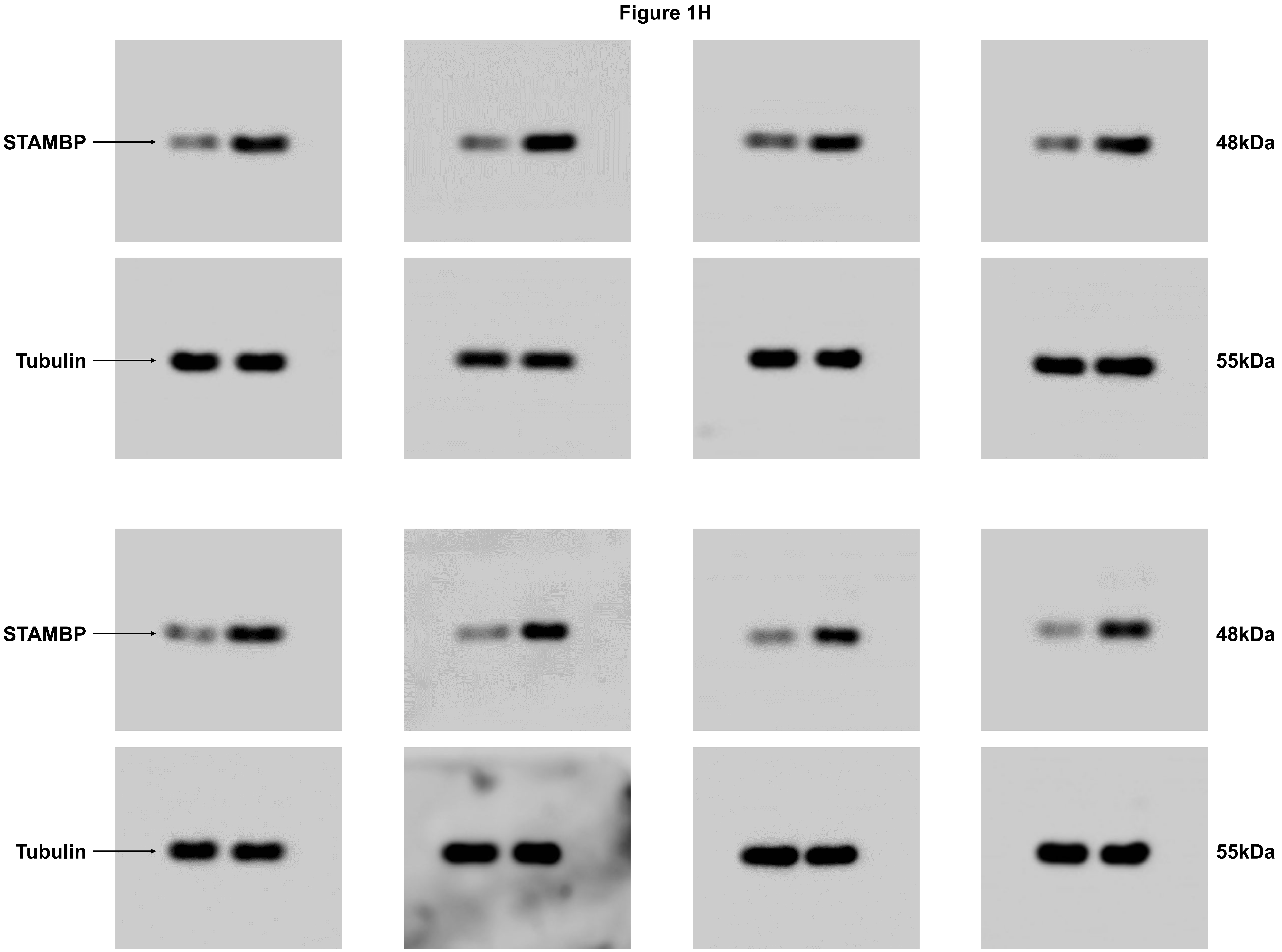

Full and uncropped western blot for Figure 2

Figure 2B

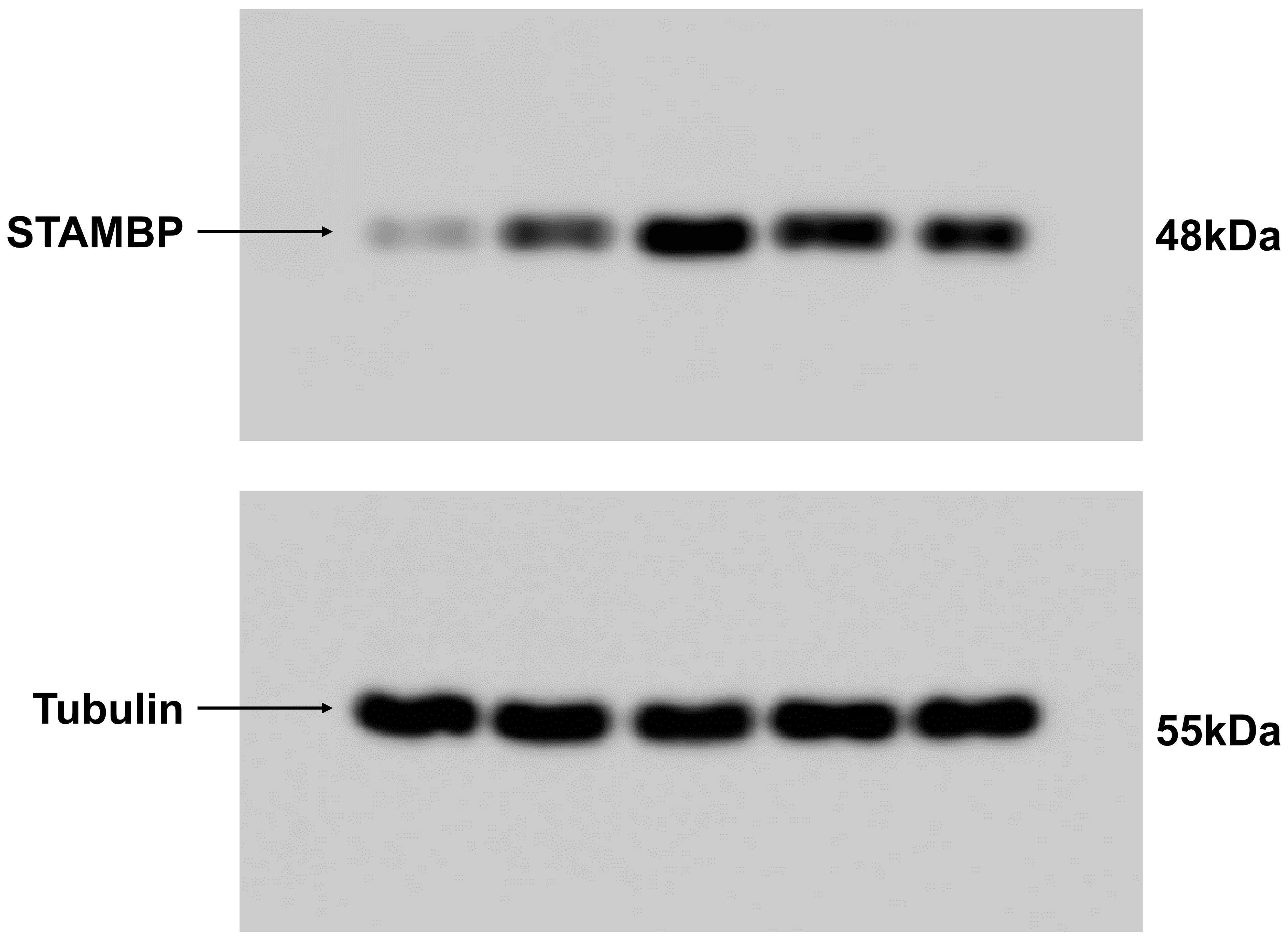

Figure 2H

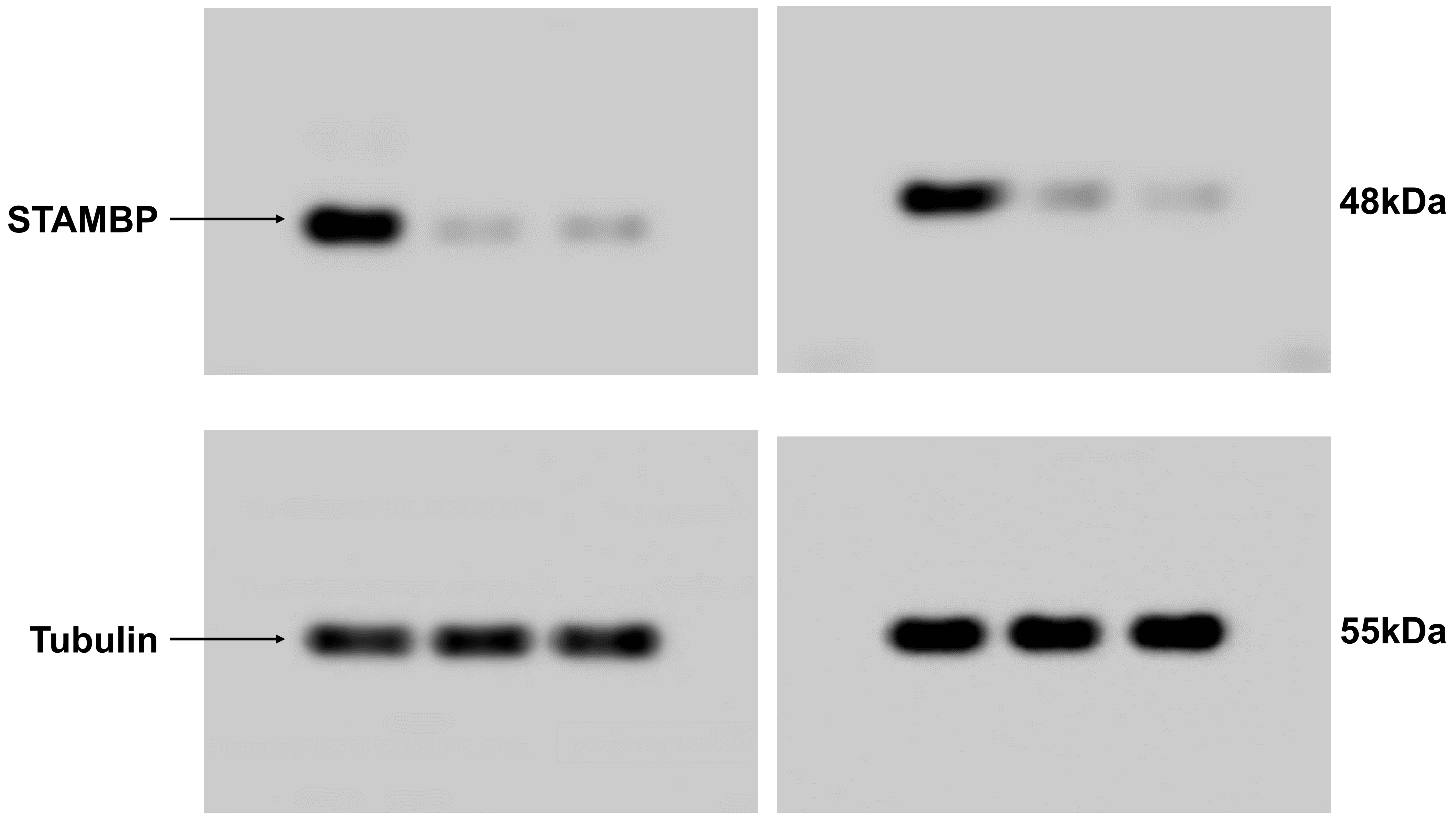

Figure 2F

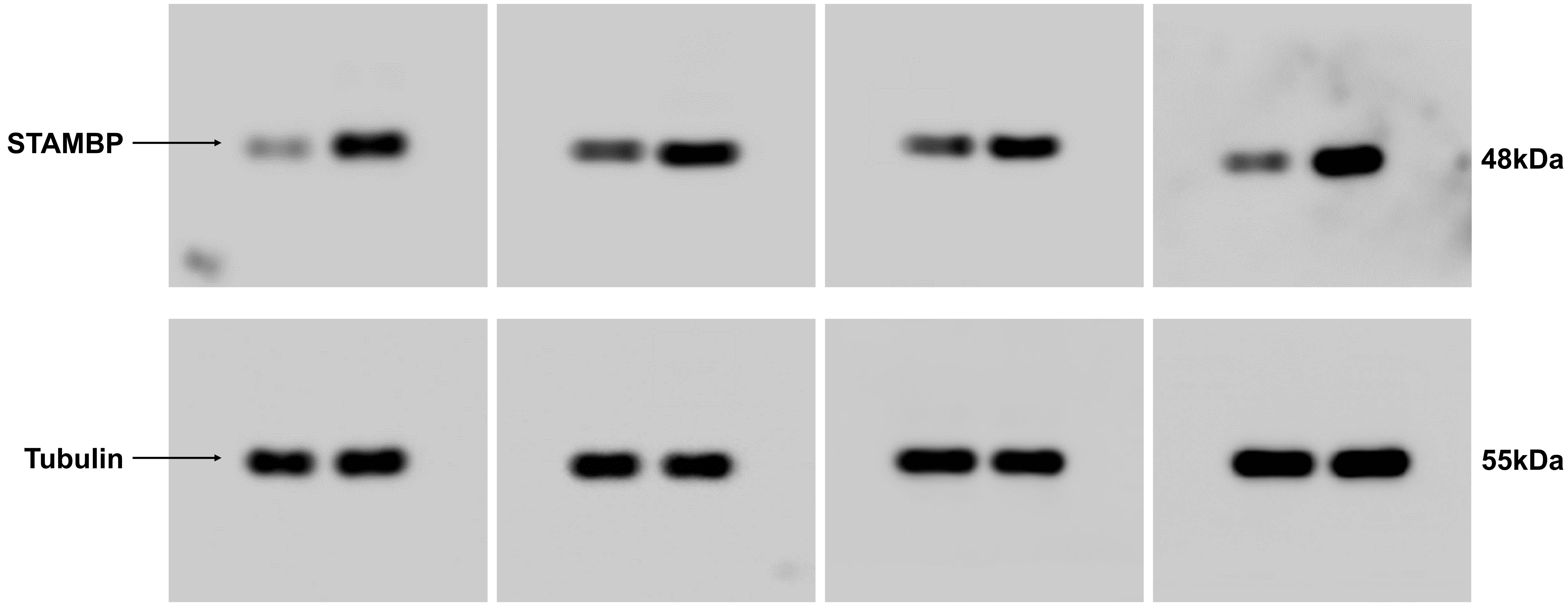

Full and uncropped western blot for Figure 4

Figure 4B (left) Figure 4B (right)

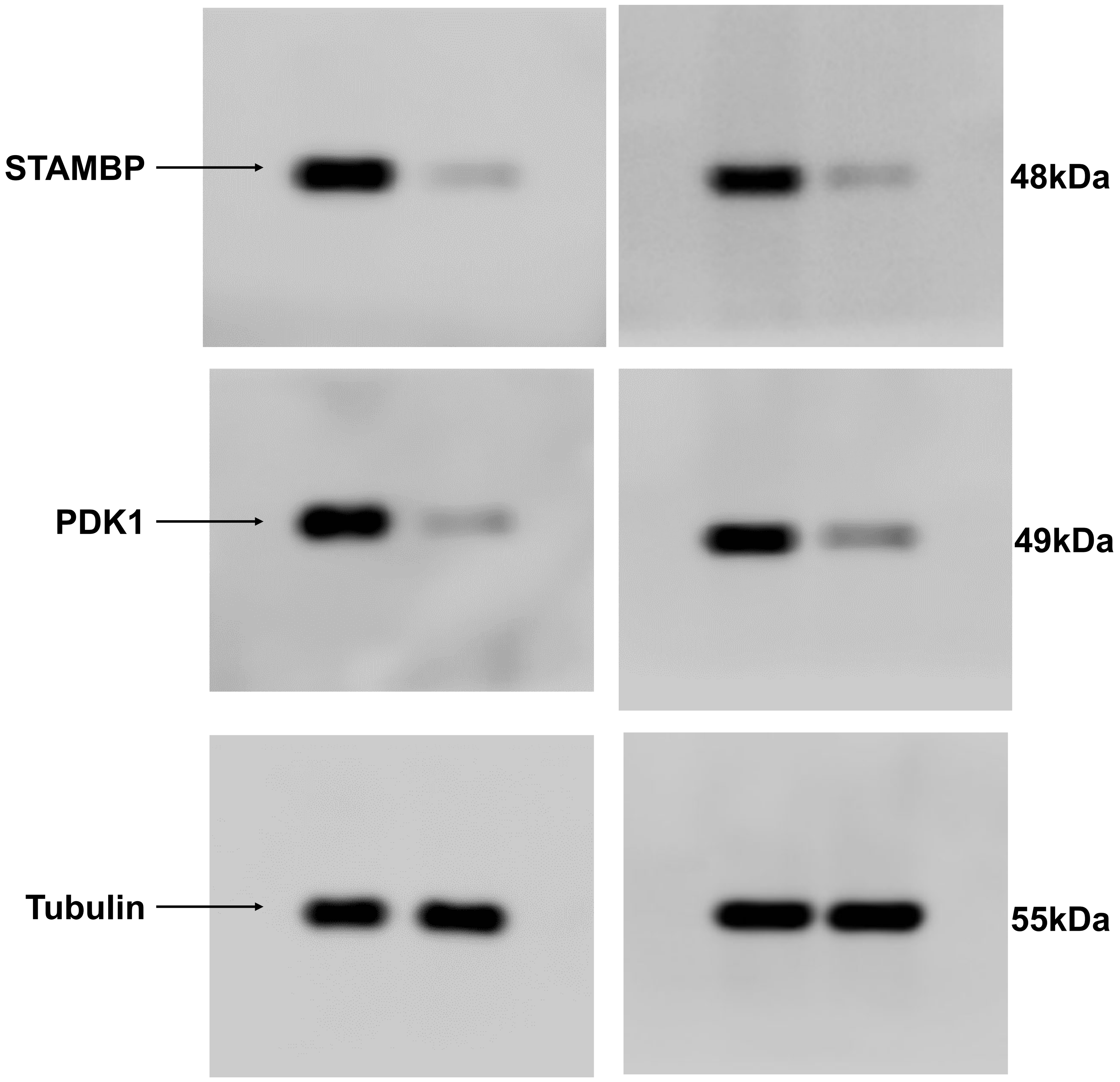

Figure 4D (left) Figure 4D (right)

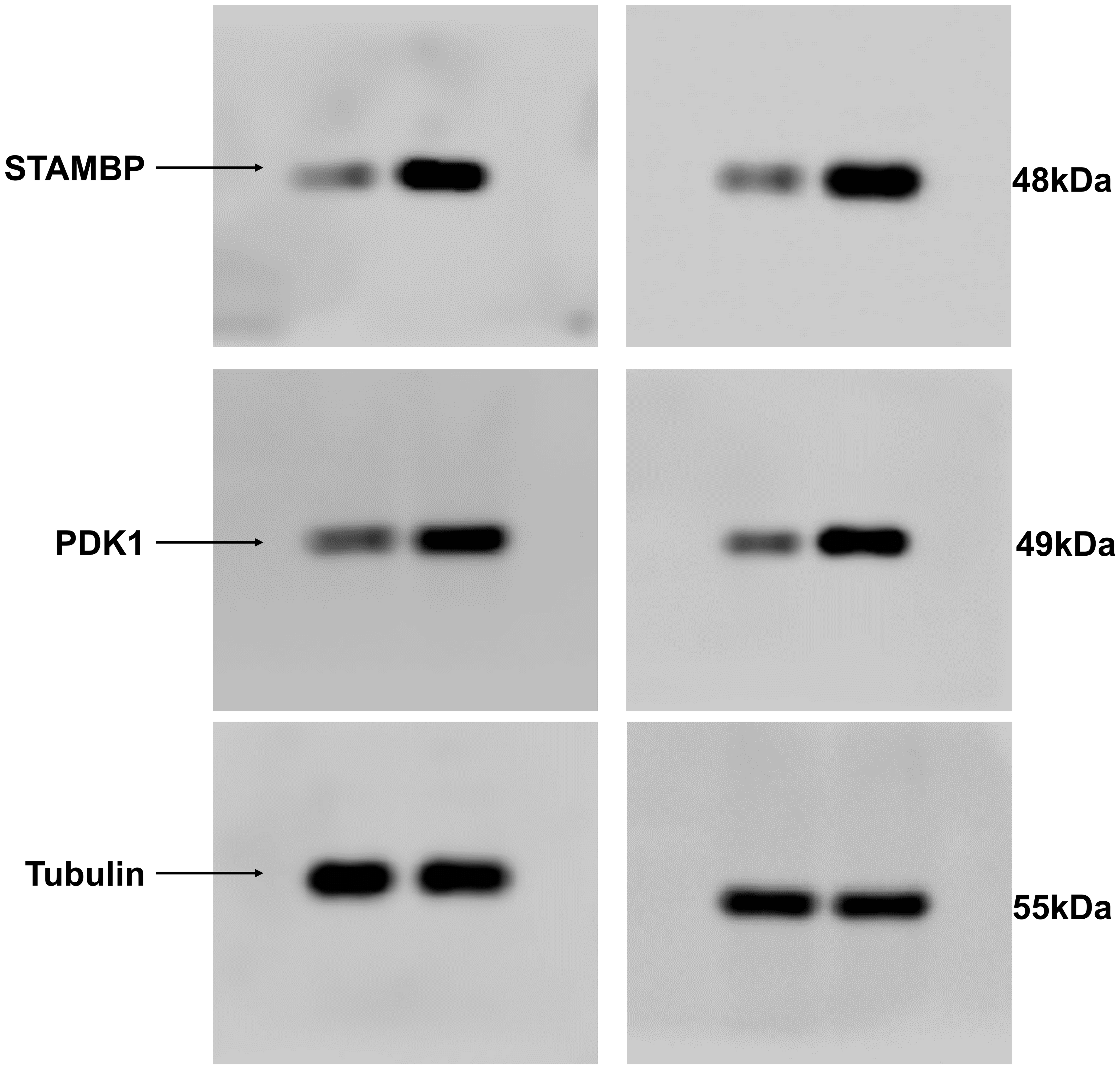

Figure 4F

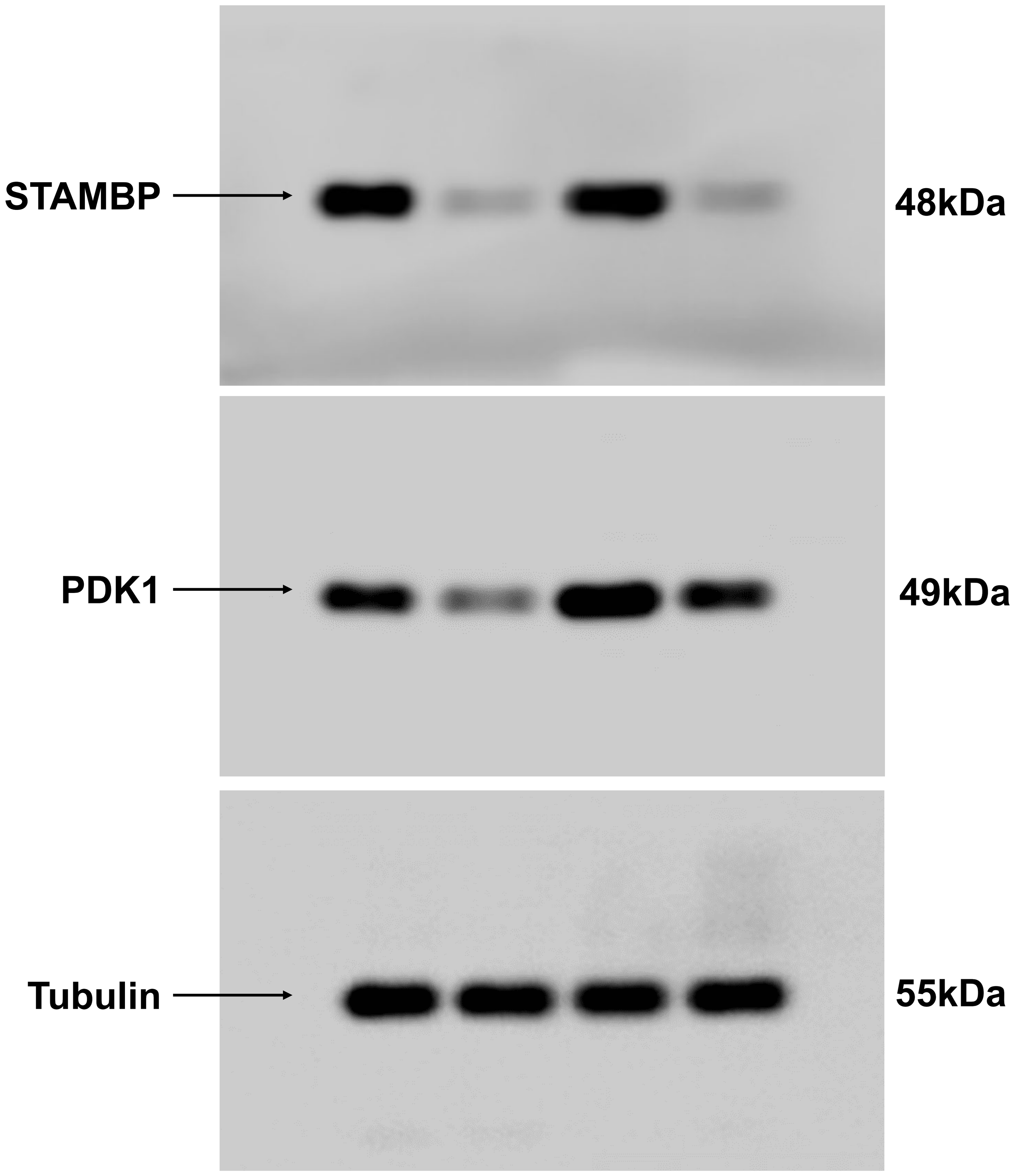

Figure 4M

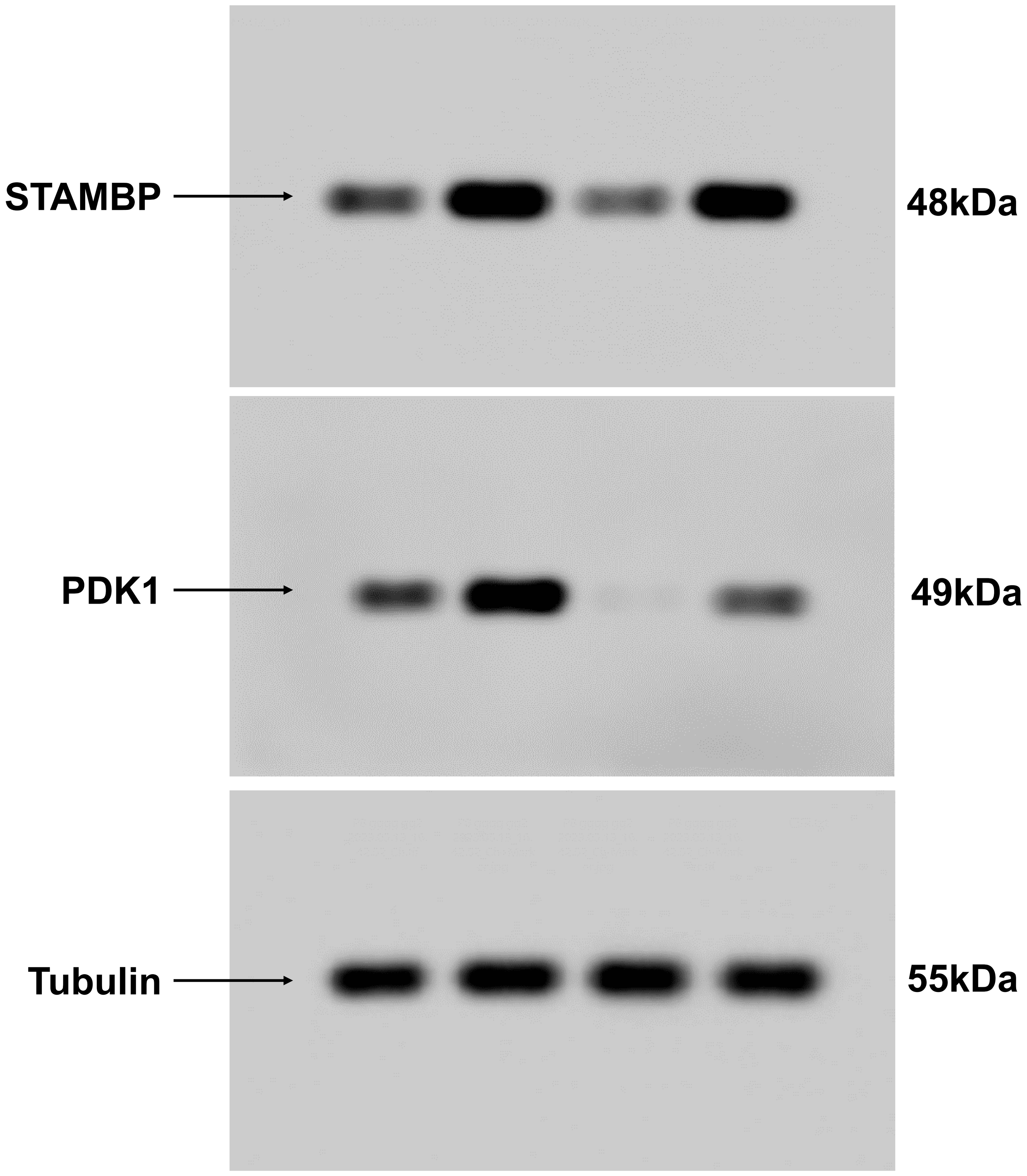

Full and uncropped western blot for Figure 5

Figure 5A (top)

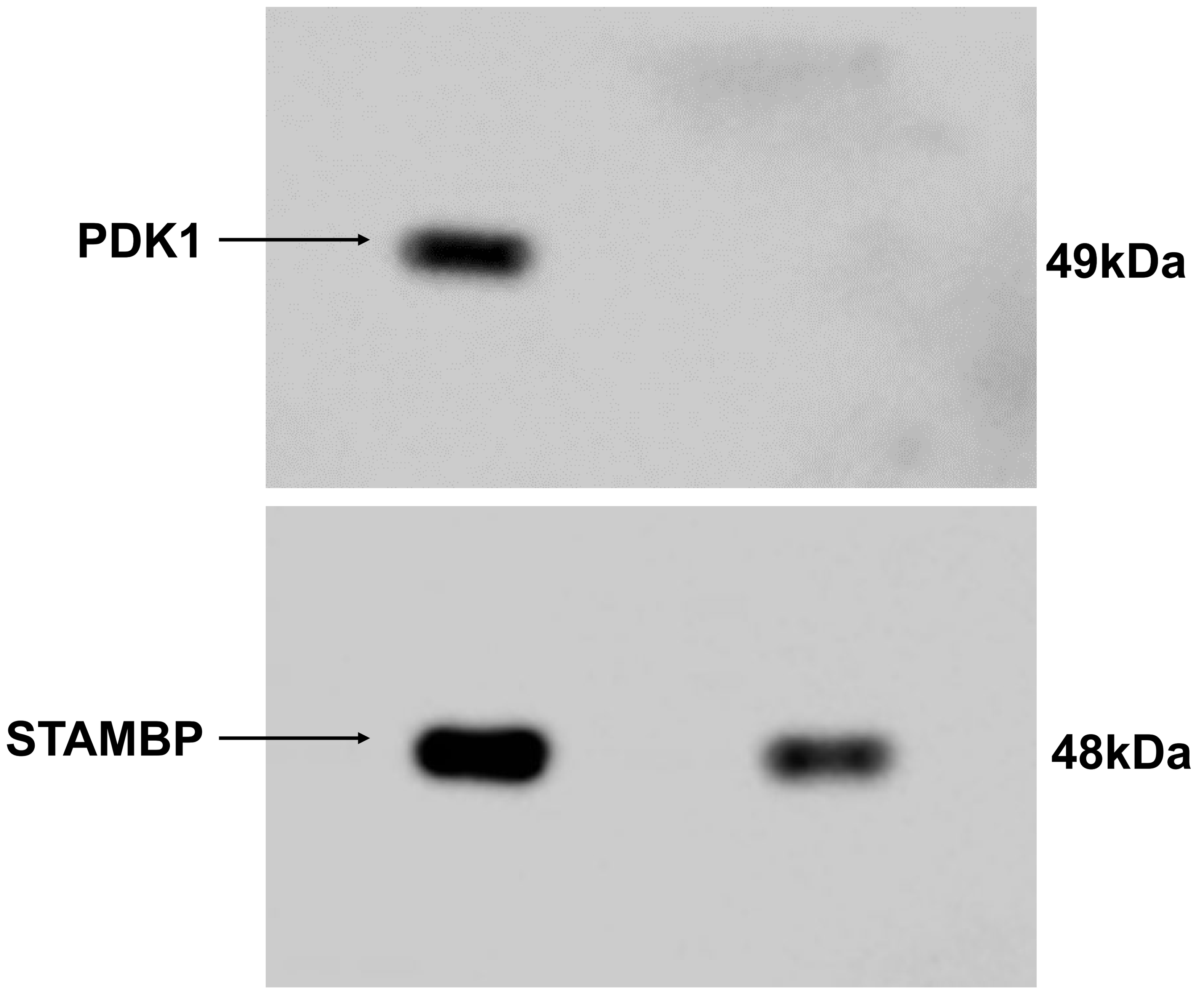

Figure 5A (bottom)

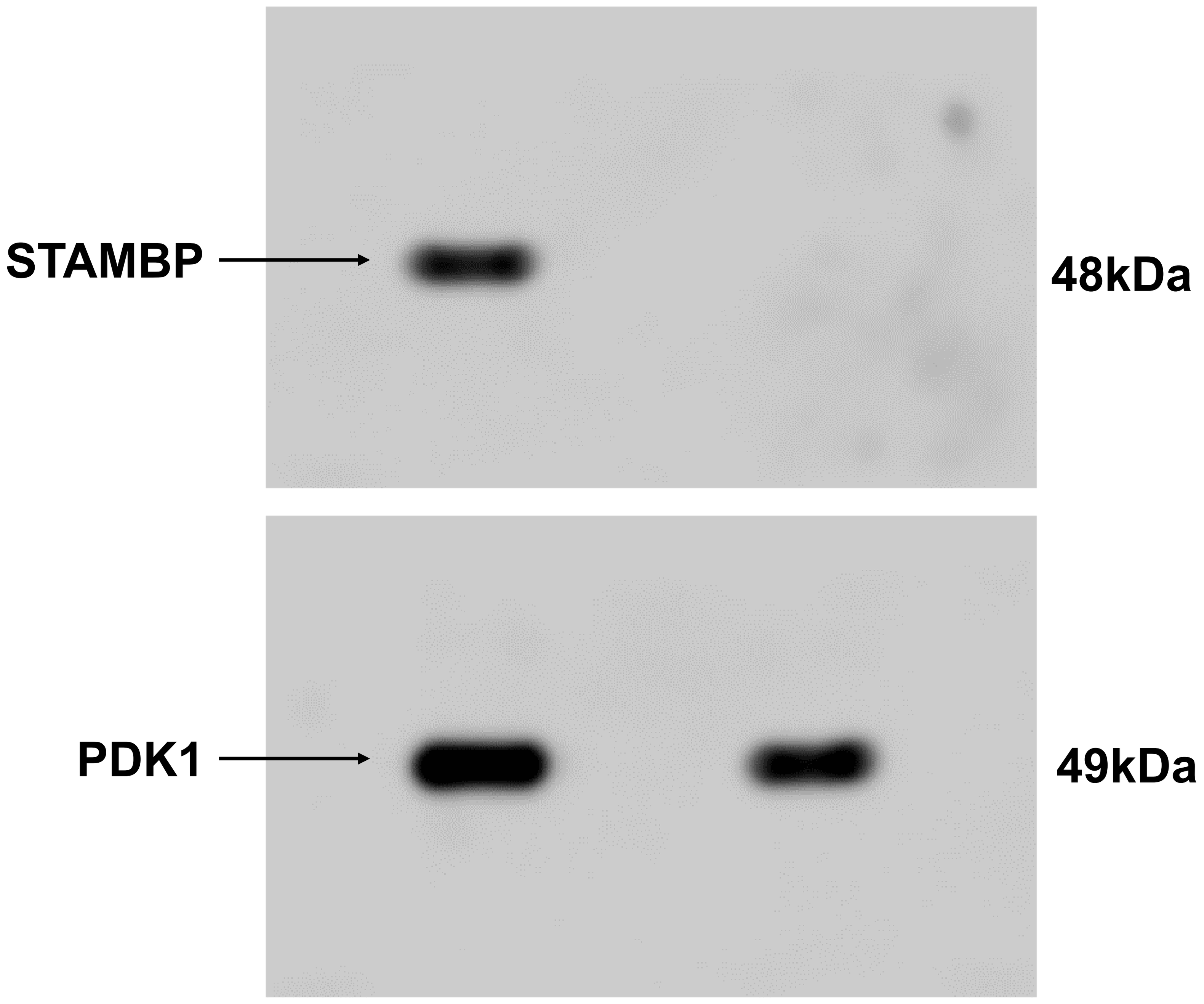

Figure 5E

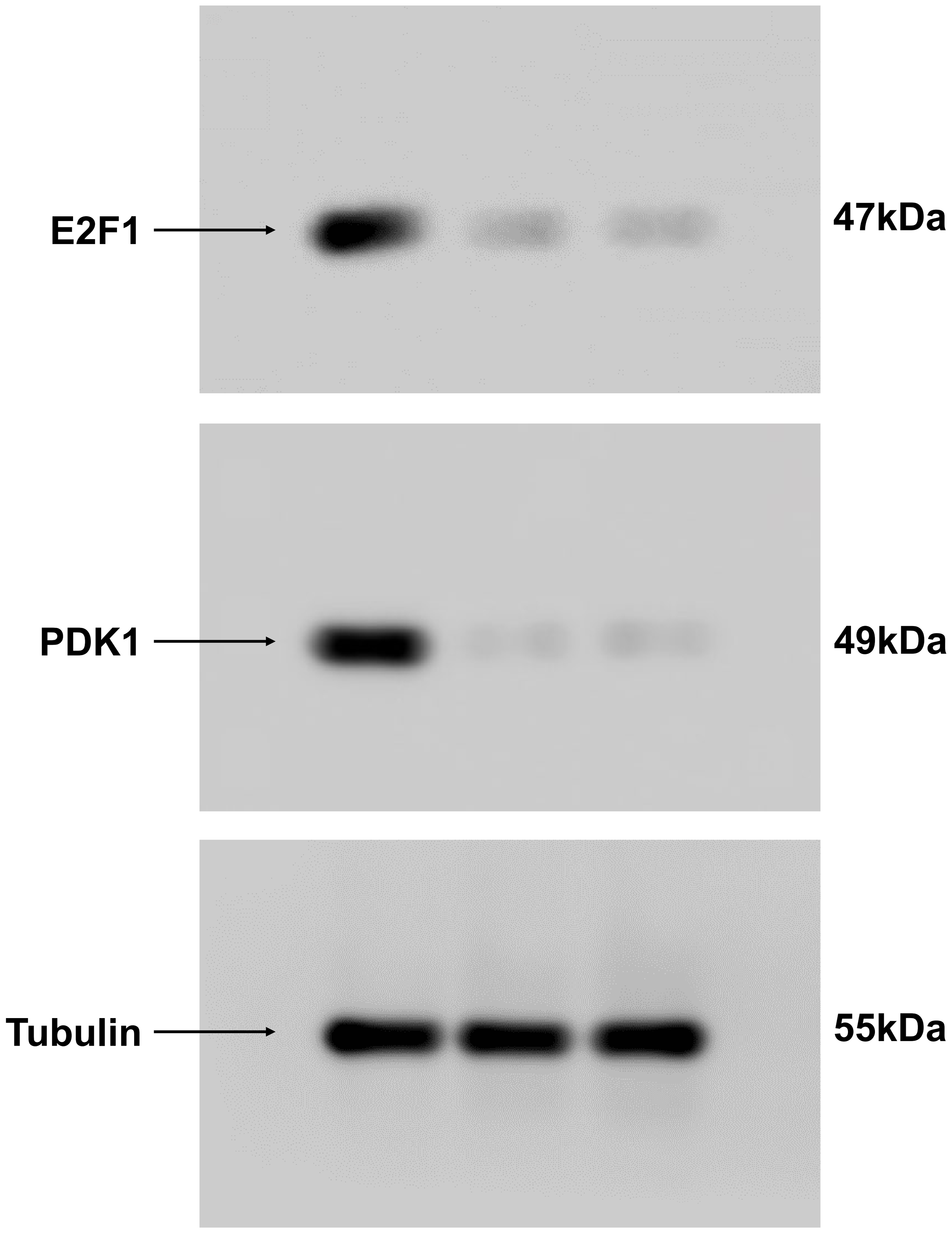

Figure 5G

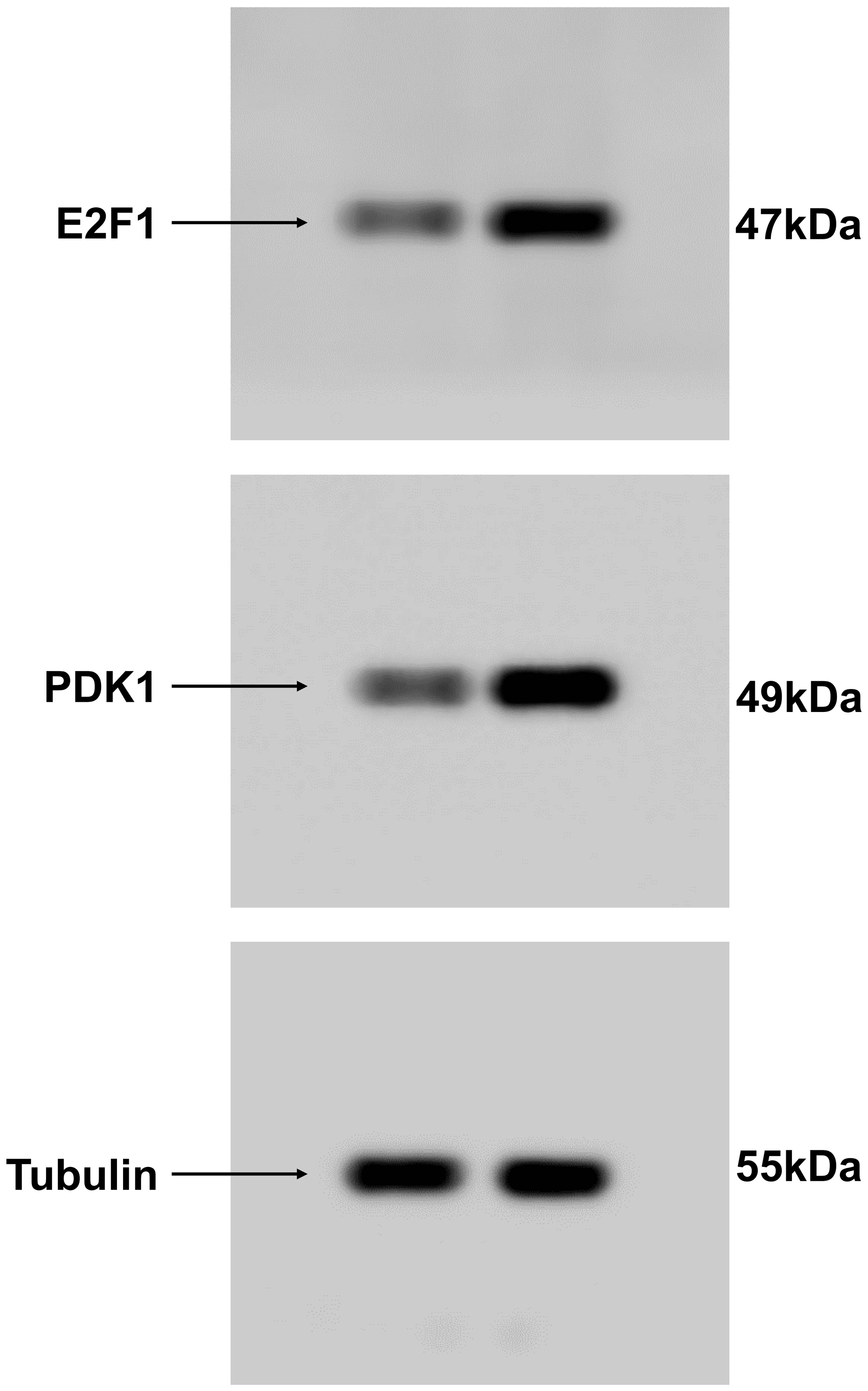

Full and uncropped western blot for Figure 6

Figure 6A (left)

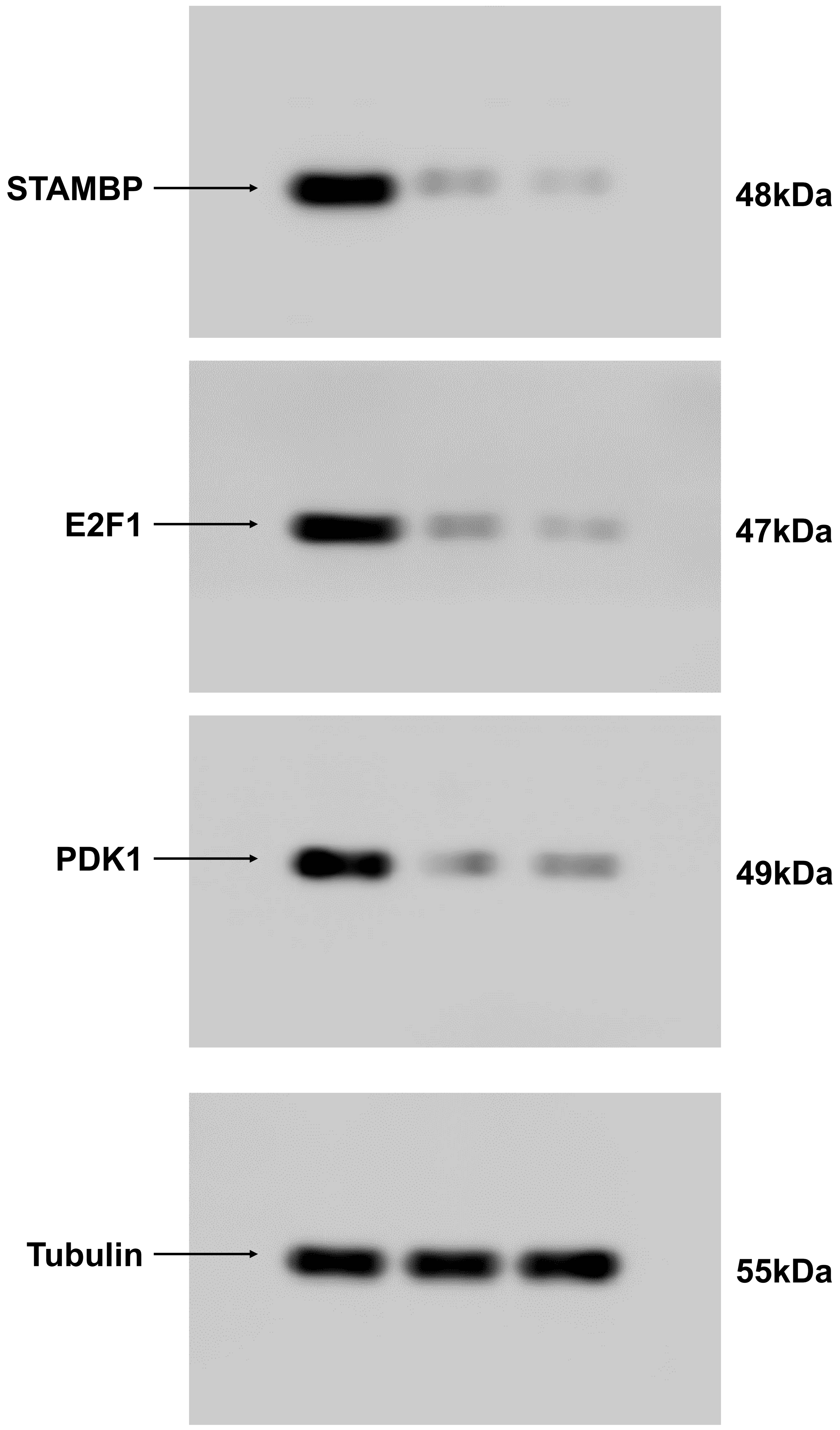

Figure 6A (right)

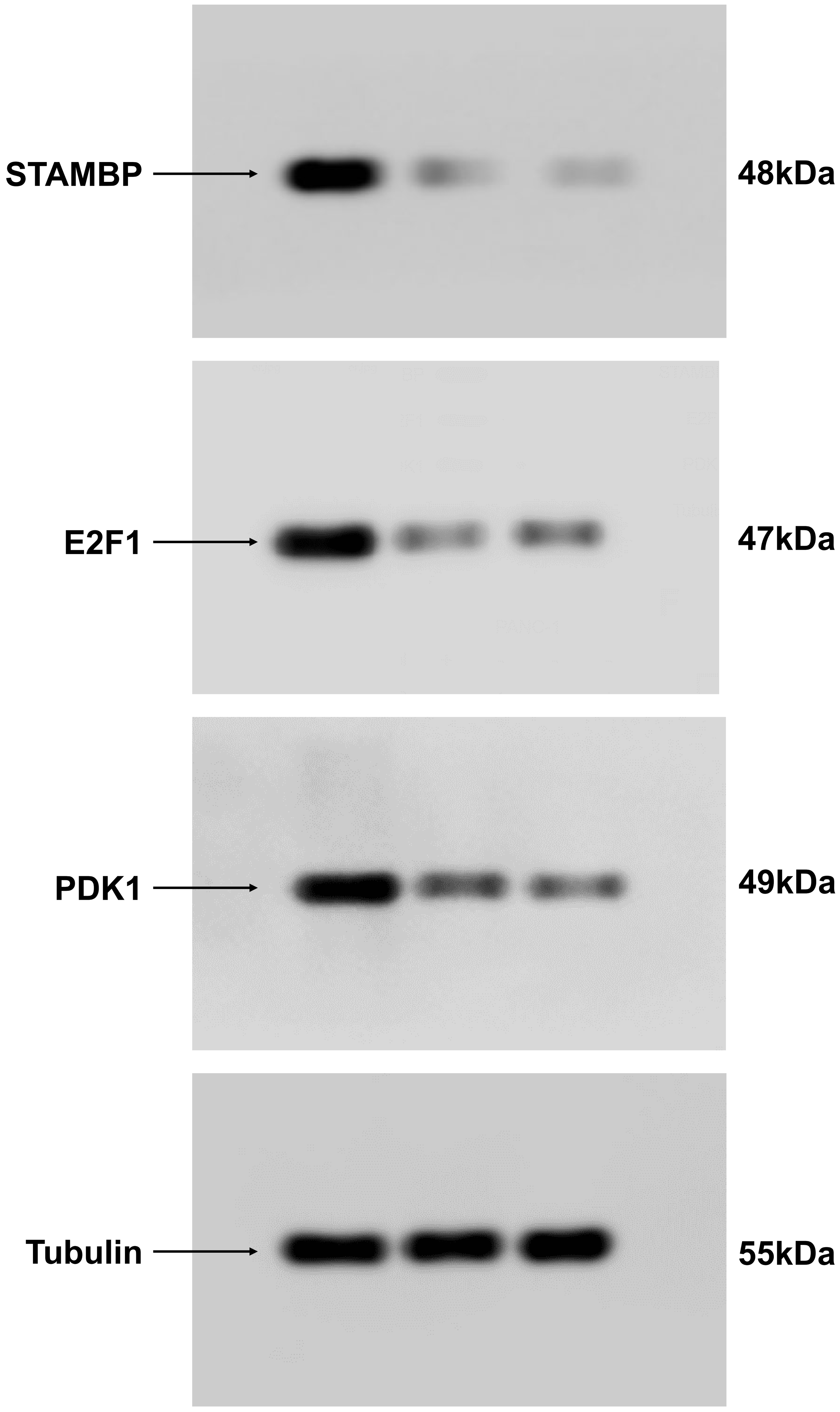

Full and uncropped western blot for Figure 6

Figure 6B (left)

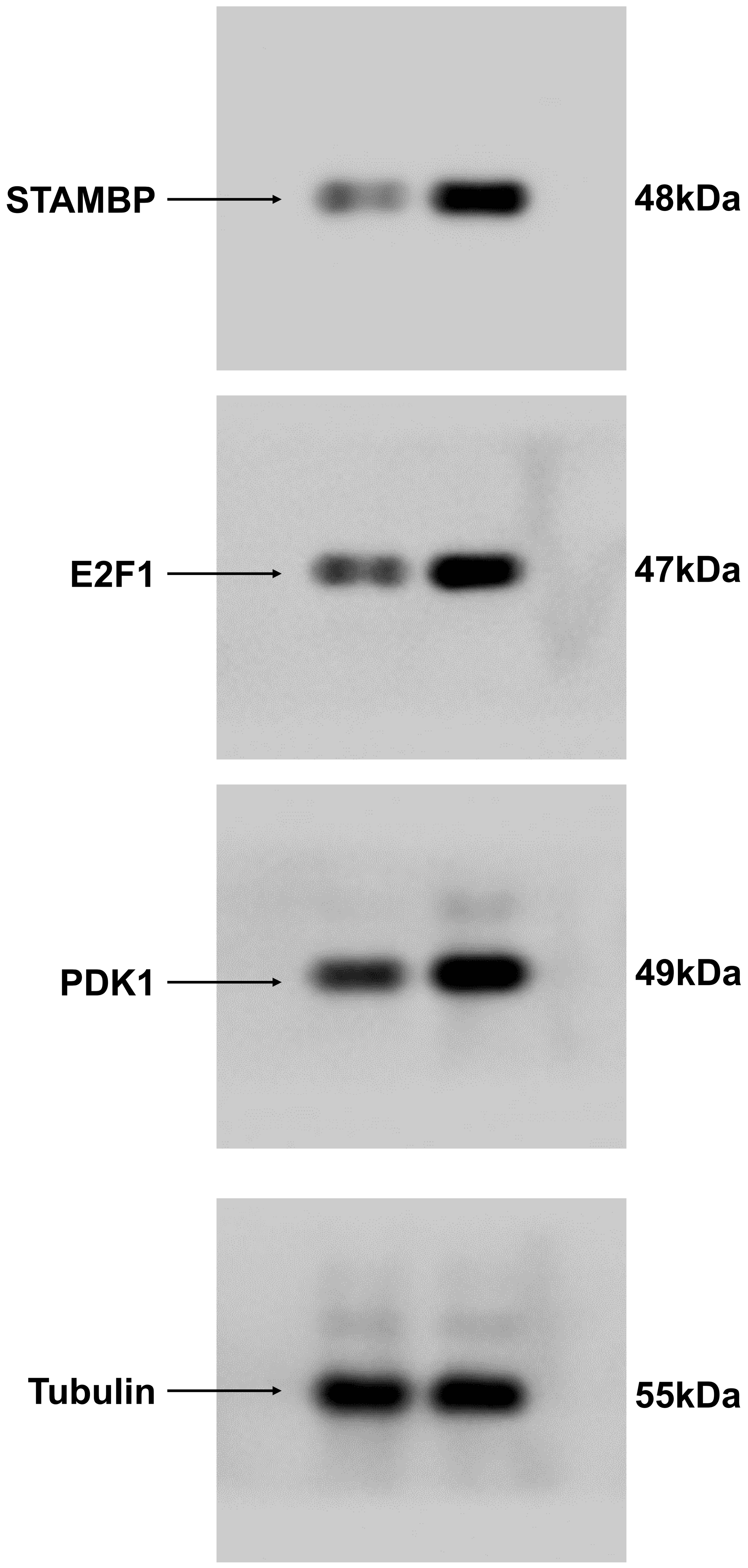

Figure 6B (right)

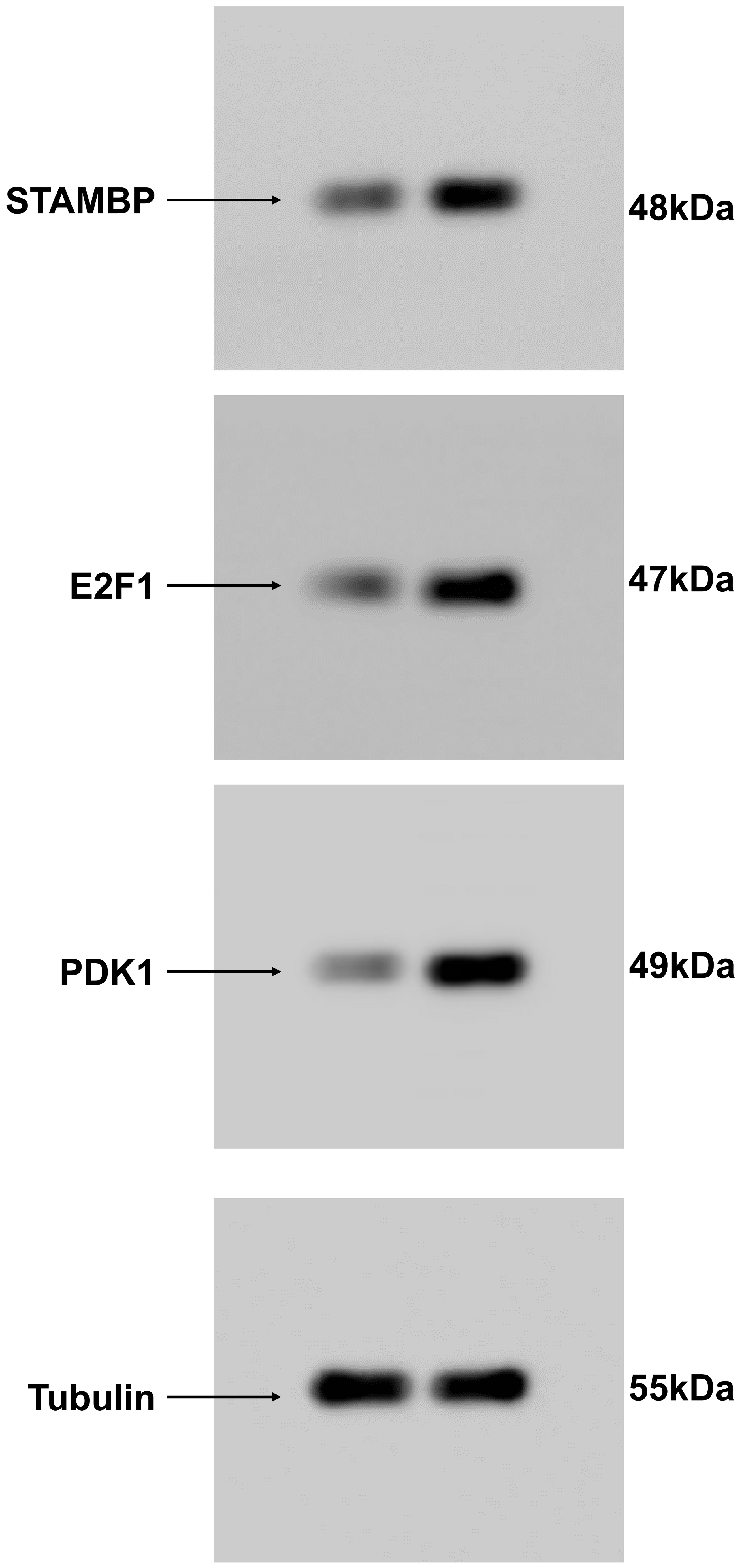

Full and uncropped western blot for Figure 6

Figure 6E

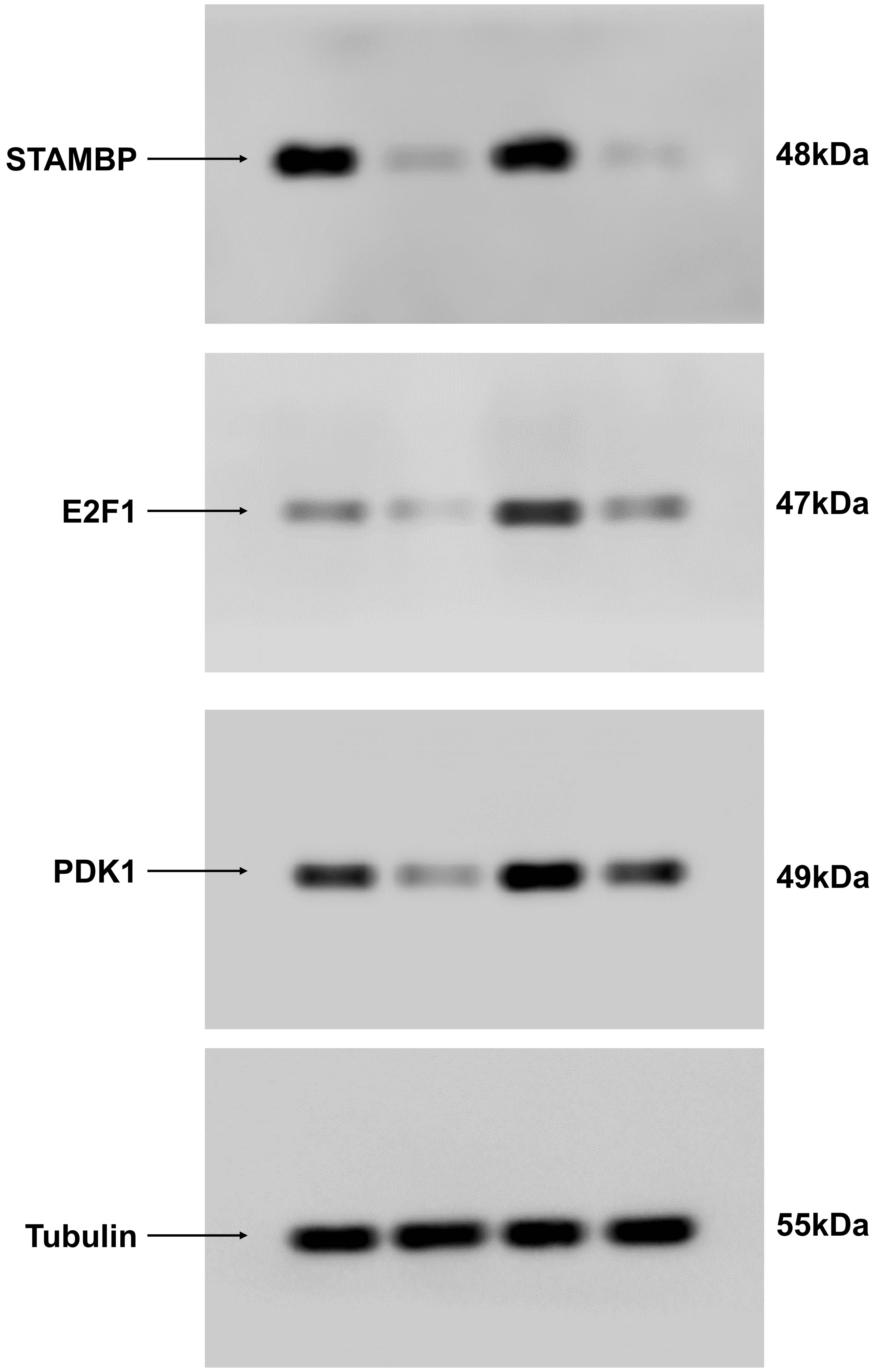

Figure 6K

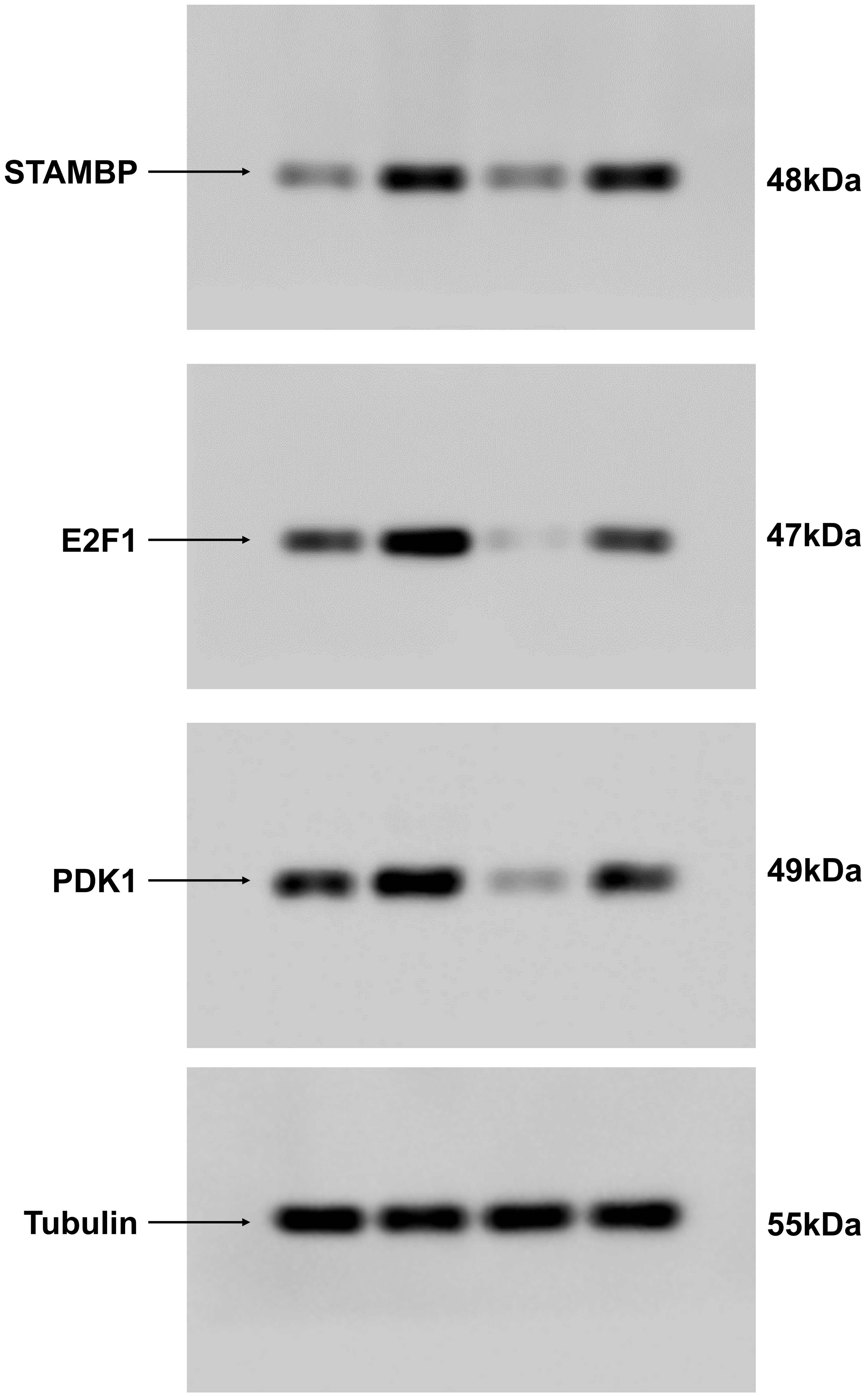

Full and uncropped western blot for Figure 7

Figure 7A (left)

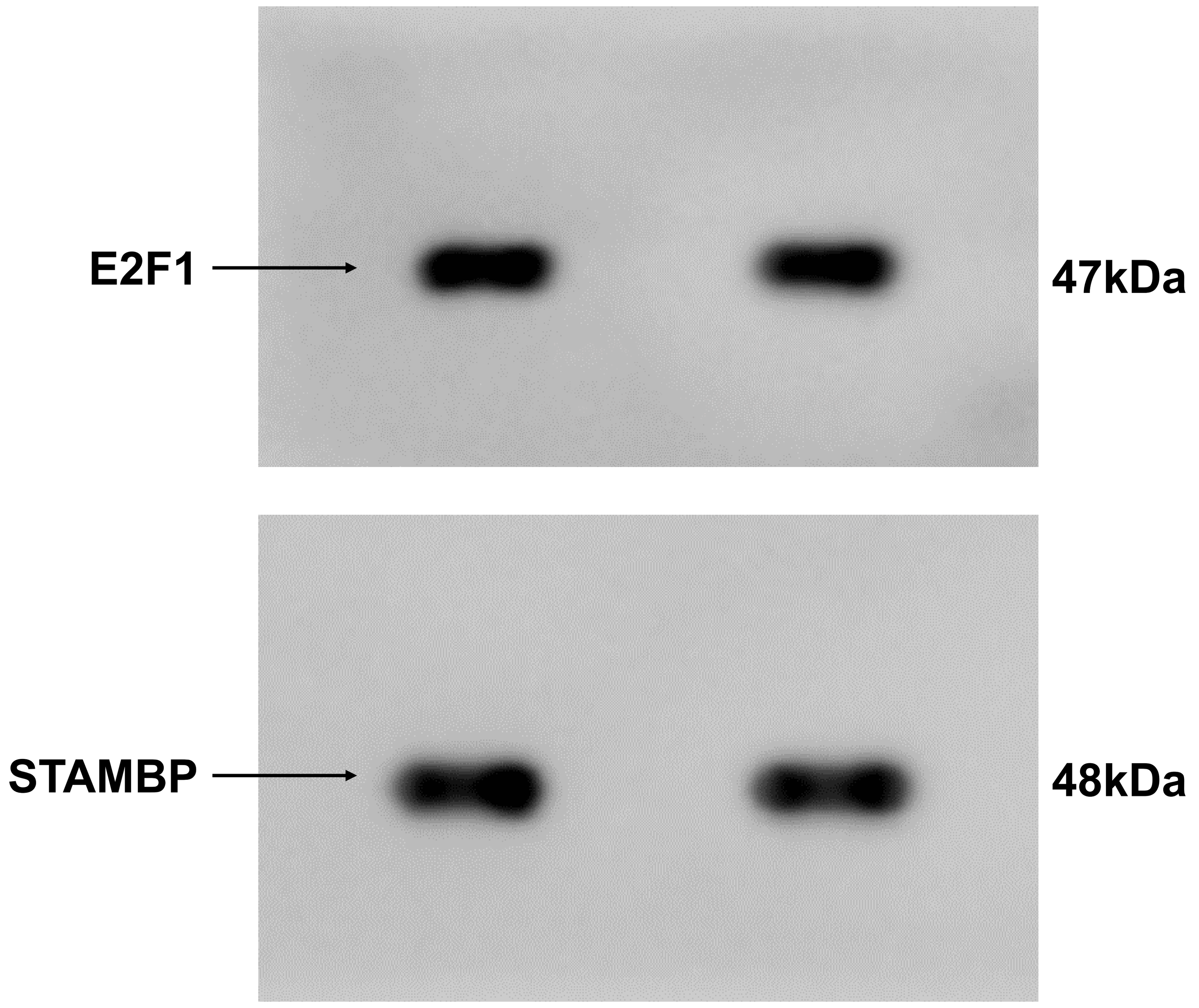

Figure 7A (right)

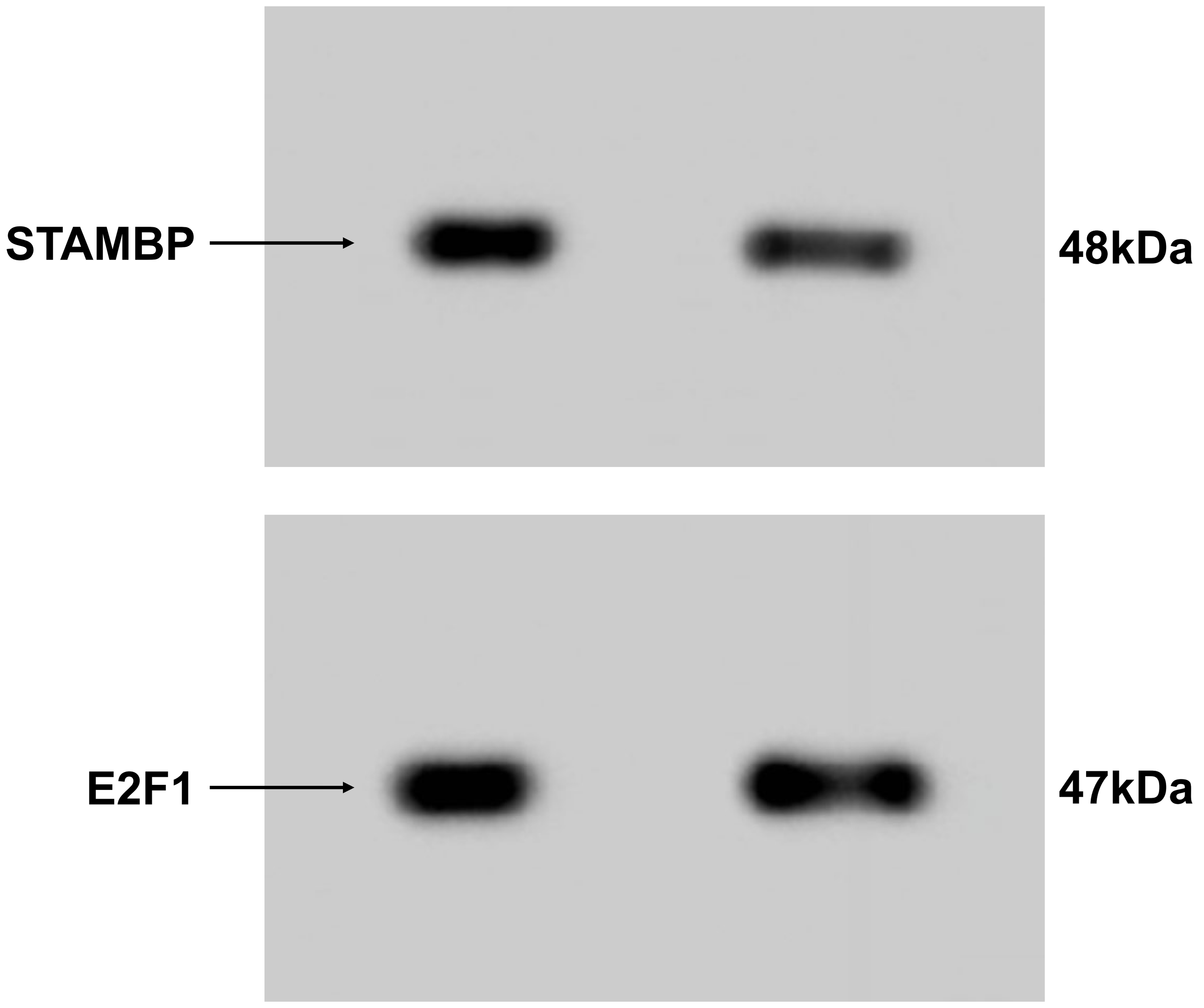

Figure 7B (left)

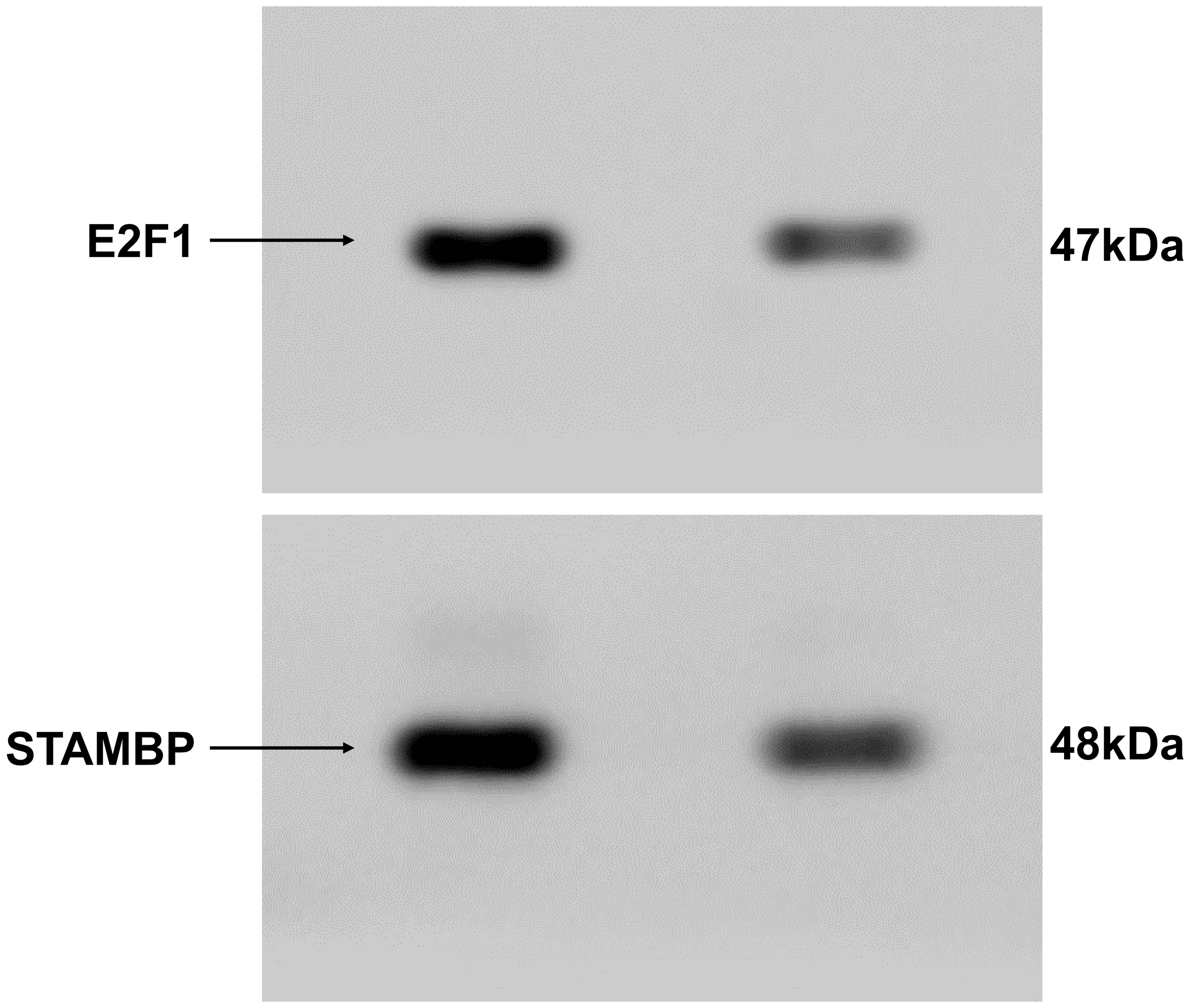

Figure 7A (right)

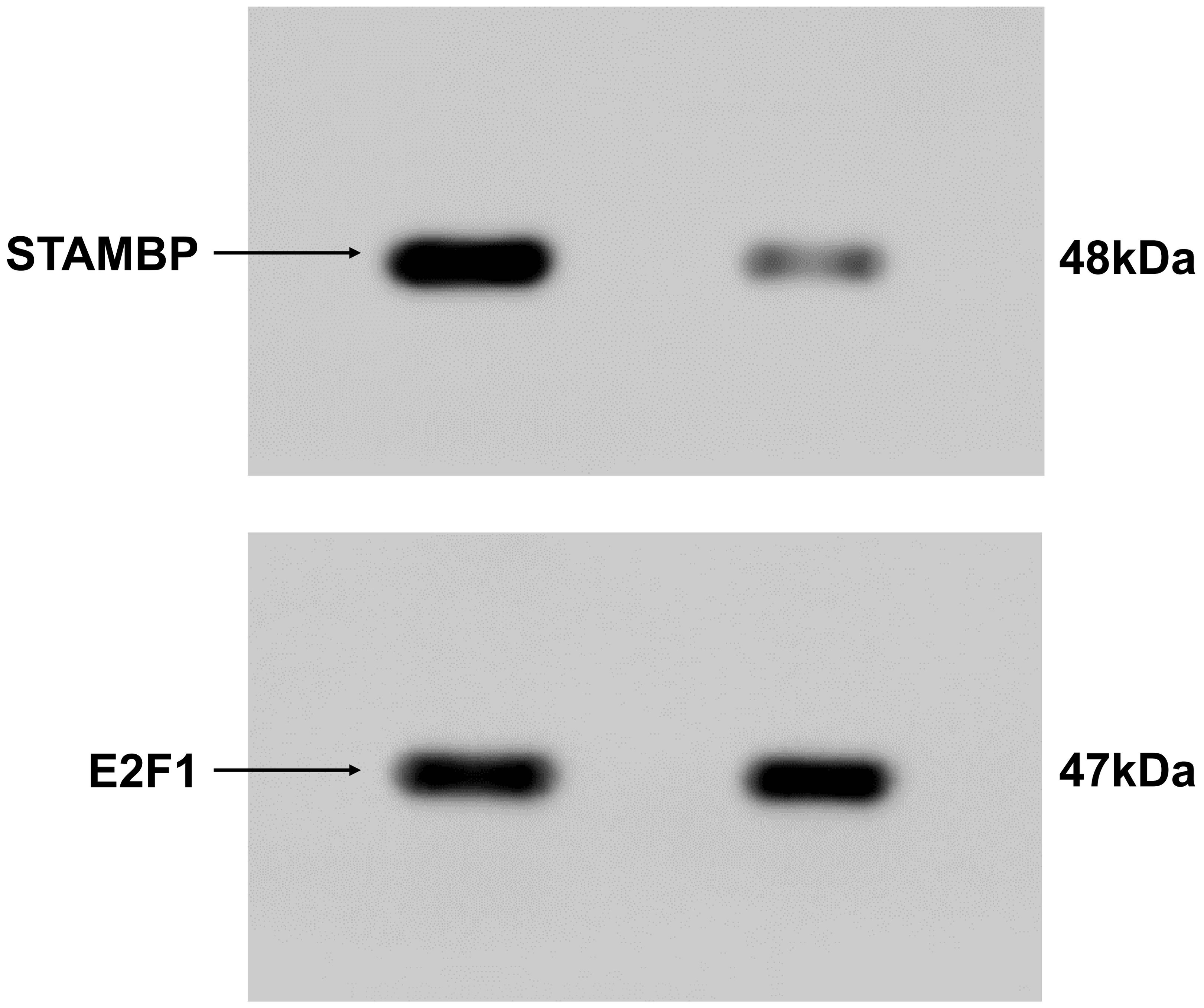

Full and uncropped western blot for Figure 7

Figure 7E

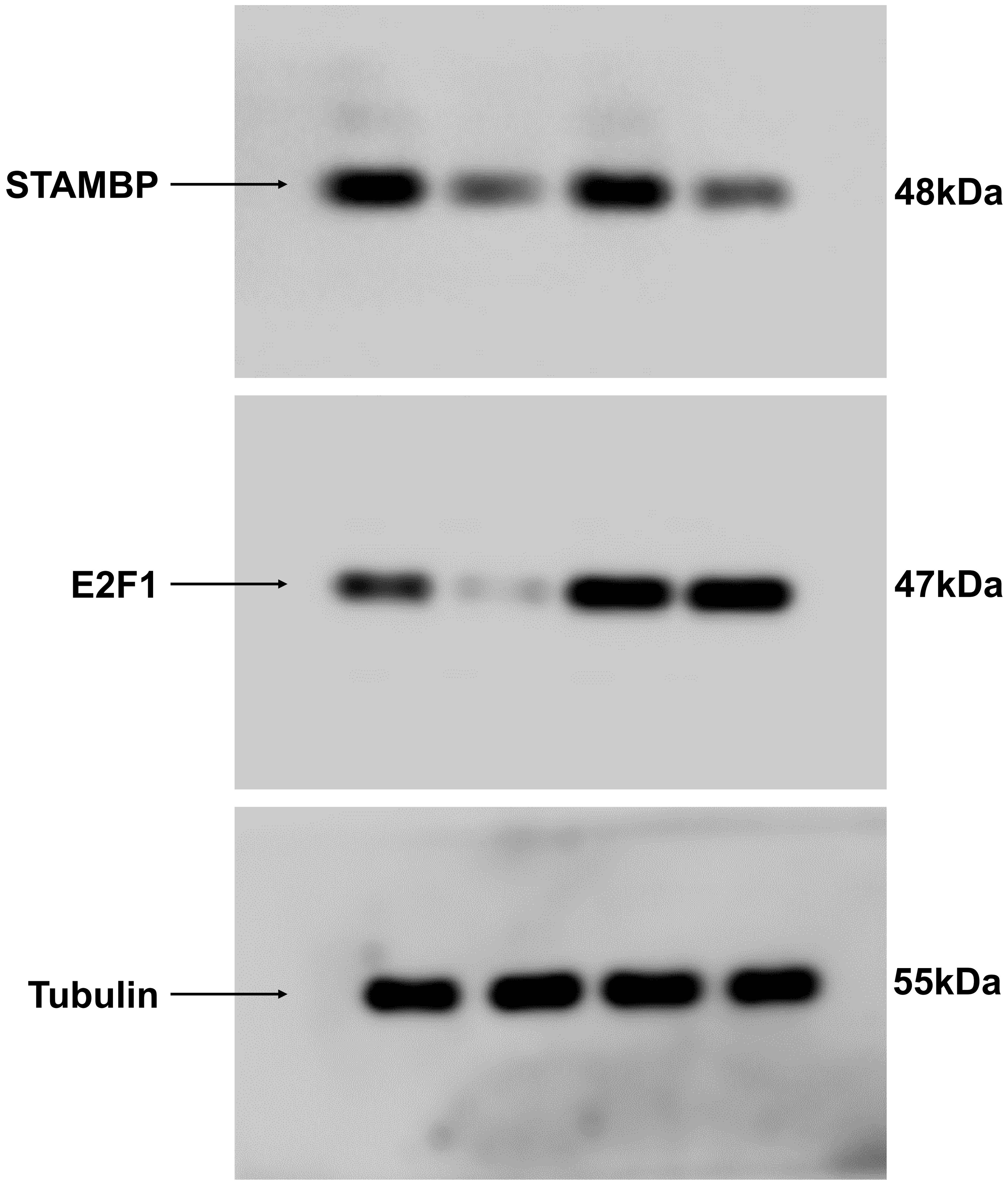

Figure 7F

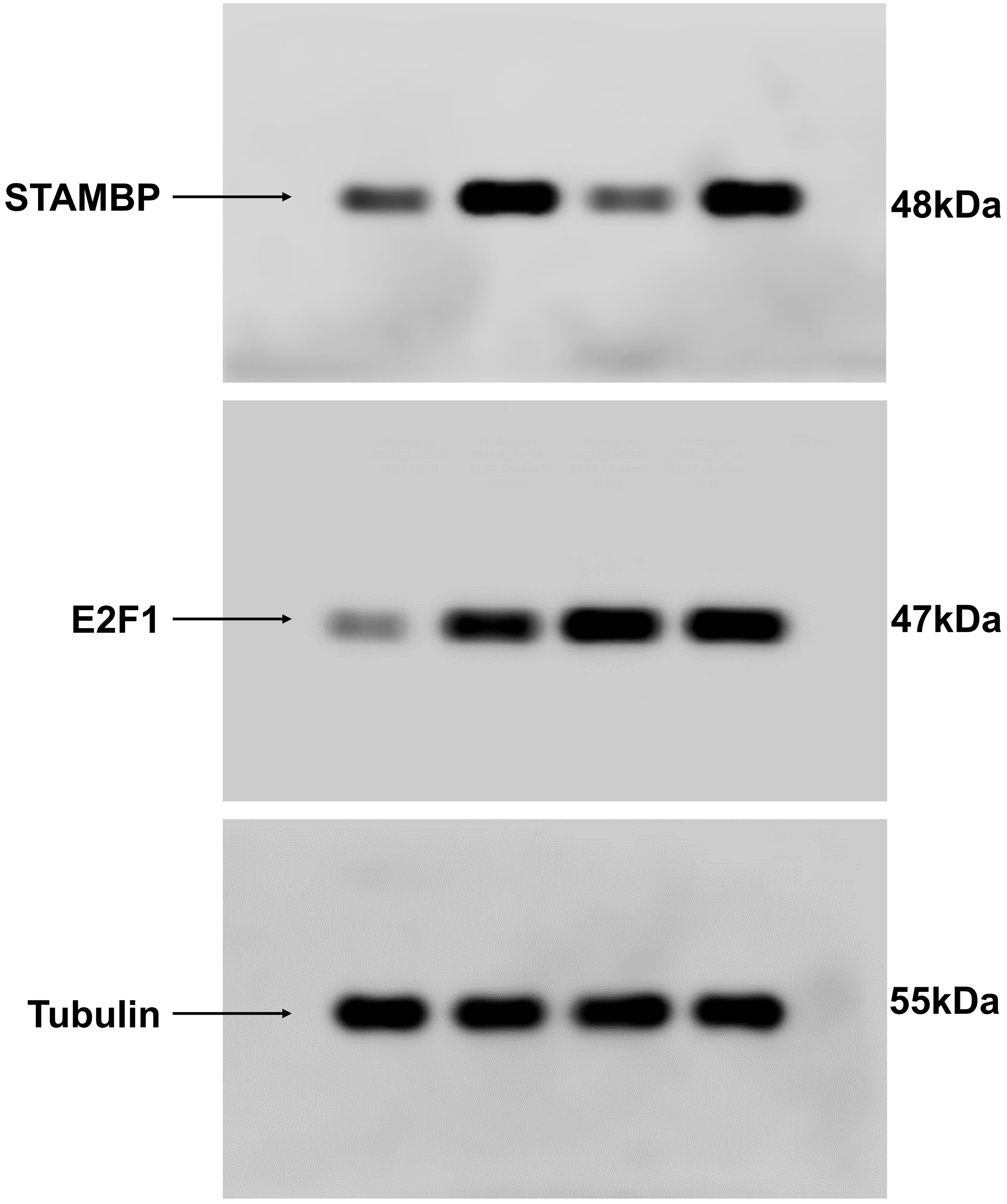

Full and uncropped western blot for Figure 7

Figure 7G

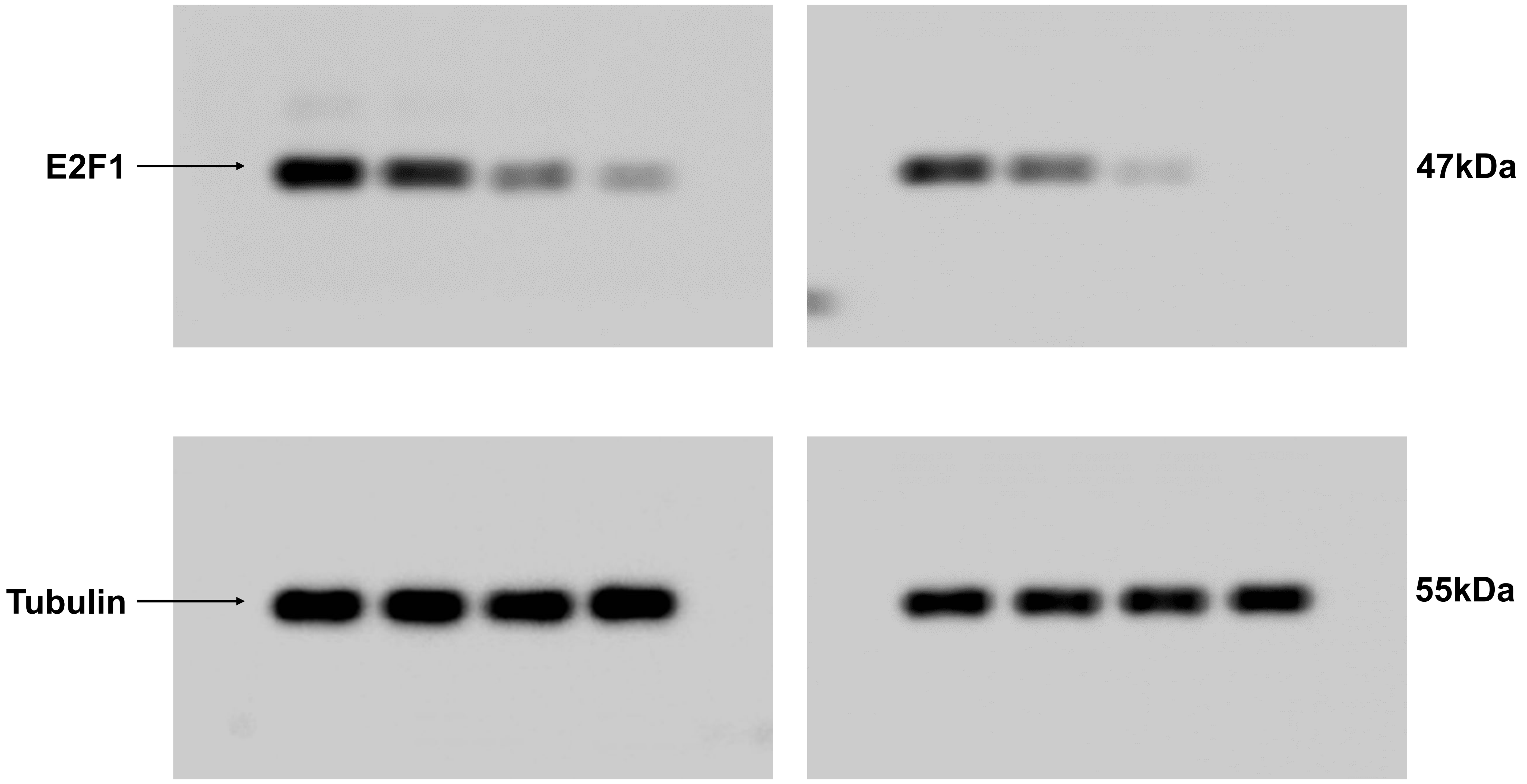

Figure 7H

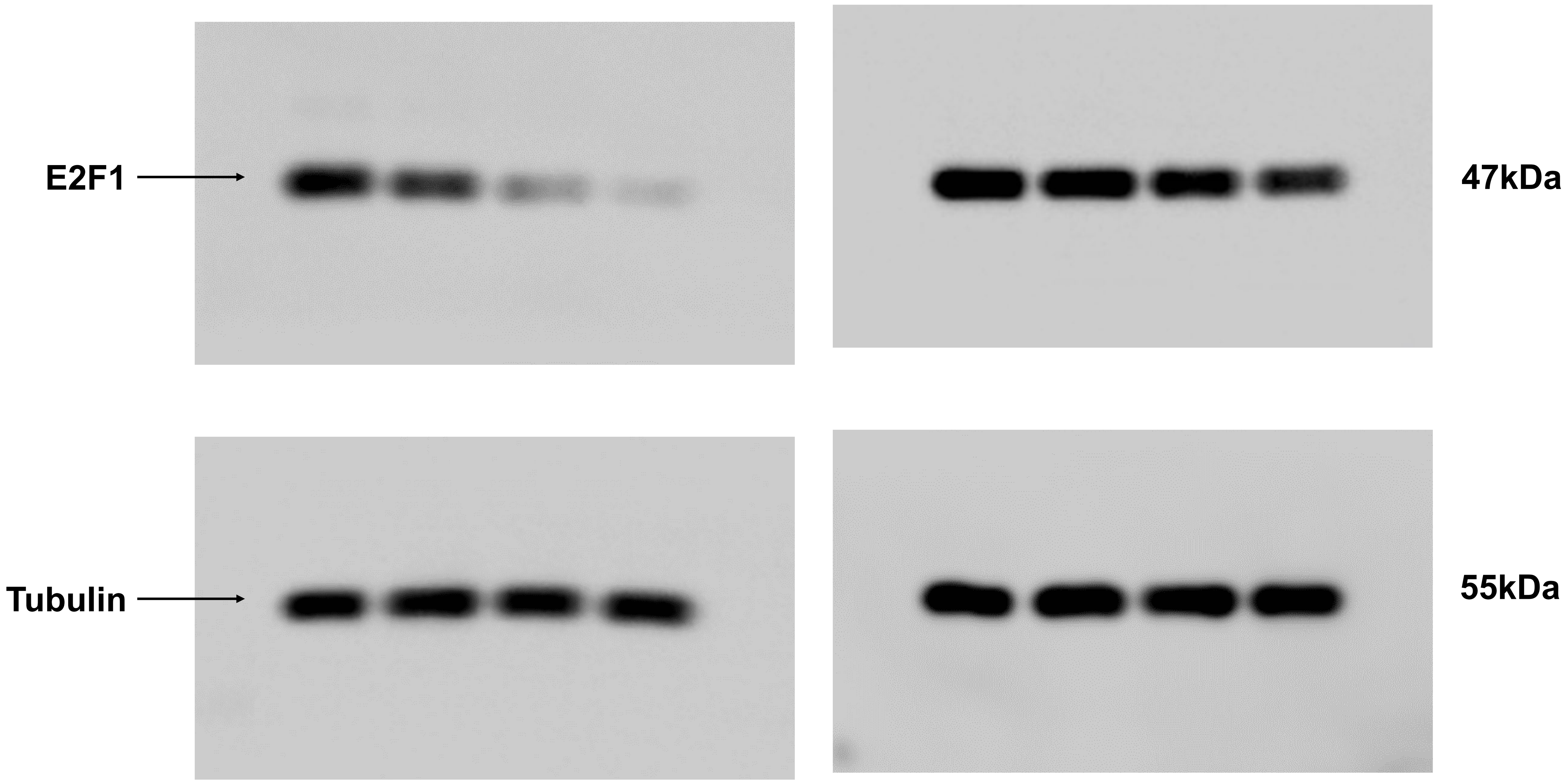

Figure 7I (left)

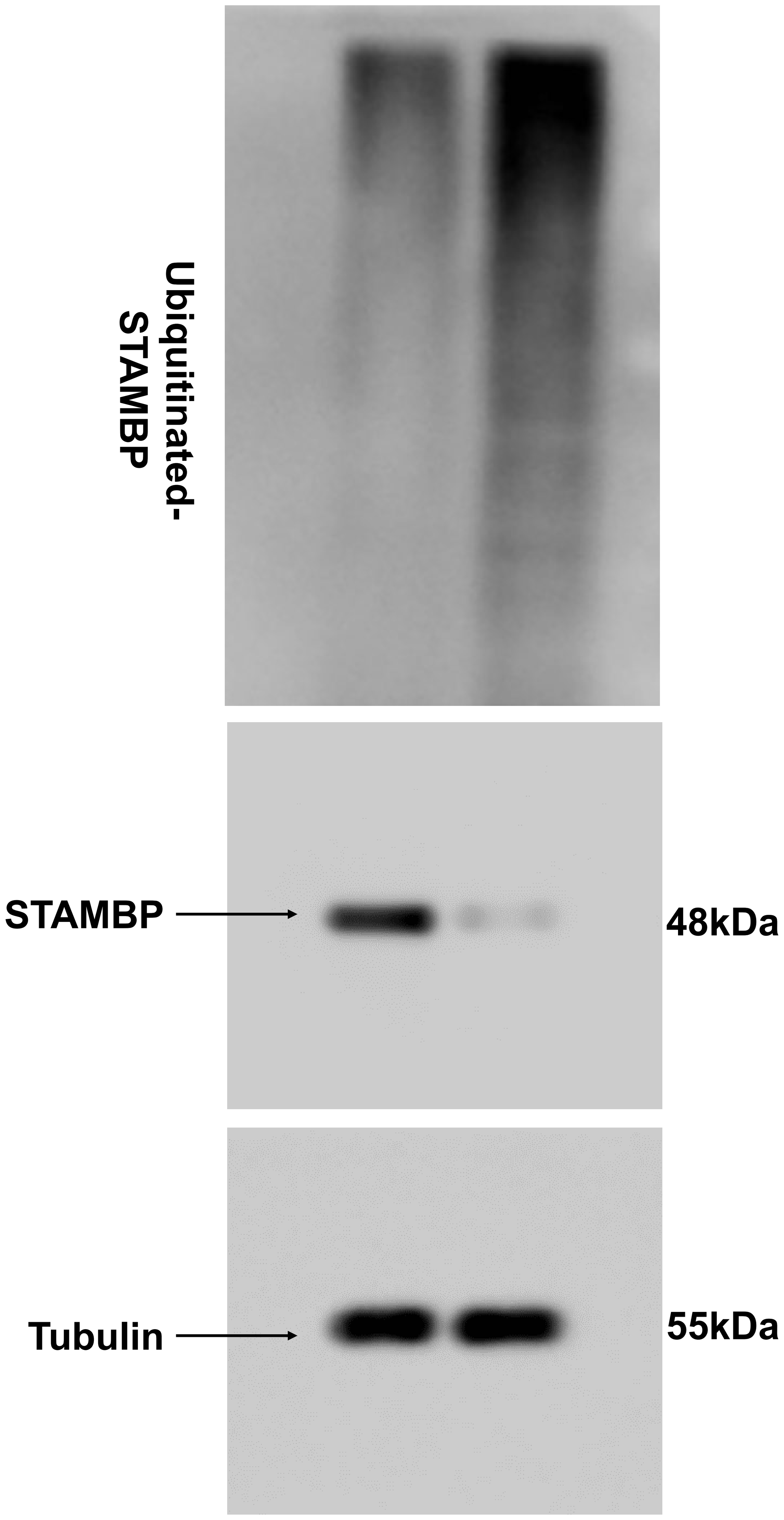

Full and uncropped western blot for Figure 7

Figure 7I (right)

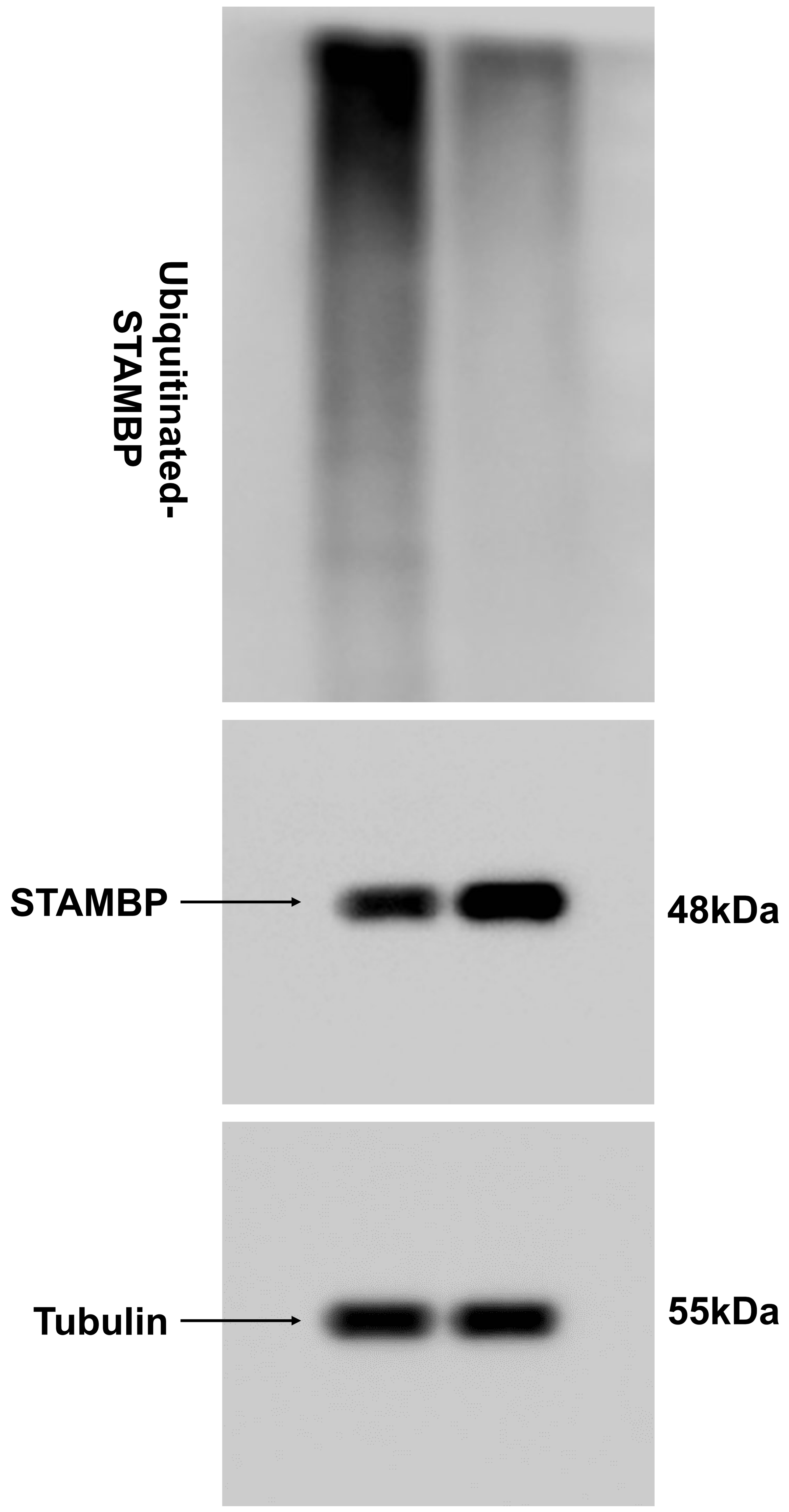

Figure 7J (left)

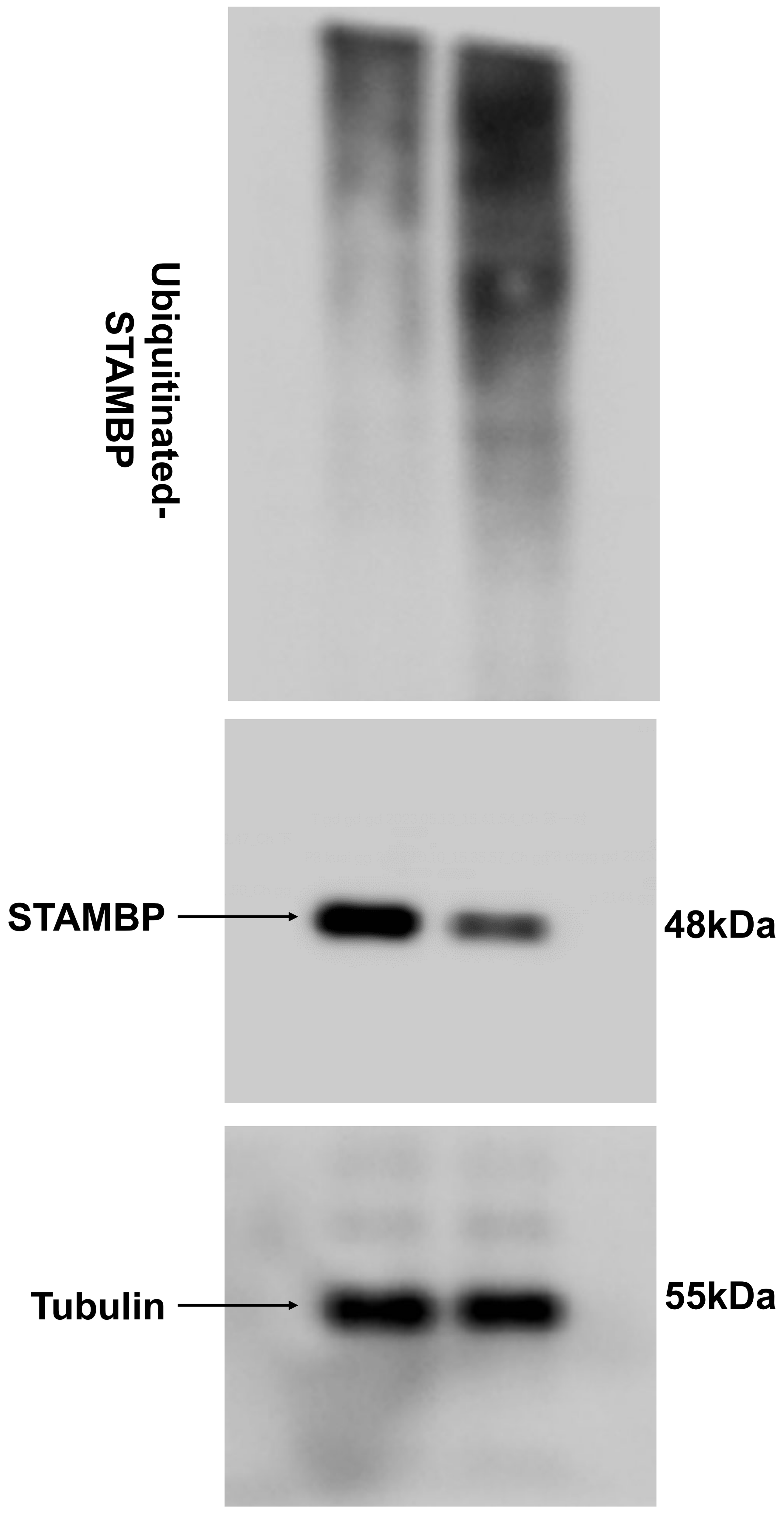

Figure 7J (right)

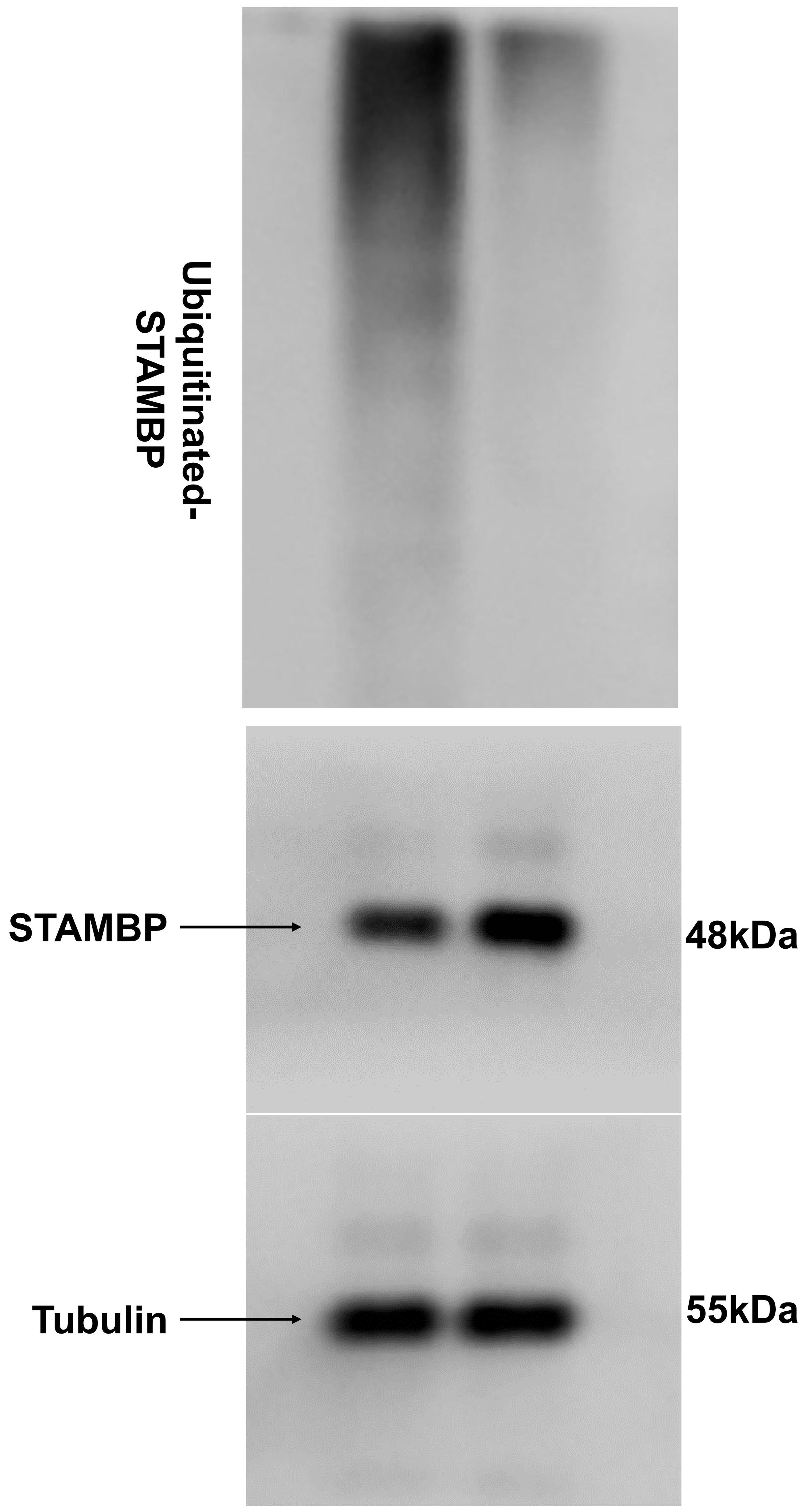

Full and uncropped western blot for Figure 8

Figure 8B (left)

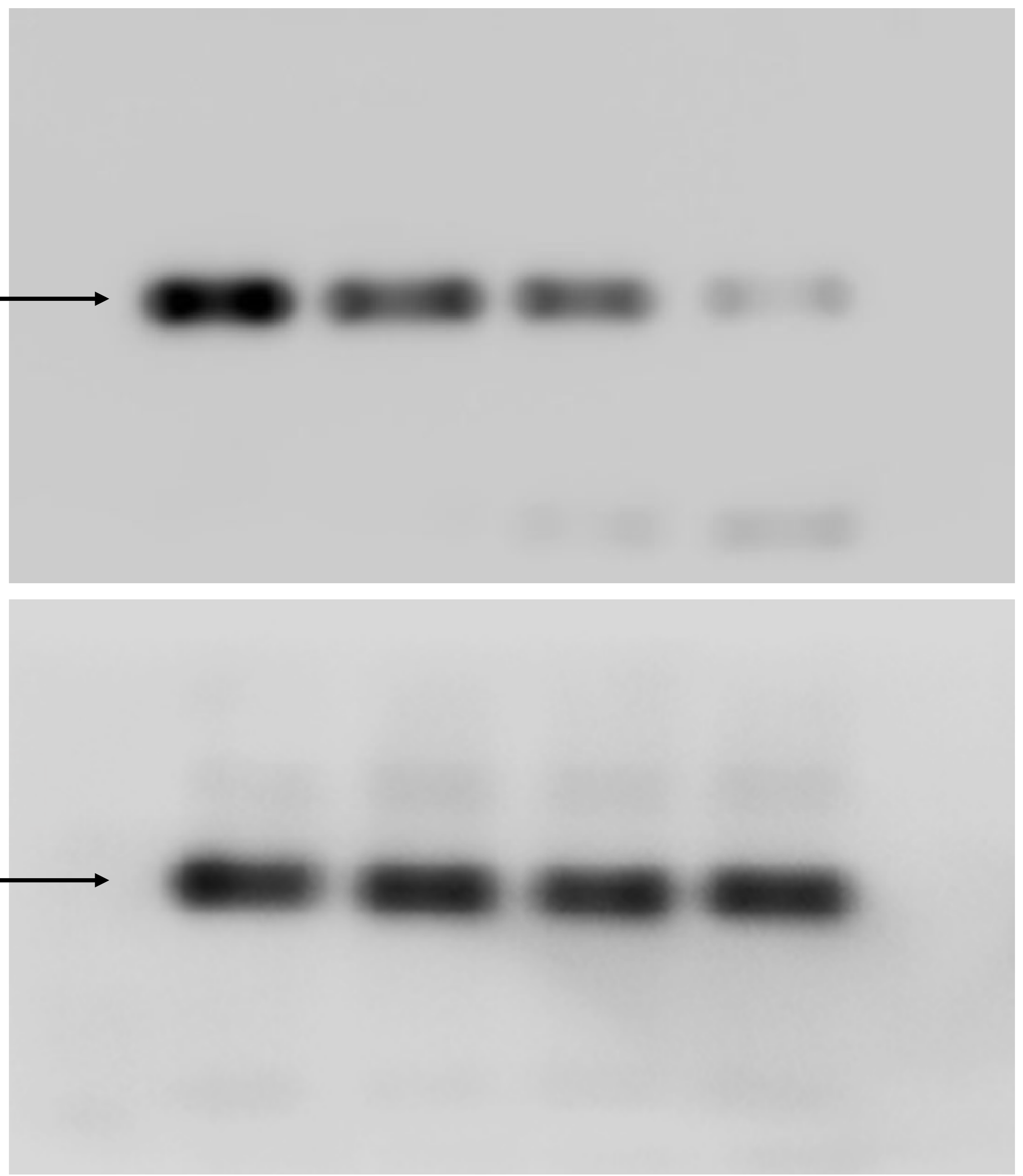

STAMBP → 48kDa

Tubulin → 55kDa

Figure 8B (right)

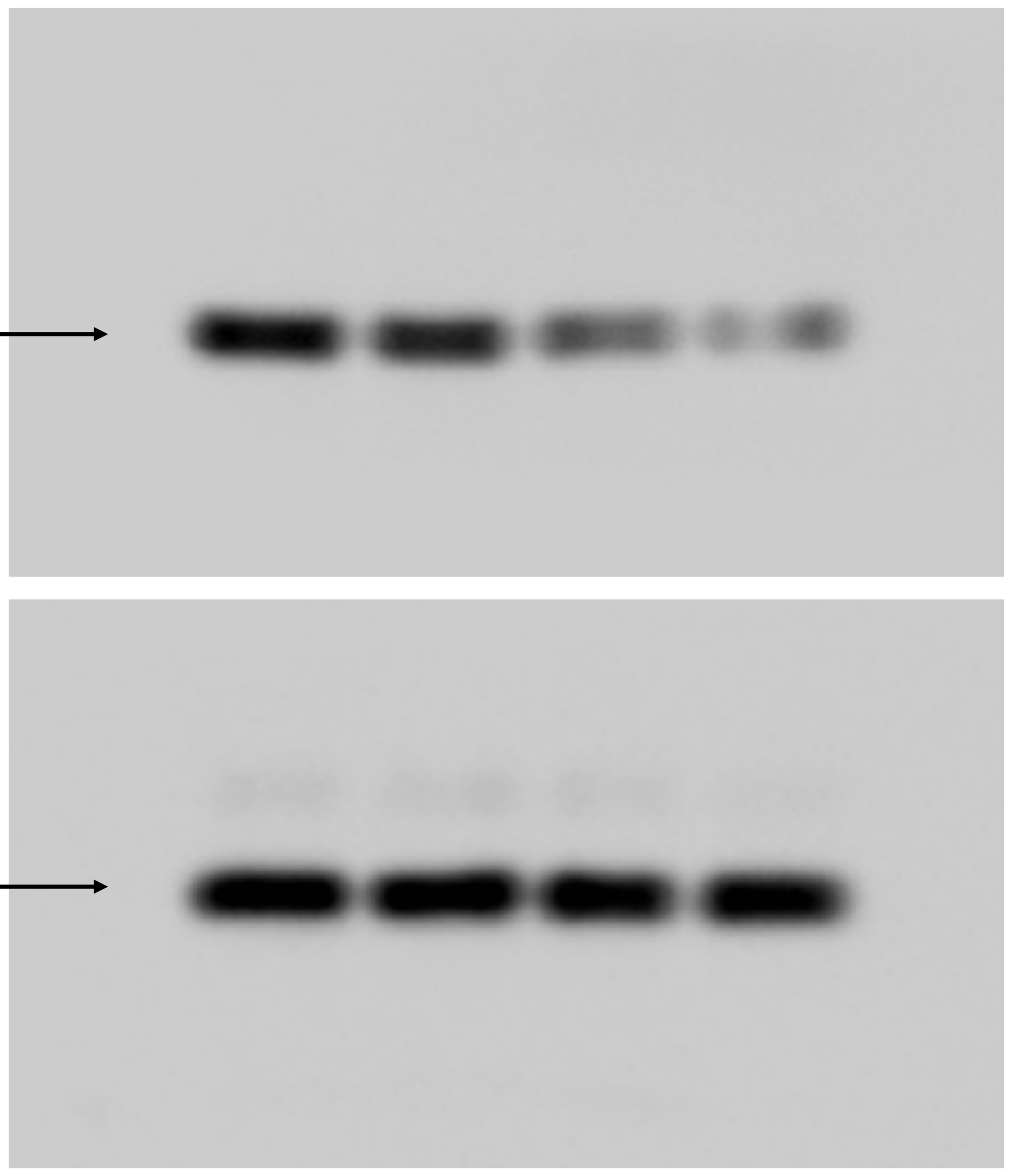

STAMBP → 48kDa

Tubulin → 55kDa

Figure 8H

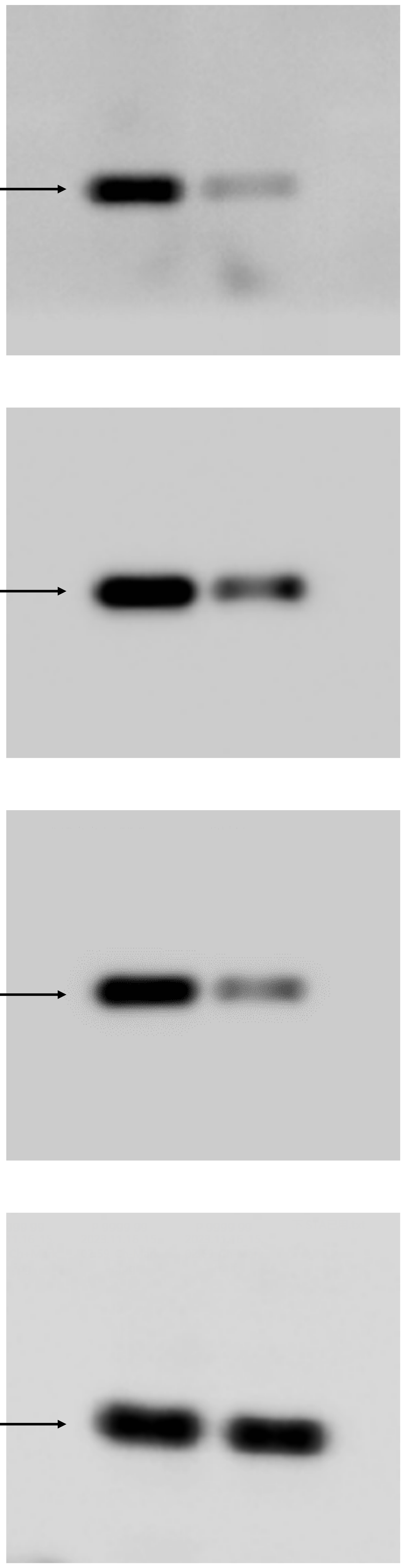

STAMBP → 48kDa

E2F1 → 47kDa

PDK1 → 49kDa

Tubulin → 55kDa

Full and uncropped western blot for Figure 9

Figure 9C

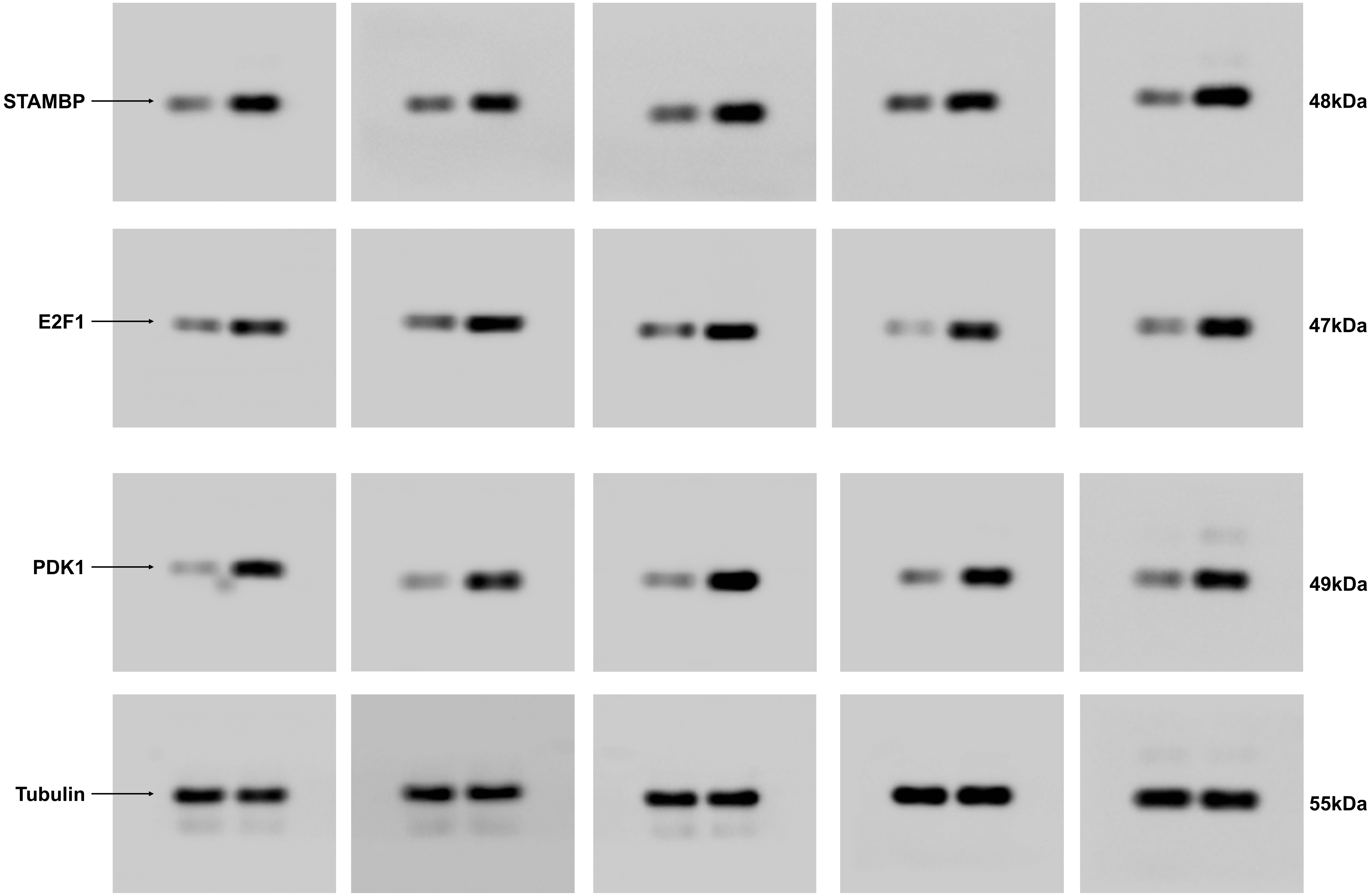

Full and uncropped western blot for Supplementary Figure 2

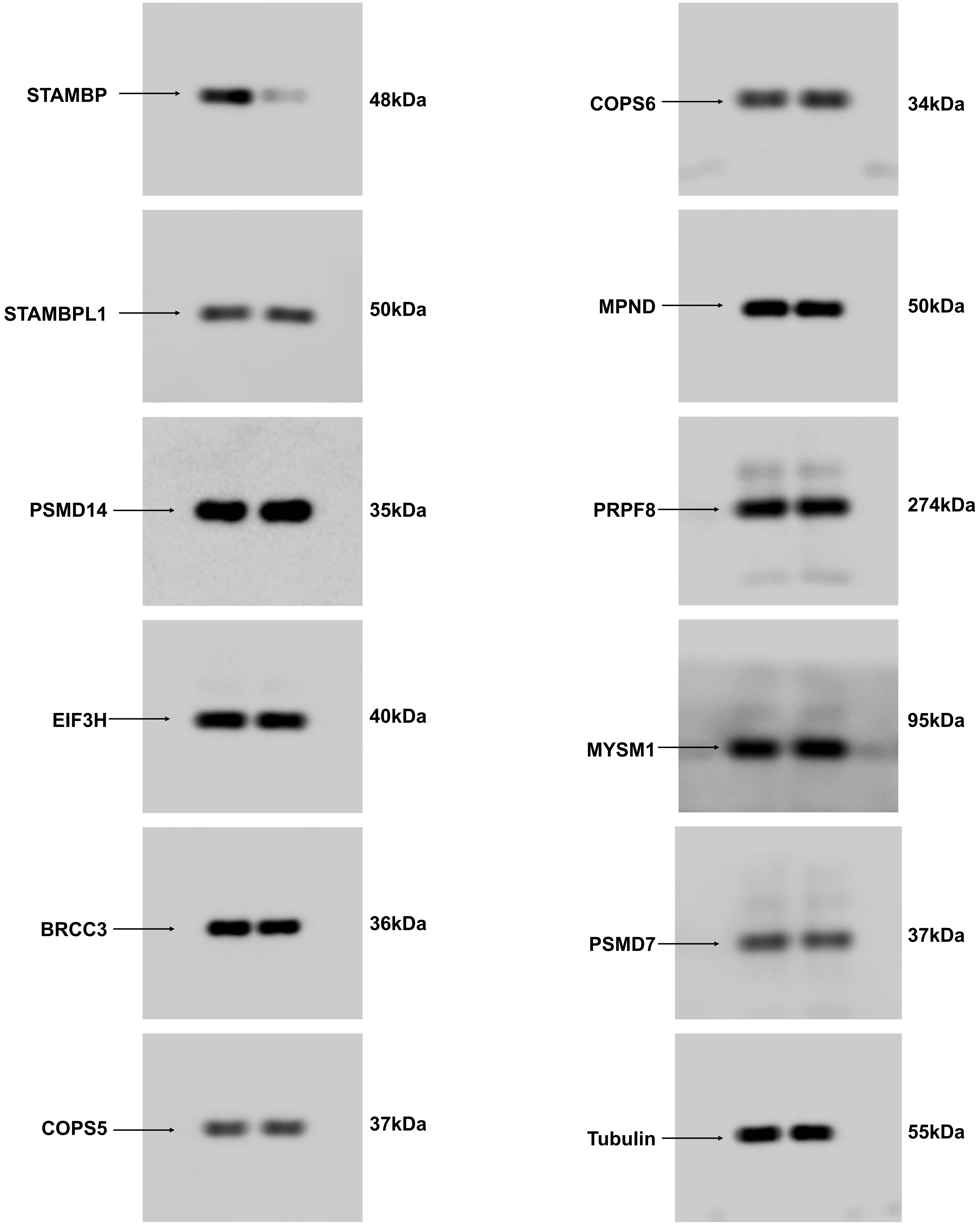

Full and uncropped western blot for Supplementary Figure 3

Figure S3A

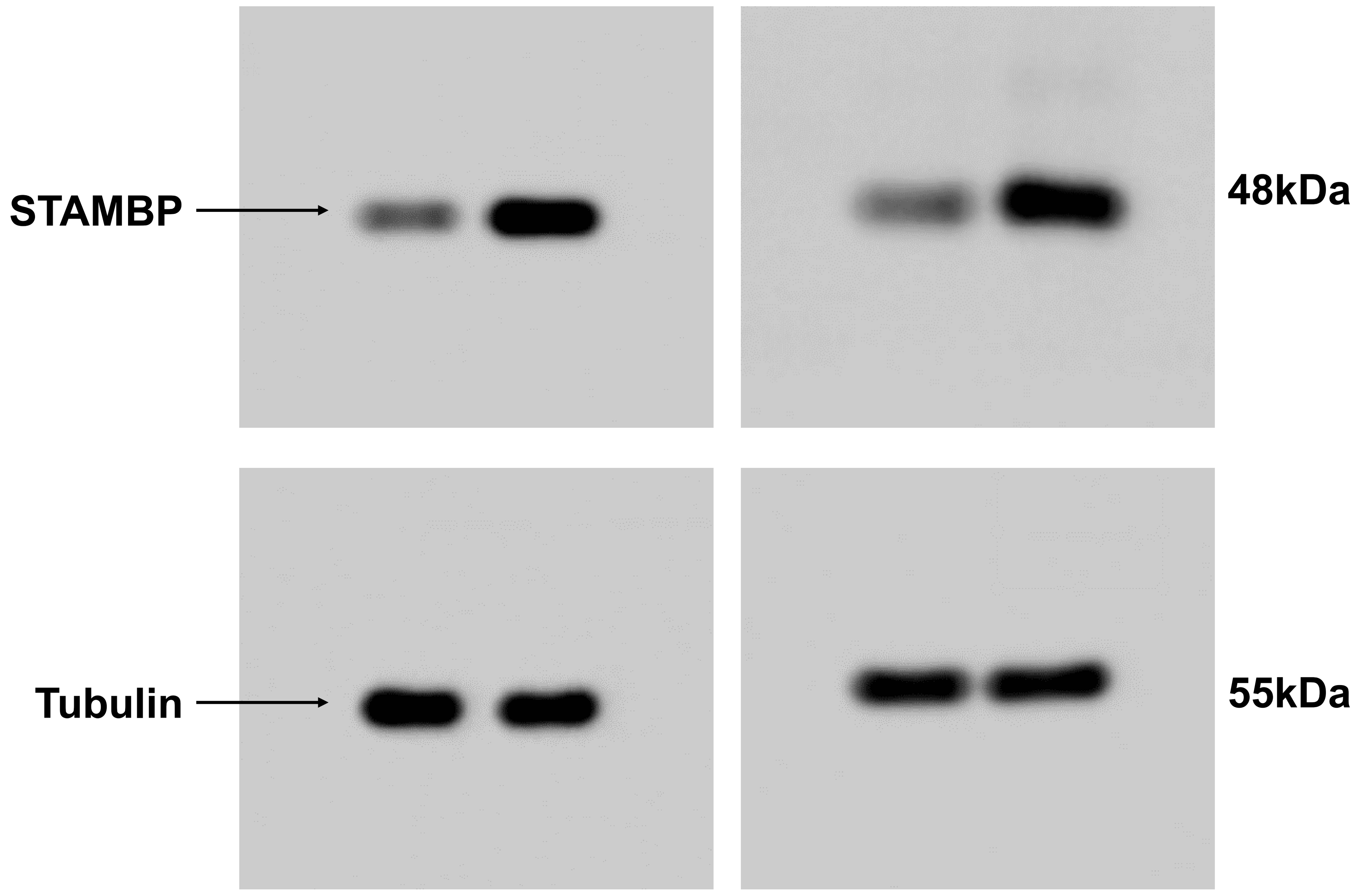

Full and uncropped western blot for Supplementary Figure 5

Figure S5A (top)

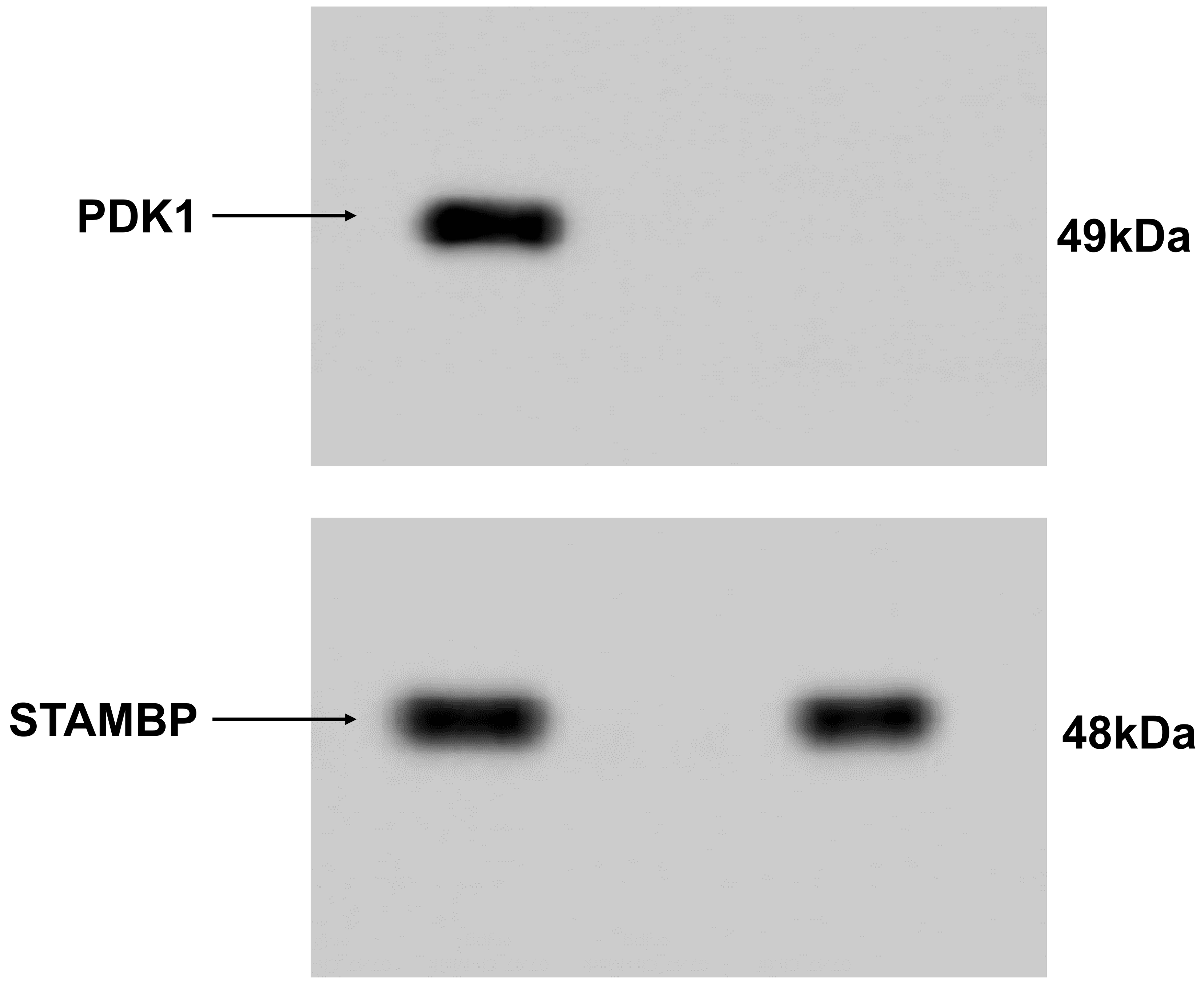

Figure S5A (bottom)

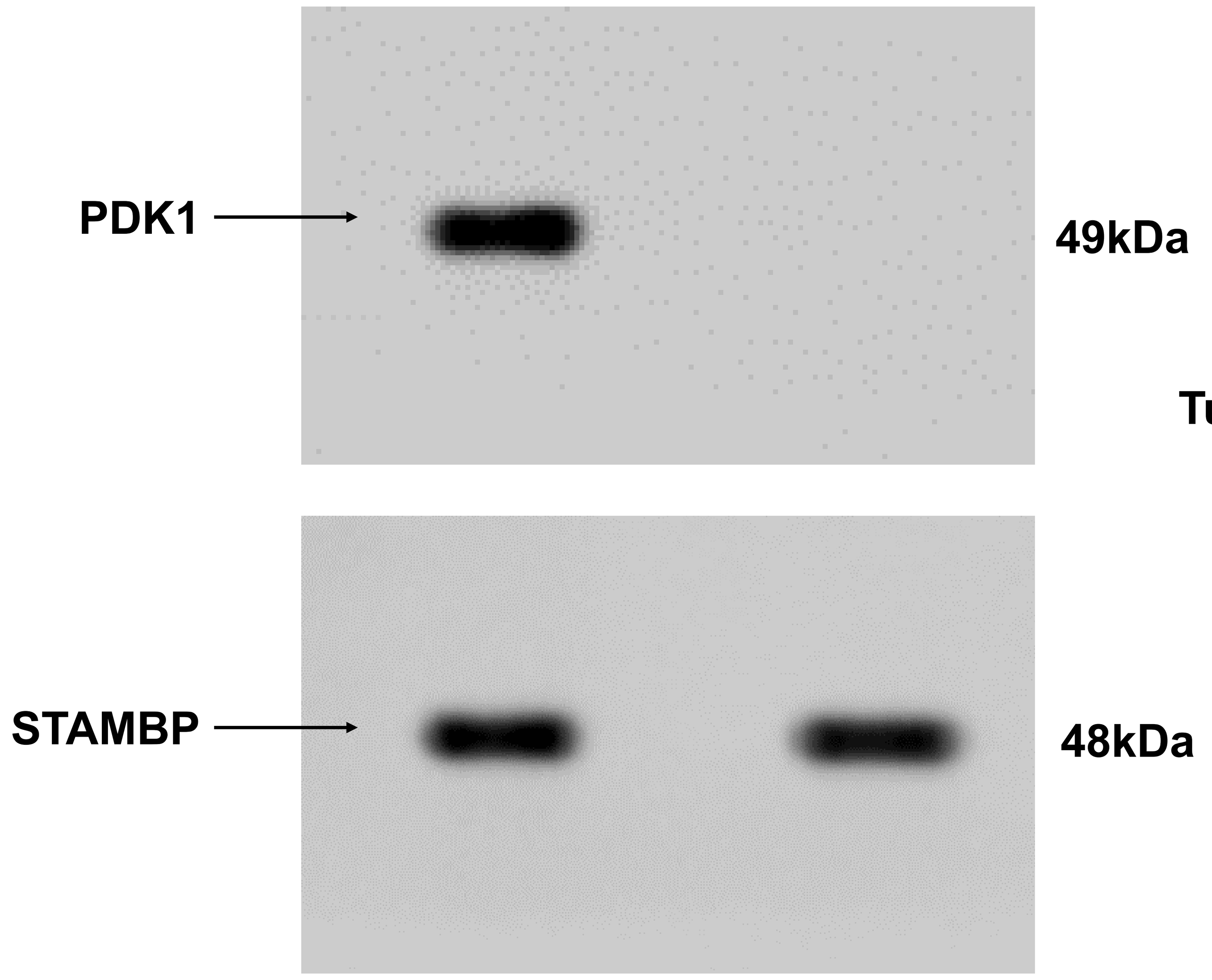

Figure S5C

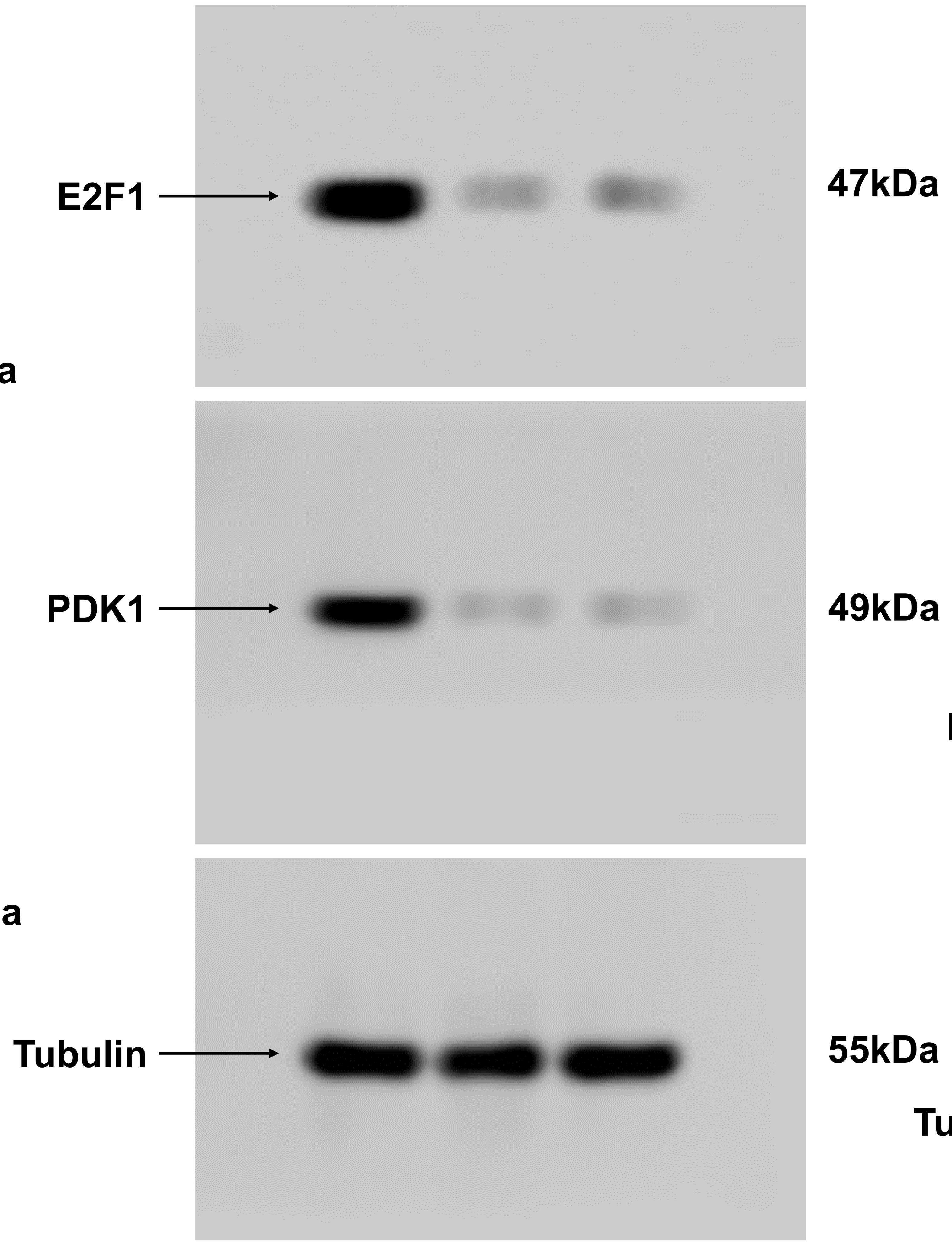

Figure S5E

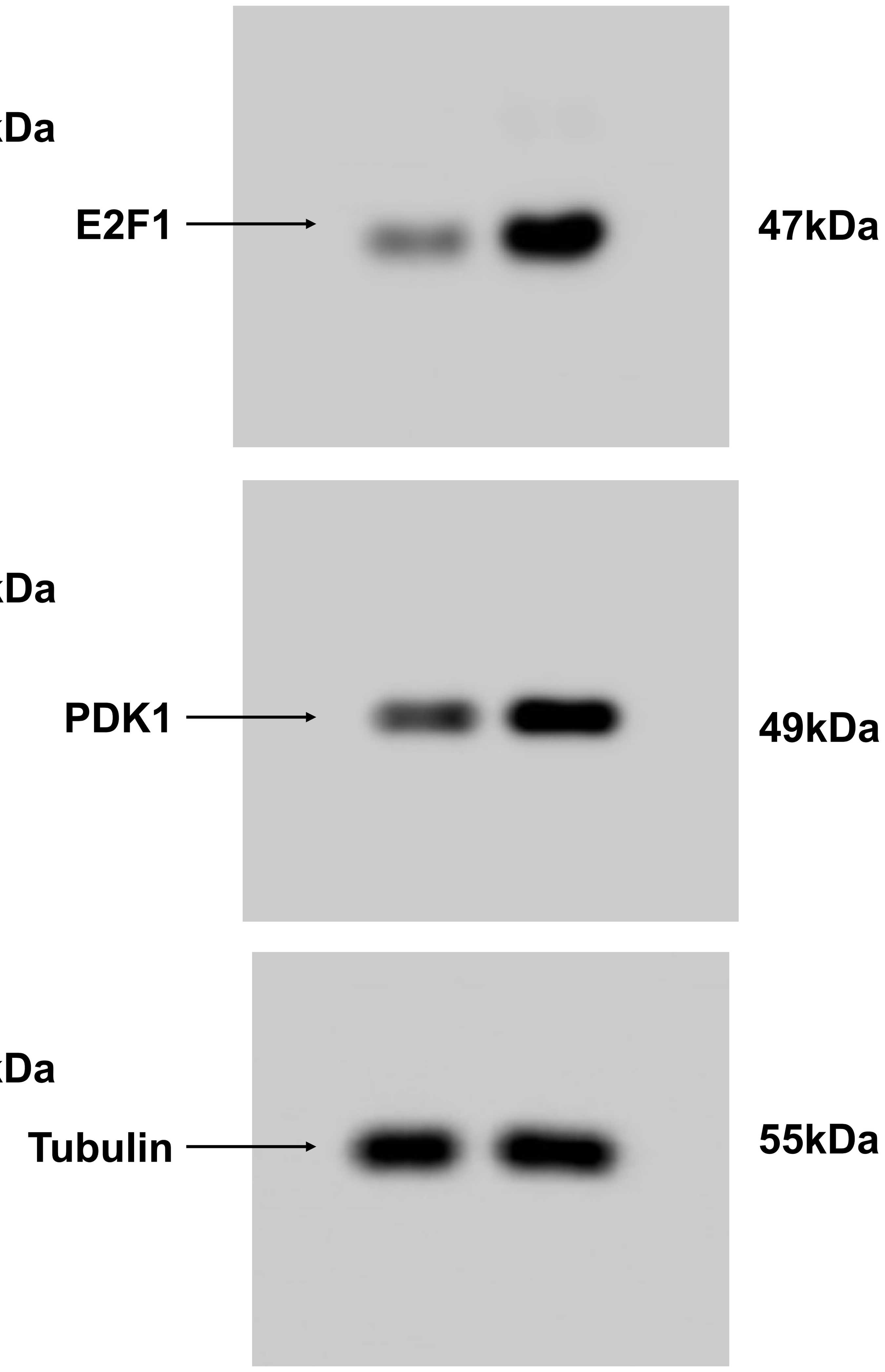

Full and uncropped western blot for Supplementary Figure 7

Figure S7E

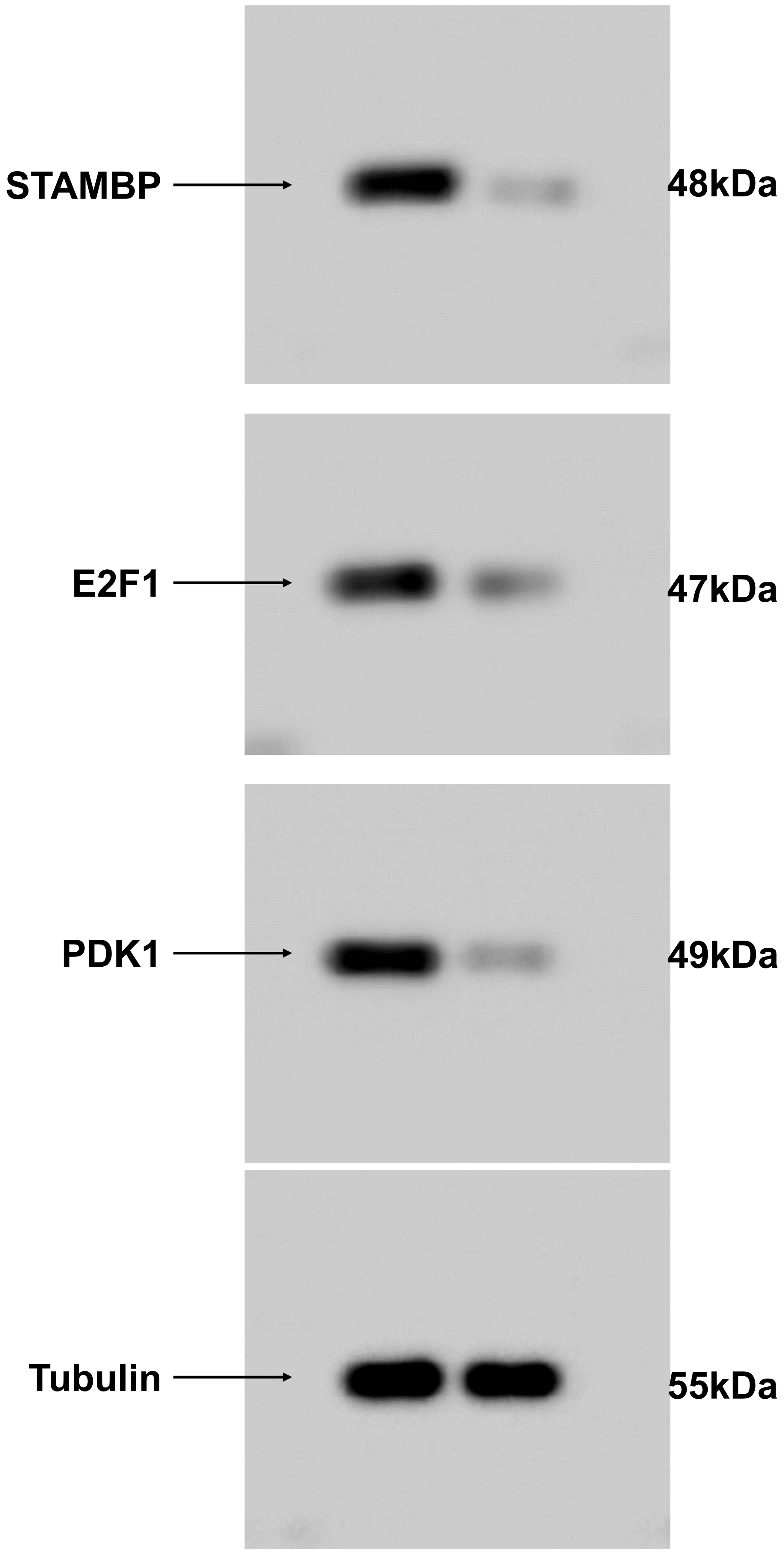

Supplement: Supplementary file 2 — Original Data [file 41419_2024_7048_MOESM2_ESM.pdf]
